# MeCP2 heterochromatin organization is modulated by arginine methylation and serine phosphorylation

**DOI:** 10.3389/fcell.2022.941493

**Published:** 2022-09-12

**Authors:** Annika Schmidt, Jana Frei, Ansgar Poetsch, Alexandra Chittka, Hui Zhang, Chris Aßmann, Anne Lehmkuhl, Uta-Maria Bauer, Ulrike A. Nuber, M. Cristina Cardoso

**Affiliations:** ^1^ Cell Biology and Epigenetics, Department of Biology, Technical University of Darmstadt, Darmstadt, Germany; ^2^ Stem Cell and Developmental Biology, Department of Biology, Technical University of Darmstadt, Darmstadt, Germany; ^3^ Queen Mary School, Medical College, Nanchang University, Nanchang, China; ^4^ Plant Biochemistry, Ruhr University Bochum, Bochum, Germany; ^5^ College of Marine Life Sciences, Ocean University of China, Qingdao, China; ^6^ Division of Medicine, The Wolfson Institute for Biomedical Research, University College London, London, United Kingdom; ^7^ Department of Neuromuscular Diseases, Queen Square Institute of Neurology, University College London, London, United Kingdom; ^8^ Institute of Molecular Biology and Tumor Research, Philipps University Marburg, Marburg, Germany

**Keywords:** arginine (di)methylation, heterochromatin organization, MeCP2, protein arginine methyltransferases, Rett syndrome

## Abstract

Rett syndrome is a human intellectual disability disorder that is associated with mutations in the X-linked *MECP2* gene. The epigenetic reader MeCP2 binds to methylated cytosines on the DNA and regulates chromatin organization. We have shown previously that *MECP2* Rett syndrome missense mutations are impaired in chromatin binding and heterochromatin reorganization. Here, we performed a proteomics analysis of post-translational modifications of MeCP2 isolated from adult mouse brain. We show that MeCP2 carries various post-translational modifications, among them phosphorylation on S80 and S421, which lead to minor changes in either heterochromatin binding kinetics or clustering. We found that MeCP2 is (di)methylated on several arginines and that this modification alters heterochromatin organization. Interestingly, we identified the Rett syndrome mutation site R106 as a dimethylation site. In addition, co-expression of protein arginine methyltransferases (PRMT)1 and PRMT6 lead to a decrease of heterochromatin clustering. Altogether, we identified and validated novel modifications of MeCP2 in the brain and show that these can modulate its ability to bind as well as reorganize heterochromatin, which may play a role in the pathology of Rett syndrome.

## Highlights

1) MeCP2 from mouse brain is methylated on arginines and phosphorylated on serines
2) Phosphorylation on serine 80 increases MeCP2 chromatin binding kinetics
3) Phosphorylation on serine 421 increases chromatin clustering
4) MeCP2 is arginine methylated on R91, R162, R167, and this modulates heterochromatin organization
5) R106 is dimethylated and its mutation results in reduced DNA binding and heterochromatin clustering abilities

## 1 Introduction

The methyl-CpG-binding protein 2 (MeCP2) is the founding member of the methyl-CpG binding domain (MBD) protein family and specifically binds to methylated CpGs *via* its MBD. As DNA methylation is mainly found on the less transcriptionally active heterochromatin, MeCP2 is prominently localized *in vivo* at pericentric chromatin regions, which contain highly methylated major satellite DNA repeats (Lewis et al., 1992). In addition to the MBD, MeCP2 contains a transcriptional repression domain (TRD) (Nan et al., 1997), the interdomain region (ID) and, more recently, the N-CoR/SMRT interacting domain (NID) has also been mapped (Lyst et al., 2013). MeCP2 binds to multiple interaction partners *via* these regions (reviewed in (Schmidt et al., 2020). Several of the interacting partners are components of transcriptional repression complexes, for example Sin3A, HDAC and N-CoR (Jones et al., 1998; Nan et al., 1998; Kokura et al., 2001; Lunyak et al., 2002; Lyst et al., 2013). MeCP2 might also be involved in transcriptional activation as it associates with CREB1 (Chahrour et al., 2008). Aside from the MBD, which shows structurally conserved motifs, MeCP2 was reported to be an intrinsically disordered protein (Adams et al., 2007).

In mouse cells, pericentric heterochromatin from different chromosomes forms densely packed chromatin clusters in interphase called chromocenters ((Baccarini, 1908), see review (Jost et al., 2012)). Increased MeCP2 levels, either occurring during cell differentiation or upon exogenous expression of fusion protein constructs, cause large-scale reorganization of heterochromatin, which can be visualized as fusion events of heterochromatin clusters in mouse cells (Brero et al., 2005; Agarwal et al., 2007; Bertulat et al., 2012). As constitutive heterochromatin has been shown to organize chromosomes within the cell nucleus (Falk et al., 2019), its reorganization has potential impact on the general chromosome distribution. Recently, we and others proposed that heterochromatin cluster fusion events might be mediated by liquid-liquid phase separation (Larson et al., 2017; Strom et al., 2017), as MeCP2 was shown to undergo phase separation under physiological conditions (Fan et al., 2020; Wang et al., 2020; Zhang et al., 2022). MeCP2 shows characteristic properties of phase separating proteins including intrinsically disordered regions and multivalency, and it was reported to interact with itself and several other interaction partners *via* regions outside of the MBD (Becker et al., 2013).

Mutations in the *MECP2* gene were linked to Rett syndrome, a human neurological disorder affecting mainly females, that is associated with intellectual disability among other symptoms (Amir et al., 1999). *MECP2* mutations in males can lead to a wide spectrum of phenotypes ranging from mild intellectual impairment to severe neonatal encephalopathy and premature death (Inuzuka et al., 2021). Missense mutations in the MBD domain of *MECP2* affect heterochromatin accumulation due to reduced DNA binding ability, but also heterochromatin clustering (Agarwal et al., 2011). The clustering function of some mutations could be rescued by retargeting MeCP2 to heterochromatin (Casas-Delucchi et al., 2012).

Importantly, MeCP2 is post-translationally modified and although many modifications have been identified, only a few were validated and functionally characterized (reviewed in (Bellini et al., 2014; Schmidt et al., 2020)). The phosphorylation of serine 421 in the C-terminal domain of MeCP2 was identified upon neuronal activity and stress exclusively in the brain, indicating a specific function under this condition (Zhou et al., 2006; Tao et al., 2009). Serine 80 phosphorylation in the N-terminal domain of MeCP2 was found in mouse and rat brain (Tao et al., 2009). Serine to alanine mutated knock-in mice of both modification sites were reported to display opposing phenotypes, as S421A mice show increased, whereas S80A mice show decreased locomotor activity. In line with these results, membrane depolarization in cortical neurons results in dephosphorylation of serine 80 and phosphorylation of serine 421. Interestingly, the S80A mutation results in a decrease of MeCP2 chromatin binding affinity to *Pomc* and *Gtl2* promoters evaluated by ChIP-qPCR but did not lead to significant changes in gene transcription (Tao et al., 2009). Besides, MeCP2 was found to be poly(ADP-ribosyl)ated in mouse brain tissue at ID and TRD, and this led to decreased DNA binding and heterochromatin clustering (Becker et al., 2016).

In this study, we aimed to identify post-translational modifications of MeCP2 from mouse brain (*in vivo*) and determine whether these modifications are involved in MeCP2 chromatin binding and clustering. 23% of the MeCP2 protein is composed of positively charged amino acids and we found only a few modified arginines compared to many modified lysines. In addition, we identified several phosphorylated serine and threonine residues, including the previously reported S80 and S421. We show that arginine methylation and to a much lesser extent also serine phosphorylation affect heterochromatin accumulation and binding kinetics and MeCP2 heterochromatin clustering function. In addition, coexpression of MeCP2 variants and the protein arginine methyltransferase 6 (PRMT6) reveals differences in heterochromatin clustering.

## 2 Materials and methods

### 2.1 Nuclei isolation from mouse brains

3-month-old C57BL/6 mice (Charles River Laboratories, Inc., Wilmington, MA) were sacrificed according to the animal care and use regulations (Government of Hessen, Germany), and the organs were collected from the sacrificed animals, washed with PBS and frozen in liquid nitrogen. For nuclei isolation the frozen mouse brains were crushed to powder and homogenized in 0.25 M sucrose solution (20 mM triethanolamine-HCl (pH 7.6), 30 mM KCl, 10 mM MgCl_2_, 1 mM DTT, 1 mM PMSF). After centrifugation for 10 min at 1,000 x g, the supernatant was discarded and the pellet resuspended in sucrose buffer to a final sucrose concentration of 2.1 M. The raw nuclei fraction was obtained by ultracentrifugation for 30 min at 50,000 x g. The pellet was resuspended in 0.25 M sucrose solution and centrifuged at 1,000 x g. During the procedure, samples were taken after resuspension of the tissue, after homogenization and after nuclei isolation, fixed with 3.7% formaldehyde in solution for 15 min, dropped on slides, dried and counterstained with 4’,6-diamidino-2-phenylindole (DAPI) for microscopic examination of the individual steps.

### 2.2 Protein enrichment

For the MeCP2 enrichment from mouse brain tissue we made use of its natural hepta-histidine tag for protein pull-down with Ni-IDA beads (His60 Ni Superflow resin, Clontech Laboratories, Inc., Mountain View, CA). First, 10^7^ mouse brain nuclei in PBS were pelleted by centrifugation at 1,000 x g for 10 min. The nuclei were resuspended in buffer B (0.2% Triton X-100, 50 mM triethanolamine-HCl (pH 7.6), 5 mM MgCl_2_), incubated on ice for 10 min and centrifuged at 1,000 x g for 10 min. The supernatant was discarded and the pellet washed three times by resuspension in 100 µl buffer C (2 mM triethanolamine-HCl (pH 7.6), 0.5 mM MgCl_2_) and centrifugation for 10 min at 1,000 x g. The pellet was resuspended in 500 µl 1 M NaCl equilibration buffer (50 mM sodium phosphate, 20 mM imidazole, pH 7.4), followed by sonication 3 × 20 s (250–450 Sonicator, BRANSON ultrasonic corporation, Danbury, CT) with microscopic control after each step. Subsequently, the lysate was diluted using 500 µl equilibration buffer without NaCl and added to the Nickel-Iminodiacetic acid (Ni-IDA) beads for incubation overnight at 4°C with rotation. The beads were washed with 300 mM NaCl equilibration buffer, then with wash buffer (50 mM sodium phosphate, 300 mM NaCl, 40 mM imidazole, pH 7.4). The Ni-IDA beads were then resuspended in Laemmli buffer (2% SDS, 50 mM Tris (pH 6.8), 10% glycerol, 0.01% bromophenol blue, 100 mM DTT), incubated at 95°C for 10 min and separated using sodium dodecylsulfate polyacrylamide gel electrophoresis (SDS-PAGE). The protein enrichment from *E. coli* BL21 (DE3) was performed using the pTYB1-MeCP2wt plasmid coding for MeCP2 with a C-terminal intein-CBD tag allowing protein binding to chitin beads and subsequent elution by cleavage as described before (Zhang et al., 2022).

### 2.3 Mass spectrometry

The samples to be analyzed by mass spectrometry were analyzed by SDS-PAGE and the gel was stained with Coomassie staining solution (5% aluminium sulfate-(14)-(18)-hydrate, 10% ethanol p.a., 0.02% CBB-G250 (Coomassie brilliant blue), 2% orthophosphoric acid (Dyballa and Metzger, 2009)) over night. The in-gel tryptic digestion was performed as described before (Cerletti et al., 2015). Briefly, the gel was destained using Coomassie destaining solution (10% ethanol p.a., 2% orthophosphoric acid, LC-MS grade) two times for 10 min, equilibrated in ddH_2_O (MS grade), the bands of interest were excised, cut to small cubes and dried using a vacuum concentrator. For destaining the gel pieces were covered with destaining solution (40 mM ammonium bicarbonate, 50% acetonitrile, LC-MS grade), incubated at 37°C for 30 min with shaking and the solution was removed. Destaining was repeated at least two times and the gel pieces were dried using a vacuum concentrator. For trypsin digestion the gel pieces were covered with 12.5 ng/μl trypsin (sequencing grade modified trypsin, V5111, Promega Corporation, Madison, WI) in 40 mM ammonium bicarbonate and incubated at 37°C with shaking overnight. The peptides were eluted by adding elution solution (50% acetonitrile, 0.5% trifluoroacetic acid, LC-MS grade), incubation for 20 min in an ultrasonic bath, transfer of the peptide solution to a new tube and drying using a vacuum concentrator. Samples were resuspended in 20 µl buffer (0.1% formic acid in 2% acetonitrile, LC-MS grade), incubated in an ultrasonic bath for 5 min and transferred to HPLC vials. Subsequent drying of the samples in a vacuum concentrator allowed storage at room temperature in the dark until the measurement.

The HPLC-MS/MS measurement was performed with the setup described before (Cerletti et al., 2015). Briefly, an UPLC HSS T3 column and an UPLC Symmetry C18 trapping column for LC were used in combination with the nanoACQUITY gradient UPLC pump system (Waters, Milford, MA) coupled to a LTQ Orbitrap Elite mass spectrometer (Thermo Fisher Scientific, Waltham, MA). The LTQ Orbitrap Elite was operated in a data-dependent mode using Xcalibur software either in collision-induced dissociation (CID) TOP20 or in TOP10 with high-energy collisional dissociation (HCD) and CID fragmentation for every precursor ion. For elution of the peptides a linear gradient from 5%—30% for 60 min (CID TOP20) or 150 min (TOP10 HCD, CID) of buffer B (0.1 formic acid in acetonitrile, UPLC/MS grade) was applied, followed by a step gradient from 30%—85% acetonitrile for 5 min at a flow rate of 400 nl/min.

Data analysis was performed using Proteome discoverer 1.3 (Thermo Fisher Scientific) with SEQUEST (Eng et al., 1994) and MaxQuant (version 2.0.3.0) with Andromeda (Tyanova et al., 2016) algorithms searching against the complete UniProt database (UniProt Consortium, 2021) for *Mus musculus*. A maximum of two missed tryptic cleavages was accepted and methionine oxidation, N-terminal acetylation, N-terminal pyroglutamate, lysine acetylation, lysine ubiquitination, lysine and arginine mono-methylation or di-methylation and serine/threonine/tyrosine phosphorylation were set as variable modifications. To identify all methylation and dimethylation sites, the search was repeated including either only lysine/arginine methylation or dimethylation. The MaxQuant search was run with default parameters having matching between runs enabled.

### 2.4 Plasmids

All plasmids used in this study are listed in Supplementary Table S1. The tetracycline inducible (Tet-On® 3G) pmMeCP2 wt expression plasmid was assembled from several plasmids: pEGFP-N1_MeCP2(WT) was a gift from Adrian Bird (Addgene plasmid #110186; http://n2t.net/addgene:110186; RRID:Addgene_110186) (Tillotson et al., 2017), AAVS1-TRE3G-EGFP was a gift from Su-Chun Zhang (Addgene plasmid #52343; http://n2t.net/addgene:52343; RRID:Addgene_52343) (Qian et al., 2014), HSC1-HS4-GiP was a gift from James Ellis (Addgene plasmid #58540; http://n2t.net/addgene:58540; RRID:Addgene_58540) (Rival-Gervier et al., 2013), pSpCas9(BB)-2A-Puro (PX459) V2.0 was a gift from Feng Zhang (Addgene plasmid #62988; http://n2t.net/addgene:62988; RRID:Addgene_62988) (Ran et al., 2013). The linker peptide as well as additionally required restriction sites were added during PCR amplification using accordingly designed primers (Supplementary Table S2). Three HS4 insulators were inserted flanking the cDNA sequence of *Mecp2-EGFP* under the control of the TRE3G promoter and flanking the Tet-On® 3G expression cassette under control of the CAG promoter to reduce leaky expression of *Mecp2-EGFP*. All cDNA sequences for MeCP2 modification site mutants were generated using overlap extension PCR (Heckman and Pease, 2007) with the primers listed in Supplementary Table S2. Mutant *Mecp2* cDNA sequences (PCR products) were inserted into pmMeCP2G wt to replace WT *Mecp2* cDNA through a cloning step using unique restriction sites of SalI and BamHI. The pTYP1-MeCP2wt plasmid used for bacterial expression was a gift from Christopher L. Woodcock (Georgel et al., 2003).

For the cloning of *hPRMT1*, the cDNA of PRMT1 transcript variant 2 (Goulet et al., 2007) was generated as a gBlock with an artificial nucleotide sequence due to gBlock optimization. The gBlock and pcDNA3.1 vector were digested with HindIII and XhoI and subsequently ligated to obtain the phPRMT1-pcDNA3.1 plasmid. The *mPRMT4* cDNA fragment was amplified by PCR from pSG5-HA_PRMT4 (Chen et al., 1999) and subsequently cloned *via* EcoRI and XhoI into pcDNA3.1. The h*PRMT5* transcript variant 1 (NM_006109) sequence was obtained by PCR using hPRMT5-fwd and hPRMT5-rev primers generating BamHI and EcoRI sites, which enabled cloning of *PRMT5* into pcDNA3.1.

### 2.5 Cell culture and transfection

C2C12 mouse myoblast cells (female), MTF mouse tail fibroblast (male) MeCP2 -/y cells (see Supplementary Table S3) and human embryonic kidney (HEK) 293T cells (female) were grown in Dulbecco’s modified Eagle Medium (DMEM) with high glucose (#D6429, Sigma-Aldrich, St. Louis, MO) supplemented with 20% (C2C12) or 10% (MTF -/y, HEK293T) fetal bovine serum (#F7524, Sigma-Aldrich), 1x glutamine (#G7513, Sigma-Aldrich) and 1 µM gentamicin (#G1397, Sigma-Aldrich) at 37°C and 5% CO_2_ in a humidified incubator. *Mycoplasma* tests were performed regularly and all cell lines are listed in Supplementary Table S3. C2C12 cells were tested for the ability to differentiate to myotubes, MTF -/y cells were proven to be MeCP2 negative by immunofluorescence staining and HEK293T cells were authenticated by STR profiling.

Transient transfections of C2C12 and MTF -/y cells were performed using the Neon transfection System (Thermo Fisher Scientific, Waltham, MA) according to the manufacturer’s instructions. For cotransfections of MeCP2 mutants and PRMTs a plasmid amount ratio of 1:5 (2 μg, 10 µg) was used. After transfection, cells were seeded on gelatin-coated coverslips and grown at 37°C and 5% CO_2_ in a humidified incubator. 7 h after transfection, cells were washed with PBS and transcription was induced by adding medium supplemented with 2 µM tetracycline. 20 h after induction, cells were washed with PBS and fixed either with ice cold methanol for 6 min (MTF -/y) or with 3.7% formaldehyde (C2C12) for 15 min. HEK293T cells were transfected using polyethylenimine (PEI, Sigma-Aldrich) as described previously (Agarwal et al., 2007).

### 2.6 Protein salt extraction

C2C12 cells were transfected with plasmids expressing MeCP2-WT, S80D, or 3L and treated 7 h with 1 μg/ml tetracycline followed by incubation for 20 h. The cells were harvested by trypsinization, washed using PBS, counted, and aliquoted into four tubes with the same cell number. Cells were resuspended in 100 µl lysis buffer (20 mM Tris-HCl, pH 8.0, 0.5 mM EDTA, 0.5% NP-40, protease inhibitors, and 150, 450, 600, or 1,000 mM NaCl separately). Cells were lysed by syringe treatment (21G needle, 20 strokes), followed by 25 min incubation on ice. The lysate was collected by centrifugation at 4,600 × g for 15 min at 4°C. The pellet was dissolved in 2% SDS in water. Both lysate and pellet were mixed with Laemmli buffer, and boiled at 95°C for 5 min before Western blot analysis.

### 2.7 Western blot analysis

Mouse brain nuclei were lysed in Laemmli buffer (see above), mechanically disrupted and incubated for 5 min at 95°C. The samples were analyzed by SDS-PAGE and transferred to a nitrocellulose membrane using a semi-dry blotting system at 25 V for 35 min. For detection of post-translational modifications, membranes were blocked with 5% BSA in TBS-T (0.1% Tween 20 in TBS) for 1 h and incubated with anti-PTM antibody diluted in TBS-T (antibodies and dilutions are listed in Supplementary Table S4) at 4°C with shaking overnight. Membranes were washed three times with TBS, blocked for 30 min with 5% low-fat milk in PBS and incubated overnight with anti-MeCP2 monoclonal rat antibody mix (4H7, 4G10, 4E1 undiluted, (Jost et al., 2011)). After three washing steps with 0.1% PBST (0.1% Tween 20 in PBS), membranes were incubated with Cy3-conjugated anti-rat IgG secondary antibody diluted 1:1,000 in 3% milk in PBS for 1 h, washed three times with PBST and the fluorescent signal for MeCP2 was detected using Amersham AI600 imager (see Supplementary Table S5). Subsequently, membranes were incubated with HRP-coupled secondary antibodies (either rabbit or mouse IgG) diluted in 3% milk for 1 h, washed three times with PBST, stained with ECL solution (Clarity Western ECL substrate, #1705061, Bio-Rad, Hercules, CA) and the chemiluminescence signal for the PTMs detected using an Amersham AI600 imager.

All other Western blots were performed similarly to MeCP2 detection. Briefly, membranes were blocked for 30 min with 5% low-fat milk in PBS, incubated with primary antibody diluted in 5% low-fat milk overnight at 4°C shaking, washed three times with PBST, incubated 1 h at room temperature with secondary antibody diluted in 3% low-fat milk, washed three times with PBST and signals were detected using an Amersham imager.

### 2.8 Immunofluorescence staining

After fixation either with ice cold methanol for 6 min (MTF -/y) or with 3.7% formaldehyde (C2C12) for 15 min and washing with PBS, cells were permeabilized with 0.7% Triton X-100 in PBS for 20 min and washed three times with 0.01% PBST. Cells were either directly stained with DAPI or blocked with 2% BSA in PBST for 20 min and incubated with primary antibody diluted in 2% BSA in PBST for 2 h (primary and secondary antibodies with their respective dilutions are listed in Supplementary Table S4). After washing three times with 0.1% PBST, secondary antibody in 2% BSA was applied for 1 h in the dark, followed by three times washing with 0.1% PBST, 12 min DAPI staining in the dark, washing with PBST and water and mounting in Mowiol 4-88 (#81381, Sigma-Aldrich; 4.3 M Mowiol 4-88 in 0.2 M Tris-HCl pH 8.5 with 30% glycerol) supplemented with 2.5% DABCO antifade (1,4-diazabicyclo (2.2.2)octan, #D27802, Sigma-Aldrich).

### 2.9 Fluorescence microscopy and image analysis

All characteristics of the microscopy systems used including lasers/lamps, filters, objectives, detection and incubation systems are listed in Supplementary Table S5.

#### 2.9.1 Microscopic analysis of subcellular localization

Fluorescence and DIC images of transfected C2C12 cells were taken using a Nikon Eclipse TiE2 system with a 40x air Plan Apo *λ* DIC objective. Fluorescence images of transfected MTF -/y cells were acquired using a confocal microscope Leica TCS SPEII and intensities were measured using ImageJ (https://imagej.nih.gov/ij/).

#### 2.9.2 Microscopic analysis of heterochromatin accumulation

Fluorescence images of transfected C2C12 cells for calculation of heterochromatin accumulation were acquired on a Zeiss Axiovert 200 microscope. Image segmentation was performed using an ImageJ macro described previously (Zhang et al., 2022). First, the cell nuclei were segmented semi-manually based on the DAPI intensity and a difference of gaussian blur filter was applied. For heterochromatin segmentation individual pixel intensities were calculated, local maxima were determined in squares of 30 × 30 pixels and pixel intensities were binned into 42 bins with the local maximum defining the intensity of the highest bin per square. Heterochromatin masks were obtained by thresholding the pixel intensities based on their respective bins, taking all pixels with bins ≥21 for total, ≥37 for core heterochromatin cluster regions. The nucleoplasm area was calculated by subtracting the total heterochromatin cluster areas from the nucleus area. The heterochromatin accumulation of MeCP2 mutants for each individual heterochromatin cluster was calculated by dividing the mean intensity of the heterochromatin cluster core by the mean intensity of the nucleoplasm (see Supplementary Figure S1). To analyze the protein level-dependency of accumulation differences, the cells were divided into low and high protein levels based on their GFP intensity as described before (Zhang et al., 2022). Briefly, the log10 GFP sum intensity of the cells was plotted and divided into 40 bins. In comparison to the GFP intensity of untransfected cells, bins 1 to 11 were defined as negative, while cells in bins 13 to 21 were considered as low and cells in bins 24 to 32 as high expressing.

#### 2.9.3 Microscopic analysis of heterochromatin clustering

High-content screening microscopy of transfected C2C12 cells was performed using a PerkinElmer Operetta imaging system and analyzed using the supplier’s software Harmony (Version 3.5.1, PerkinElmer Life Sciences, United Kingdom). Briefly, cell nuclei were segmented based on the DAPI channel image considering nuclei with a size of 110–250 μm^2^ and roundness coefficient >0.8 that are not touching the edges of the image. Heterochromatin segmentation was also performed using the DAPI channel image, identifying high intensity spots within the nuclei. Spots with a spot-to-region intensity ratio of at least 0.35, an area of at least five px^2^ and a relative spot intensity of 0.0253 were considered. The nucleus and heterochromatin masks generated based on the DAPI channel image were used to segment the images of the other channels and intensity and morphology properties of nuclei and heterochromatin clusters were measured (see Supplementary Figure S2). MeCP2 intensity bins based on GFP intensity were defined as described for the Axiovert images. Not all images obtained were considered for analysis, as the cell numbers per replicate and condition were different caused by differing transfection efficiencies. Thus, either the number of images (three biological replicates) was reduced to achieve comparable cell numbers or the number of cells (two biological replicates) was adjusted to achieve exactly the same number of cells per condition and replicate.

For the cell cycle analysis, the frequencies of the DAPI sum intensities per nucleus were plotted as histogram. The DAPI intensities of the different samples were normalized as described before (Heinz et al., 2018). The intervals for G1, S and G2/M phase were set manually and the percentages of the cells within each interval were plotted as bar diagrams.

#### 2.9.4 Fluorescence recovery after photobleaching analysis of heterochromatin binding kinetics

Live cell imaging for fluorescence recovery after photobleaching (FRAP) experiments was carried out on a confocal microscope Leica SP5 II with a HCX PL APO ×63 oil lambda blue objective equipped with an ACU live cell chamber at 37°C, 5% CO_2_ and 60% humidity. MTF -/y cells were transfected with MeCP2 mutation constructs, seeded on gelatin-coated glass bottom p35 plates followed by induction with 1 µM tetracycline 7 h later and DNA staining with 100 nM SiR-DNA (SiR-Hoechst) (#SC007, Spirochrome) in presence of 5 µM verapamil (included in #SC007). 16 h later heterochromatin clusters were bleached with a 488 nm argon laser at 100% intensity for 2 s and confocal images were taken with a frame size of 256 × 256 with 200 Hz and a pinhole of one AU in time intervals of 1.5 s. For analysis the image series were registration corrected using ImageJ plugin StackReg (correction based on GFP channel) or HyperStackReg (correction based on DNA staining). The mean fluorescence intensities of the (pre- and post-bleach) bleached and unbleached region were background subtracted for each time point. For single normalization, intensities were normalized to the mean of the prebleach intensities. Fluorescence recovery curves were fitted in ImageJ and t-half values and mobile fraction were obtained from the fitted curves. At least 10 cells were analyzed for each construct and the means of the fitted curves, t-half values and mobile fractions were plotted.

#### 2.9.5 Protein *in situ* extractions

The protein extractability was measured in live cells. In brief, the C2C12 cells were transfected with plasmids expressing MeCP2-WT, S80D, or 3 L, plated onto µ-Slide eight Well plate (#80826, Ibidi), and treated 7 h with 1 μg/ml tetracycline followed by incubation for 20 h. Live-cell imaging was performed on an UltraVIEW VoX spinning disc system (PerkinElmer Life Sciences) mounted on a Nikon Ti microscope equipped with an oil immersion 60 Plan-Apochromat NA 1.45 objective lens at 37°C, 5% CO_2_. Confocal z-stacks were acquired at 30 s intervals for 20 min. Z-stack images were taken first in PBS/EDTA and then in PBS with 0.5% Triton X-100 for 20 min with 30 s intervals. Quantifications were performed using Volocity (PerkinElmer Life Sciences). The mean fluorescence intensity signal at the heterochromatin clusters/cell at each time was calculated and normalized to the mean fluorescence intensity of heterochromatin clusters before Triton X-100 treatment.

## 3 Results

### 3.1 MeCP 2 is post-translationally modified in mouse brain

**FIGURE 1:**
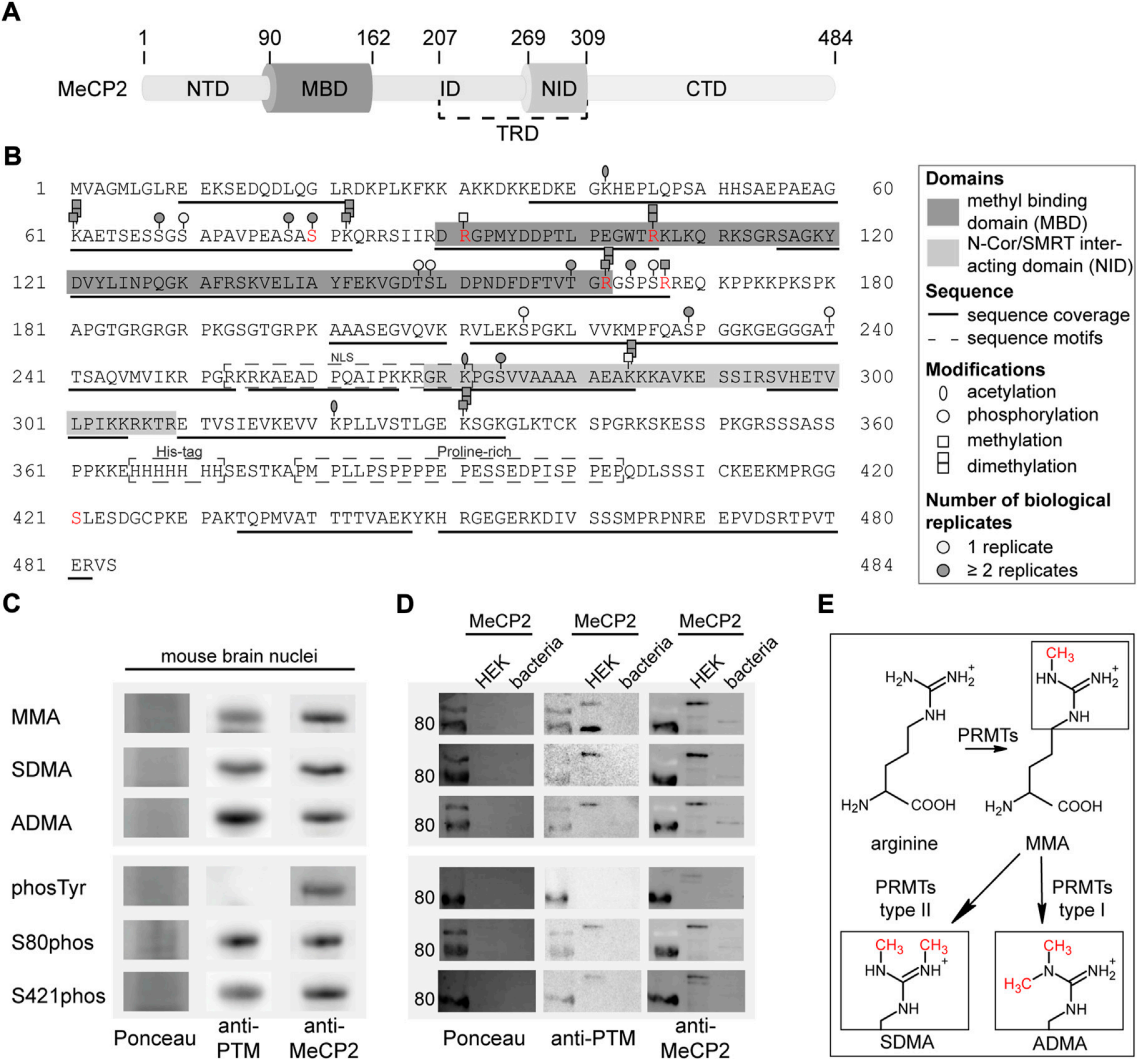
Post-translational modifications of MeCP2 from mouse brain identified by mass spectrometry analysis. **(A)** MeCP2 domain structure comprising N-terminal domain (NTD), methyl-CpG binding domain (MBD), intervening domain (ID), N-CoR/SMRT interacting domain (NID), transcriptional repression domain (TRD) and C-terminal domain (CTD). **(B)** MeCP2 protein sequence with modifications identified by mass spectrometry. MBD and NID, sequence coverage, sequence motifs, PTMs (acetylation, phosphorylation, methylation, dimethylation) and the number of biological replicates from independent experiments are marked as indicated. Modifications selected for further validation are marked in red. The software-based annotation of spectra and modification sites shown were obtained from Proteome Discoverer and MaxQuant. The arginine methylation sites selected for further validation were additionally manually inspected (see Supplementary Figures S4–6). The location of modifications identified on the same peptide might be uncertain. **(C)** Western blot analysis of mouse brain nuclei extracts tested for monomethyl arginine (MMA), symmetric dimethyl arginine (SDMA), asymmetric dimethyl arginine (ADMA), tyrosine phosphorylation (phosTyr), S80 and S421 phosphorylation (phos) and reprobed with MeCP2 specific antibodies. The full membranes of the Western blots are shown in Supplementary Figure S7. **(D)** Western blot analysis of MeCP2-GFP purified from human HEK cells and MeCP2 purified from *E. coli* probed with the same modification specific antibodies and reprobed with MeCP2 specific antibodies. The Ponceau stain visualizes the total proteins on the membrane and the full membranes are shown in Supplementary Figure S7. **(E)** Scheme of the arginine methylation reaction: protein arginine methyltransferases (PRMTs) can catalyze the monomethylation of arginine and subsequently the dimethylation, which can occur either on the same nitrogen (ADMA, catalyzed by type I PRMTs) or on the unmodified nitrogen (SDMA, catalyzed by type II PRMTs).

To identify post-translational modifications (PTMs) of MeCP2, we first isolated nuclei from mouse brain tissue using a sucrose buffer in combination with ultracentrifugation. Then, we enriched MeCP2 from mouse brain nuclei using its natural hepta-histidine sequence localized in its C-terminal domain (see Figures 1A,B) for protein pull down with Ni-IDA beads (Supplementary Figure S3). Subsequently, the enriched proteins were separated by SDS-PAGE, in-gel digested using trypsin and subjected to mass spectrometry analysis using a TOP10 shotgun method with a combination of HCD and CID fragmentation for improved sequence coverage and modification identification. The mass spectrometry analysis of MeCP2 from mouse brain yielded a sequence coverage of 60.1% and a series of PTMs, including lysine methylation and acetylation, arginine methylation and phosphorylation on serines and threonines. Figure 1B depicts the MeCP2 protein sequence with the modifications identified. The peptides on which the modifications were identified and the number of identifications obtained from automated data analysis using either Proteome Discoverer or MaxQuant software are listed in Supplementary Table S7. We decided to functionally characterize especially arginine methylation sites, as 7% of the MeCP2 amino acids are arginines, but we found only 11.4% of the arginines modified. In addition, a large number of *MECP2* mutations identified in Rett syndrome patients affect arginine residues. The arginine methylation sites identified on MeCP2-E2 isoform (starting in exon 2) namely R91, R106, R162, and R167 were selected for further analysis and were validated by manual inspection of the spectra (see Supplementary Figures S4–6). The arginines R162 and R167 are located on the same peptide and were both identified as monomethylated and R162 also as dimethylated in the automated analysis. It was not possible to unambiguously determine the localization of the methylation sites on this peptide from the spectra (see Supplementary Figures S4–6).

As the MeCP2 sequence comprises 13.2% of lysines and 7% of arginines, it is likely that some regions could not be covered in our measurements due to the generation of very short peptides by trypsin which cuts after lysine and arginine. In addition, there is a long sequence without any lysines and arginines in the C-terminus of MeCP2, containing the hepta-histidine sequence and a proline-rich region (see Figure 1B) that was, thus, not accessible. Of note, it was reported that post-translational modifications like methylation lower the efficiency of trypsin mediated cleavage. Therefore, we tried to increase sequence coverage using other enzymes for digestion but were unable to cover the missing regions (data not shown).

The results were validated by immunoblot detection of mono and dimethyl arginine, serine/threonine/tyrosine phosphorylation as well as serine 80 and serine 421 phosphorylation on mouse brain nuclei extracts (Figure 1C; Supplementary Figure S7). The same membranes were incubated with anti-MeCP2 antibody to validate that the PTM signal was specific to MeCP2. We could show that MeCP2 is monomethylated as well as symmetrically and asymmetrically dimethylated on arginines. In addition, it is phosphorylated on serines, but not on tyrosines. The published MeCP2 phosphorylation sites S80 and S421 (Zhou et al., 2006; Tao et al., 2009) were also detected on MeCP2 isolated from mouse brains (Figure 1C). In addition, recombinant MeCP2-GFP was enriched from human HEK cells and MeCP2 from *E. coli* to confirm the antibody specificity by Western blot (Figure 1D). Figure 1E shows a scheme of the arginine methylation reaction catalyzed by protein arginine methyl transferases (PRMTs). First, arginine residues can be monomethylated by PRMT enzymes of class I and II, subsequent asymmetric dimethylation is catalyzed by PRMT enzymes type I, symmetric dimethylation by PRMT enzymes of type II. Of note, the arginine keeps its charge in the methylated state, but shows a different charge distribution due to the bulky methylation groups.

### 3.2 Arginine methylation and serine phosphorylation site mutations do not influence MeCP2 subcellular localization with exception of MeCP2 R106 mutations

To investigate the functional consequences of MeCP2 PTMs, we generated recombinant MeCP2 proteins tagged with GFP and altered at PTM sites. While most published work utilizes the amino acid substitutes aspartate (D) to mimic phosphorylation or alanine (A) to prevent phosphorylation, there are no commonly used substitutions for methylated arginines. Thus, we decided to mutate the arginines identified to be methylated to lysine (K) to retain the positive charge, to glutamine (Q) to sterically mimic a methylated arginine and to leucine (L) to obtain methyl groups similar to a methylated arginine (compare Supplementary Figure S8C and Figure 1E). In addition, arginine substitutions to glutamine and leucine were identified in Rett syndrome patients. Of note, none of these mutations is an ideal mimic for methylated or unmethylated arginine. In methylated arginine the positive charge of the arginine is kept with the addition of the sterically hindering additional methyl group(s). These amino acid substitutions can, thus, only partially mimic these changes, either the positive charge (K), the methyl group (L) or a polar and sterically larger side chain (Q). MeCP2 arginine R106 was detected as methylated in our proteomic screen (Figure 1B) and is found mutated to tryptophan (R106W) or glutamine (R106Q), with very low frequency also to glycine (R106G) and leucine (R106L) in Rett syndrome patients (online RettBASE, (Krishnaraj et al., 2017)). We therefore generated recombinant MeCP2 with the R106 mutated to produce lysine, glutamine, leucine, tryptophan and glycine, thus including the previously explained substitutions for arginine methylated sites (K, Q, and L) and all reported R106 Rett syndrome mutations (W, Q, G, and L).

**FIGURE 2:**
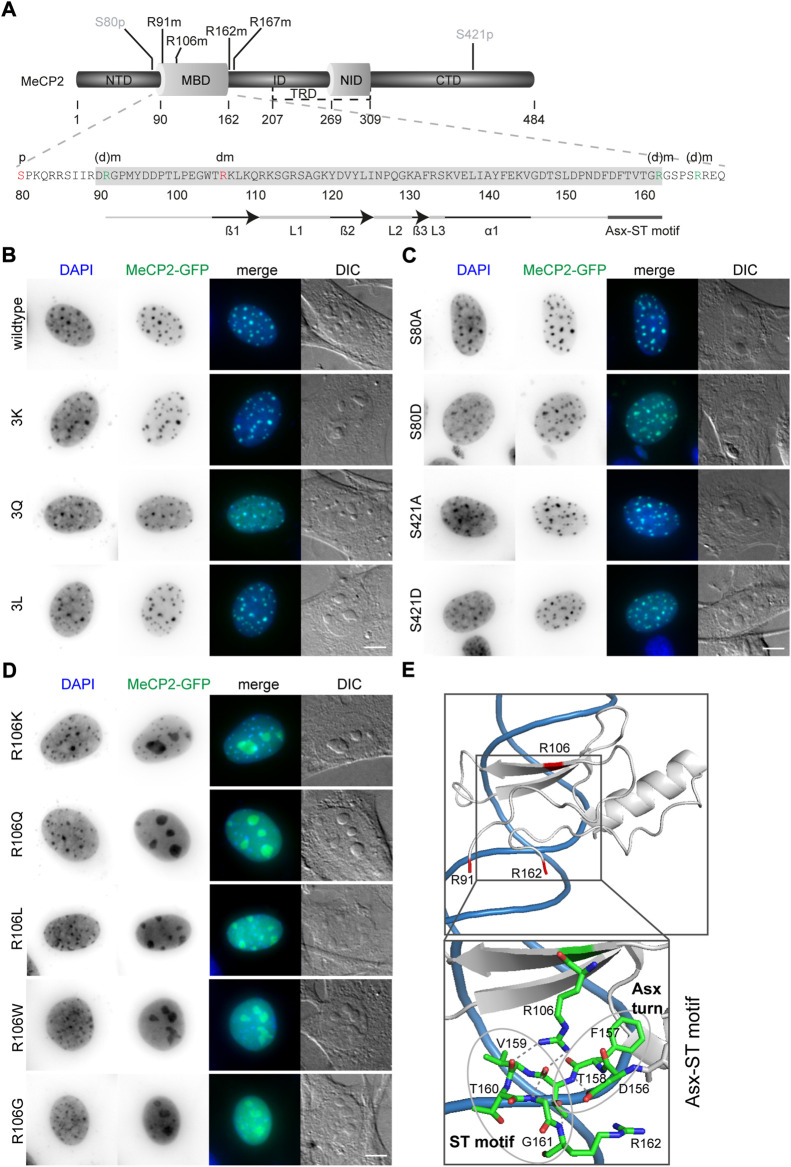
Subcellular localization of MeCP2-GFP mutant constructs transfected in C2C12 mouse myoblast cells. **(A)** MeCP2 domain structure and MBD amino acid sequence with the modification sites selected for functional validation indicated in red (S80phos, R106dimet) or green (R91, R162, and R167, referred to as 3x). **(B–D)** MeCP2 wild type and 3K, 3Q, and 3L mutations **(B)** as well as S80 and S421 mutations **(C)** colocalized with DAPI dense heterochromatic regions, whereas R106 mutations lose their DAPI colocalization and mislocalized to the nucleoli **(D)**. Scale bar 5 µm. **(E)** X-ray structure of the MeCP2 methyl-binding domain (MBD, shown in gray) in complex with methylated DNA (shown in blue) with the methylated arginine sites R91, R106, and R162 highlighted in red (structure information from (Ho et al., 2008); PDB accession code 3C2I). The enlarged image shows the Asx-ST motif stabilizing the MBD binding to methylated DNA. The Asx turn composed of D156, F157, and T158 is stabilized by a hydrogen bond between the carboxylate side chain of D156 and T158 main chain nitrogen. The ST motif of the amino acids 158 to 161 comprises two hydrogen bonds, one between the side chain hydroxyl group of T158 and the main chain nitrogens of G161 and R162, the second one between the main chain carbonyl group of T158 and G161 main chain nitrogen (Ho et al., 2008). The structural data was generated and color-coded using PyMOL software.

The MeCP2-GFP plasmids point mutated for modified sites (Figure 2A) were transfected into male mouse tail fibroblasts MTF-/y (Supplementary Figures S8A,B), which are MeCP2 null cells, and C2C12 female myoblast cells (Figures 2B–D), which have a very low to undetectable level of MeCP2 (Zhang et al., 2022) and, thus, can be used as a functional MeCP2-null system. In the following, the constructs are abbreviated as: 3K (triple R91K R162K R167K), 3Q (triple R91Q R162Q R167Q), and 3L (triple R91L R162L R167L).

First, we analyzed the subcellular localization of the altered MeCP2 proteins and compared them with the wild type MeCP2 protein. Like wild type MeCP2, the MeCP2 proteins with 3K, 3Q, and 3L substitutions (Figure 2B, Supplementary Figure S8A) as well as all the phosphorylation site altered proteins (Figure 2C, Supplementary Figure S8B) were enriched at heterochromatin, visualized as dense DAPI stained DNA regions in the images. All R106 altered MeCP2 proteins, as reported earlier for MeCP2 R106W and R106Q (Ballestar et al., 2000; Yang et al., 2016), tend to lose their heterochromatin enrichment and mislocalize to the negatively charged RNA-enriched nucleolar compartment (Figure 2D). The nucleolar compartment is visualized in the DIC images where it appears as prominent large structures within the cell nucleus. Interestingly, it was described that peptides with high occurrence of arginines tend to localize at the negatively charged nucleoli (Martin et al., 2015). While MeCP2 R106 K still shows some heterochromatin localization, the other R106 mutant proteins localized nearly exclusively within the nucleoli and showed higher intensities in the nucleoplasm compared to the wild type (Figure 2D). These results might be explained by the role of R106 in MBD binding to the DNA. It was reported that MeCP2 binding to methyl-CpG on the DNA is mediated by direct contact of the three amino acids D121, R111, and R133 and might involve five water molecules (Ho et al., 2008). Arginine 106 stabilizes the Asx-ST motif, a motif stabilizing MeCP2 DNA interaction ((Ho et al., 2008), see Figure 2E). Interestingly, also the frequent Rett syndrome missense mutation T158M is localized in this motif. Both missense mutations occur very frequently and reduce DNA binding, emphasizing the importance of this motif for proper methyl-CpG binding and MeCP2 function.

### 3.3 MeCP2 arginine methylation and serine phosphorylation site mutants accumulate differently in heterochromatin

**FIGURE 3:**
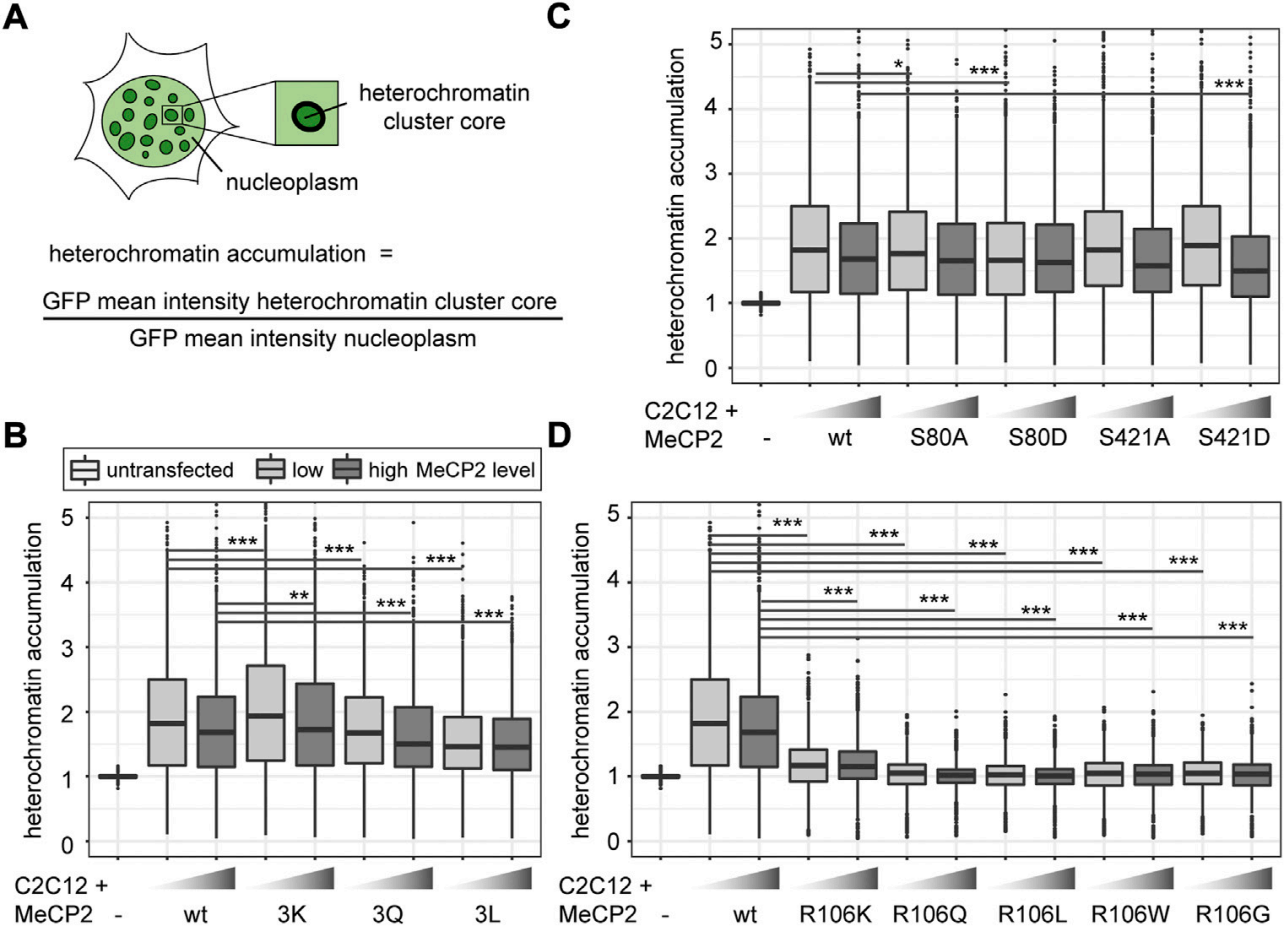
Comparative analysis of heterochromatin accumulation of MeCP2 mutant constructs transfected in C2C12 mouse myoblast cells. **(A)** Heterochromatin accumulation was calculated as the ratio of GFP mean intensity at heterochromatin versus nucleoplasm. The boxplots depict the heterochromatin accumulation of mutant MeCP2 including R91, R162, and R167 **(B)**, S80 and S421 **(C)**, and R106 **(D)** constructs for low and high MeCP2 levels in the cells. Three biological replicates, statistical significance calculated using Wilcoxon-Rank test. *p < 0.05, **p < 0.005, ***p < 0.001. *p*-values and n-values are summarized in Supplementary Table S6.

To quantitatively analyze heterochromatin accumulation of the MeCP2 mutant constructs, we performed a cellular DNA/chromatin binding assay. C2C12 cells were transfected with the mutant constructs, fixed and counterstained with the DNA dye DAPI. After imaging the cells, the nuclei were segmented semi-manually and heterochromatin compartments were segmented using a self-made macro in ImageJ/Fiji [(Zhang et al., 2022), Supplementary Figure S1]. By calculating the ratio of the mean GFP intensity in the heterochromatin to the mean GFP intensity in the nucleoplasm, we obtained heterochromatin accumulation values (Figure 3A). As MeCP2 levels might have an influence on its degree of heterochromatin accumulation, the cells were classified into low and high MeCP2 levels according to their mean nuclear fluorescence intensity as described before (Zhang et al., 2022).

The quantitative analysis of heterochromatin accumulation revealed a slightly higher accumulation of MeCP2 3K than MeCP2 wild type. MeCP2 3Q showed a lower accumulation than wild type MeCP2 and MeCP2 3L an even lower accumulation compared to the MeCP2 3Q (Figure 3B). The heterochromatin accumulation values of the phosphorylation site mutants were all very similar, with only the phospho mimic mutants S80D showing a slightly lower accumulation at low levels and S421D a lower accumulation at high levels (Figure 3C). In line with the subcellular localization, the heterochromatin accumulation was drastically reduced in all R106 mutants (Figure 3D). Only R106K still showed some heterochromatin accumulation with a ratio clearly above one. Overall, all constructs show a lower accumulation in case of high protein levels, which might hint to a saturation effect of MeCP2 binding to chromatin at high protein levels.

### 3.4 MeCP2 arginine methylation and serine phosphorylation site mutations affect its heterochromatin binding kinetics

**FIGURE 4:**
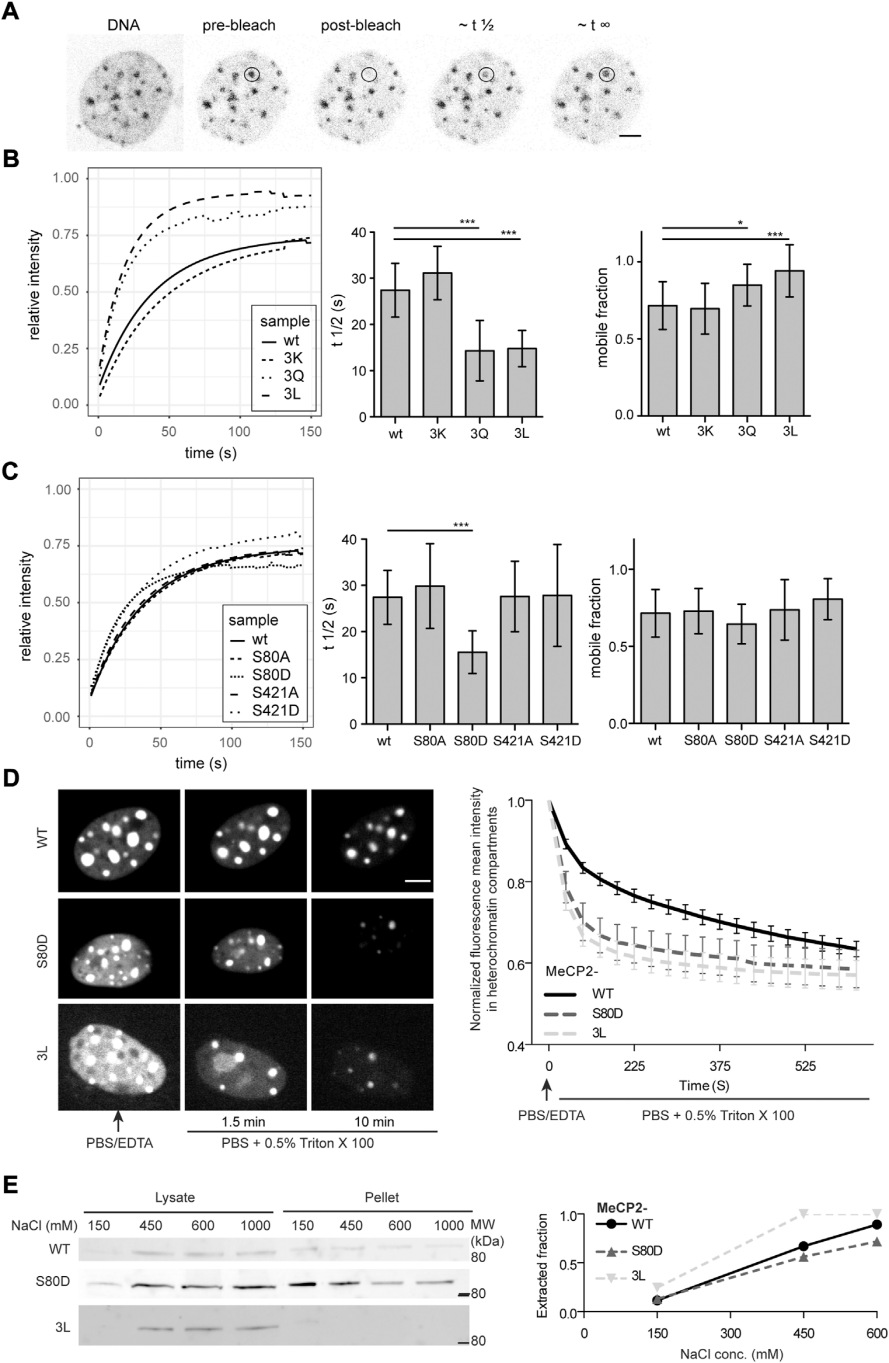
Analysis of the dynamics of MeCP2 mutants in cells. **(A)** Exemplary MTF *Mecp2* -/y cell transfected with wild type MeCP2 expression construct stained with the cell-permeable DNA dye SIR Hoechst and GFP signal is shown pre-bleaching, post-bleaching and during fluorescence recovery. **(B)** Fitted time curves for fluorescence recovery after photobleaching and bar diagrams showing the recovery half times (t ½) and mobile fractions for MeCP2 wild type (wt) and 3K, 3Q, and 3L mutations including R91, R162, and R167 **(B)** as well as for S80 and S421 mutations **(C)**. *p*-values calculated by Wilcoxon-Rank test. **p < 0.05, ***p < 0.001. *p*-values and n-values are summarized in Supplementary Table S6, single recovery curves with standard deviation are plotted in Supplementary Figure S9. **(D)** *In situ* extraction of MeCP2 mutants expressed in C2C12 mouse cells. Shown are representative images at selected time points (left side) and the quantification curve (right side) depicting the mean and SEM. **(E)** C2C12 cells expressing the constructs as indicated were subjected to fractionation with different salt concentrations to ascertain chromatin binding and soluble fraction (lysate) as well as insoluble fraction (pellet) were loaded and probed with anti-MeCP2 antibodies. The plot depicts the ratio of the lysate intensity to the total intensity (sum of lysate plus pellet). Full blots are shown in Supplementary Figure S10. Scale bars 5 µm.

Next, we wanted to know whether the MeCP2 modification site mutants show differences in heterochromatin binding kinetics. Therefore, MTF-/y cells were transfected with the different constructs and heterochromatin compartments were photobleached using a focused laser microbeam on a confocal microscope. The fluorescence recovery was measured by taking images before and every 1.5 s after photobleaching (Figure 4A). Curve fitting of the intensity values over time allowed for calculation of fluorescence recovery half times and mobile fractions (Figure 4, Supplementary Figure S9).

The comparison of the recovery half times of wild type MeCP2 with those of the triple mutants revealed that the MeCP2 3Q and 3L mutants recover much faster than the wild type (Figure 4B). The MeCP2 3K mutant, retaining the positive charge, showed similar kinetics as wild type MeCP2, emphasizing the importance of the positive charge for chromatin binding. These results are in line with the heterochromatin accumulation results, which depicted a slightly higher accumulation for 3K, but a lower one for 3Q and 3L constructs compared to the wild type (Figure 3B). For the phosphorylation mutants only S80D showed faster recovery kinetics compared to wild type MeCP2, but no significant changes in the mobile fraction (Figure 4C). This result also agrees with the heterochromatin accumulation data (Figure 3C). The recovery data for 3L and S80D were validated with *in situ* extraction analysis and by lysing the cells with increasing salt concentrations followed by Western blot analysis of the soluble and insoluble fractions (Figures 4D,E, Supplementary Figure S10). As the R106 mutants were shown to hardly localize or accumulate at heterochromatin, their recovery times were too fast to be measured under similar conditions as wild type MeCP2 and were, thus, not analyzed.

### 3.5 Arginine methylation and serine phosphorylation site mutations of MeCP2 influence its heterochromatin clustering

**FIGURE 5:**
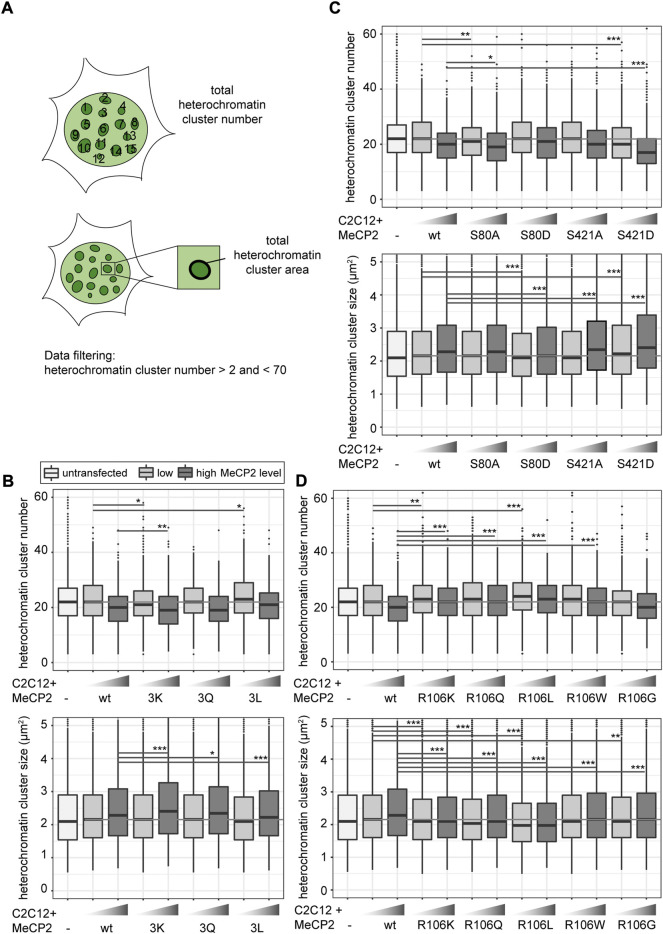
Heterochromatin clustering and cluster size of cells transfected with MeCP2 mutant constructs. Shown are the heterochromatin cluster numbers and areas scheme in **(A)** obtained for the arginine mutants including R91, R162, and R167 **(B)**, arginine R106 **(D)** and S80, S421 phosphorylation **(C)** mutant constructs in C2C12 cells from high-content screening microscopy after segmentation of nuclei and heterochromatin clusters. Cells were divided into low and high MeCP2 levels based on their nuclear GFP signal. Three biological replicates, statistical significance calculated using Wilcoxon-Rank test. *p < 0.05, **p < 0.005, ***p < 0.001, n. s Not significant. *p*-values and n-values are summarized in Supplementary Table S6.

To investigate the influence of MeCP2 modifications on chromatin organization, we performed a cellular chromatin clustering assay. As reported before, many small heterochromatin clusters tend to fuse to build fewer bigger clusters with increasing MeCP2 protein levels and cellular differentiation (Brero et al., 2005). Thus, we aimed to analyze the cellular heterochromatin clustering in two ways, by observing the heterochromatin cluster number and the corresponding area (Figure 5A), which should develop in an inverse manner. The transfected C2C12 cells were imaged on a high-content screening microscope and nuclei and heterochromatin clusters were segmented (Supplementary Figure S2). We confirmed transfected cell viability by cell cycle profiling (Supplementary Figure S10). Depending on the GFP mean nuclear intensity, cells were classified into low and high MeCP2 levels and heterochromatin cluster numbers and areas were plotted (Figure 5).

In comparison to wild type MeCP2, MeCP2 3K showed a higher heterochromatin clustering function represented by lower cluster numbers and larger cluster areas. MeCP2 3L showed a tendency to reduced heterochromatin clustering, while the results with MeCP2 3Q were not significantly different (Figure 5B). These findings correlate well with those obtained for heterochromatin accumulation and heterochromatin binding kinetics. 3K showed higher heterochromatin accumulation, while 3Q and 3L showed lower accumulation and faster heterochromatin binding kinetics (Figures 3B, 4B).

Regarding the phosphorylation site mutants, S421D showed the most striking clustering function difference to wild type MeCP2 represented by lower cluster numbers and larger cluster areas (Figure 5C). In the other functional assays, though, MeCP2 S421D showed lower accumulation than wild type at high protein levels but did not show any significant differences in the binding kinetics (Figures 3C, 4C). Hence, it is unclear how this amino acid substitution affects heterochromatin binding in relation to heterochromatin organization.

For MeCP2 arginine 106, all mutant constructs tested were associated with higher heterochromatin cluster numbers and smaller cluster areas than wild type MeCP2 (Figure 5D), possibly because of their lack of heterochromatin accumulation.

### 3.6 Protein arginine methyltransferases affect MeCP2 induced heterochromatin remodeling

**FIGURE 6:**
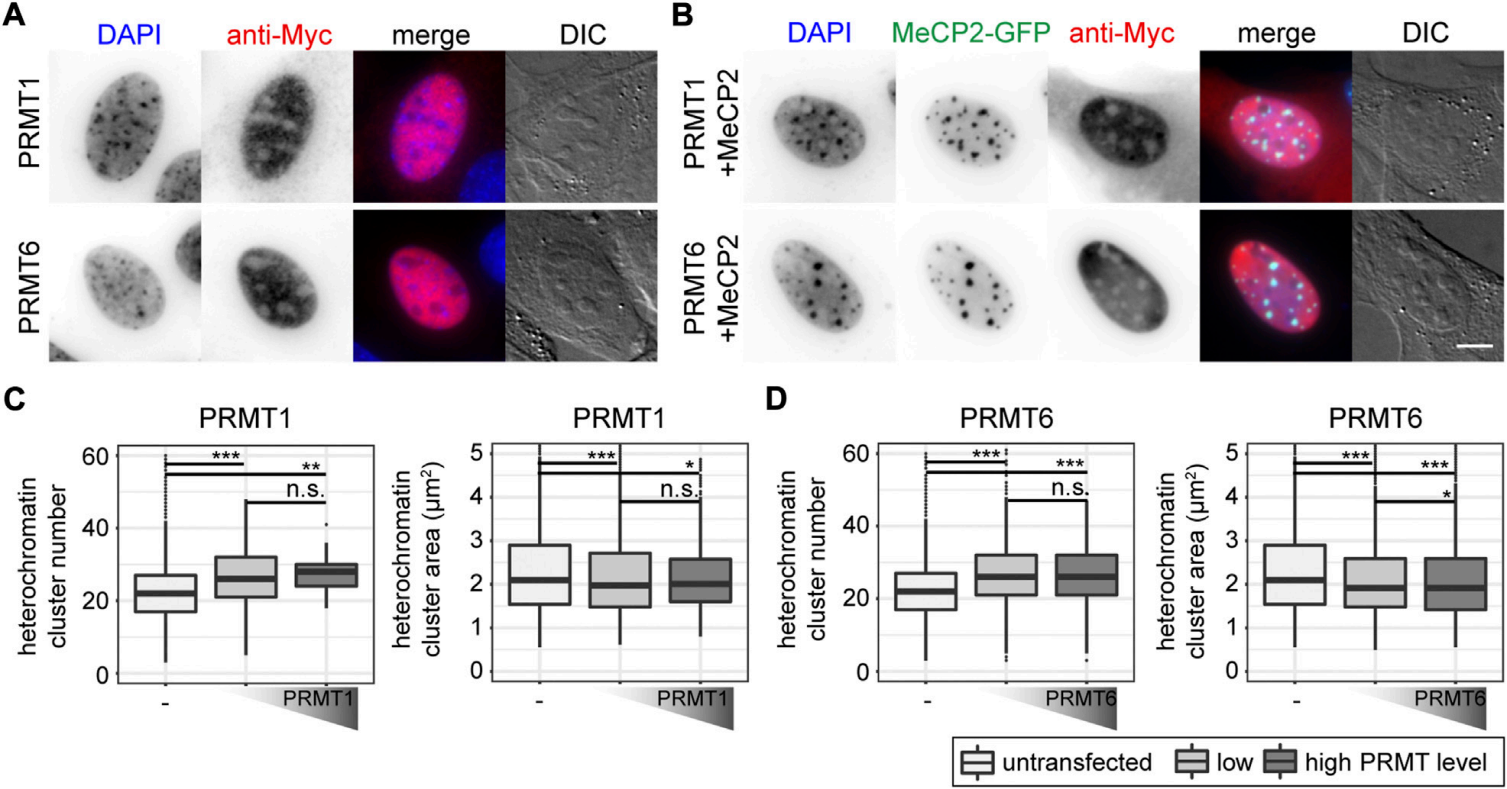
Subcellular localization of PRMT1 and PRMT6 constructs transfected in C2C12 mouse myoblast cells in the absence **(A)** and presence **(B)** of MeCP2. Scale bar 5 µm. Boxplots depict the heterochromatin clustering in C2C12 cells without and with low and high levels of nuclear PRMT1 **(C)** and 6 **(D)** represented by the number and size of the heterochromatin clusters obtained from high-content screening microscopy. Two biological replicates, statistical significance calculated using Wilcoxon-Rank test. *p < 0.05, **p < 0.005, ***p < 0.001, n. s Not significant. *p*-values and n-values are summarized in Supplementary Table S6.

As we observed changes in MeCP2 heterochromatin accumulation, clustering and binding kinetics for the constructs mutated for arginine methylation sites, we tested whether these changes are due to the mutations inserted or a consequence of arginine methylation. Therefore, we performed coexpression experiments of protein arginine methyltransferases (*PRMTs*) with *Mecp2* to test whether the PRMTs affect the MeCP2 heterochromatin clustering function. We confirmed transfected cell viability by cell cycle profiling (Supplementary Figure S10). We made use of recombinant PRMTs with a Myc-tag that could be used for detection. The PRMTs tested comprised three enzymes that catalyze mono- and asymmetric dimethylation on arginines namely PRMT1, 4, 6, as well as PRMT5 that catalyzes mono- and symmetric arginine dimethylation. We chose PRMT1, as it is the most common arginine methyltransferase responsible for about 85% of all arginine methylations (Nicholson et al., 2009). In addition, PRMT1 and PRMT6 preferentially methylate arginines in glycine and arginine rich (GAR) motifs (Bedford, 2007; Nicholson et al., 2009) and two of the sites identified, R91 and R162, are localized adjacent to lysines. PRMT4 and PRMT1 can cooperate in gene regulation (Kleinschmidt et al., 2008), but cannot substitute each other in all contexts (Herrmann et al., 2009). PRMT5 is the predominant type II PRMT catalyzing symmetric arginine methylation and was associated to transcriptional repression (Stopa et al., 2015). It was reported before that the subcellular localization of the PRMT enzymes is highly dependent on the cell type and the target proteins (Herrmann et al., 2009). Thus, to test for their subcellular localization, C2C12 cells were transfected with the PRMTs, fixed and stained using an antibody against the Myc tag. Of the four PRMTs tested, PRMT1 localized in the nucleus and to a lesser extent in the cytoplasm, while PRMT6 localized exclusively in the nucleus (Figures 6A,B), which is in line with previous studies (Frankel et al., 2002; Herrmann et al., 2009). PRMT4 and PRMT5 were localized in the cytoplasm and, thus, not considered in further experiments (Supplementary Figure S12). None of the PRMTs had an influence on MeCP2 localization (Figures 6A,B, Supplementary Figure S12).

First, we tested whether the PRMTs alone have an influence on heterochromatin clustering in C2C12 cells by plotting heterochromatin cluster numbers and areas (Figures 6C,D). The values for heterochromatin cluster numbers and areas were obtained from high-content microscopy data after segmentation of nuclei and heterochromatin clusters (Supplementary Figure S2) and binning of the cells into low and high protein levels based on their nucleus mean fluorescence intensities. The presence of PRMT1 and PRMT6 resulted in higher heterochromatin cluster numbers and smaller heterochromatin cluster areas compared to untransfected cells, meaning they counteract the clustering of heterochromatin compartments. This effect was observed independent of the PRMT level, as there was no difference in heterochromatin cluster numbers and areas between low and high PRMT levels (Figures 6C,D).

**FIGURE 7:**
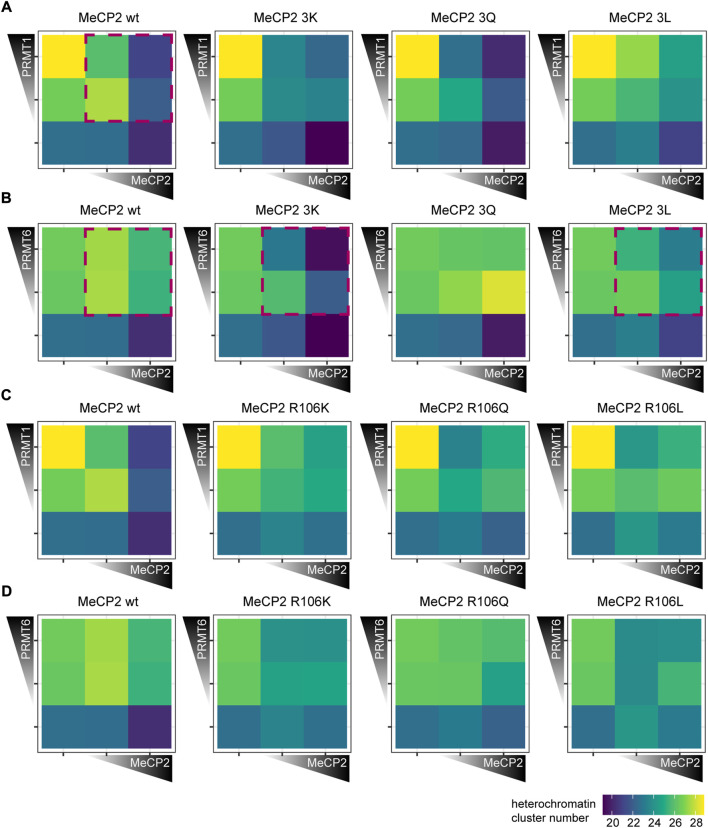
Heterochromatin clustering of cells transfected with MeCP2 mutant constructs in the presence of protein arginine methyltransferases (PRMTs) 1 and 6. Heatmaps show the heterochromatin cluster numbers obtained by high-content screening microscopy of C2C12 cells cotransfected with MeCP2 wild type or triple mutants and PRMT1 **(A)**, wild type or triple mutants and PRMT6 **(B)**, wild type or R106 mutants and PRMT1 **(C)** and wild type or R106 mutants and PRMT6 **(D)**. Cells were binned for low and high fluorescence intensity according to their MeCP2 and PRMT levels. Heterochromatin cluster numbers are shown as means of at least 26 cells from at least two biological replicates. The *p*-values representing the statistical significance calculated using Wilcoxon-Rank test are listed in Supplementary Table S6. Red boxes mark the most important observations.

Next, C2C12 cells were cotransfected with *PRMT1* and *PRMT6* together with *Mecp2* wild type or mutant constructs, cells were fixed and stained for the Myc tag and subsequently imaged on a high-content microscope (Supplementary Figure S2). The heterochromatin cluster numbers (Figure 7) and areas (Supplementary Figure S13) were plotted as heatmaps for each PRMT in combination with the MeCP2 triple mutants.

Introduction of PRMT1 and PRMT6 together with MeCP2 wild type into cells resulted in a significantly decreased MeCP2 heterochromatin clustering shown by higher heterochromatin cluster numbers and smaller cluster areas (Figure 7; Supplementary Figure S13). While MeCP2 at high levels could still cluster heterochromatin in the presence of PRMT1, there was nearly no clustering in the presence of PRMT6.

The cotransfection experiments of MeCP2 and PRMTs revealed differences in heterochromatin clustering between MeCP2 modification site mutants and MeCP2 wild type. Comparing the triple mutations to MeCP2 wild type in presence of PRMT1, MeCP2 3Q showed a similar heterochromatin cluster number distribution as the wild type, while the heatmaps of 3K and 3L differed (Figure 7A). MeCP2 3K and 3L in presence of PRMT1 showed lower heterochromatin numbers in high levels compared to wildtype, but the changes observed were not statistically significant. The presence of PRMT6 blocked the ability of MeCP2 wild type to induce heterochromatin clustering, and the same was observed for MeCP2 3Q (Figure 7B, Supplementary Figure S13B). MeCP2 3K, though, showed lower heterochromatin cluster numbers than wild type MeCP2 when cotransfected with PRMT6 and, thus, shows higher heterochromatin clustering. Thus, MeCP2 3K was able to reverse the negative effect of PRMT6 on heterochromatin clustering and the R to K substitution prevents its methylation by PRMTs. MeCP2 3L at high levels increased heterochromatin clustering (shown by lower heterochromatin cluster numbers and larger areas) in presence of PRMT6, but its clustering function was still impaired compared to its expression without PRMT6 (Figure 7B, Supplementary Figure S13B). Thus, we conclude that the presence of PRMTs influences the heterochromatin clustering function of MeCP2 and its triple mutants. The differences in clustering are specifically pronounced comparing the positively charged lysine mutation with the uncharged but still polar glutamine and the non-polar leucine, emphasizing the importance of the positive charge for the MeCP2 heterochromatin clustering function.

The cotransfection experiments of MeCP2 R106 mutants and PRMT1 and PRMT6 revealed that the mutation of R106 decreases MeCP2 heterochromatin clustering function showing higher cluster numbers and smaller areas (Figures 7C,D, Supplementary Figures S12C,D). The R106 mutants could not counteract the reduced clustering function in the presence of PRMT1 and there was no clear difference in heterochromatin clustering between R106K, Q and L mutants. In the presence of PRMT6 MeCP2 R106Q showed no significant heterochromatin clustering, while the presence of R106K and R106L increased heterochromatin clustering when cotransfected with PRMT6. Thus, the influence of PRMT6 on the R106 mutant heterochromatin clustering shows similar tendencies as for the 3x mutants, but less pronounced.

## 4 Discussion

MeCP2 is post-translationally modified and some of these modifications might influence its transcriptional regulation and protein-protein interactions (Bellini et al., 2014), as well as its chromatin clustering abilities (Becker et al., 2016). Although many modifications have been reported, only few of them were functionally characterized or identified *in vivo*. In this study, we show that MeCP2 isolated from adult mouse brain is post-translationally phosphorylated on serines and threonines, methylated and acetylated on lysines and methylated on arginines. Although MeCP2 is rich in arginines (comprising 7.2% of the amino acids), we identified only a few of them as modified by methylation. One possible explanation is the removal of the modification during the experimental procedure. Although arginine methylation is considered a rather stable and permanent modification, recent reports argue for the existence of arginine demethylation enzymes (Bedford and Clarke, 2009; Wesche et al., 2017). On the one hand, studies involving drug treatments revealed rapid changes in arginine methylation (Le Romancer et al., 2008; Sylvestersen et al., 2014; Tsai et al., 2016). On the other hand, candidate proteins catalyzing active arginine demethylation are discussed, among them several lysine demethylases (Chang et al., 2007; Walport et al., 2016; Wesche et al., 2017). Thus, although we might not have identified all possible arginine methylation sites, we conclude that arginine methylation on MeCP2 occurs mostly on a few specific sites and should be tightly regulated.

**FIGURE 8:**
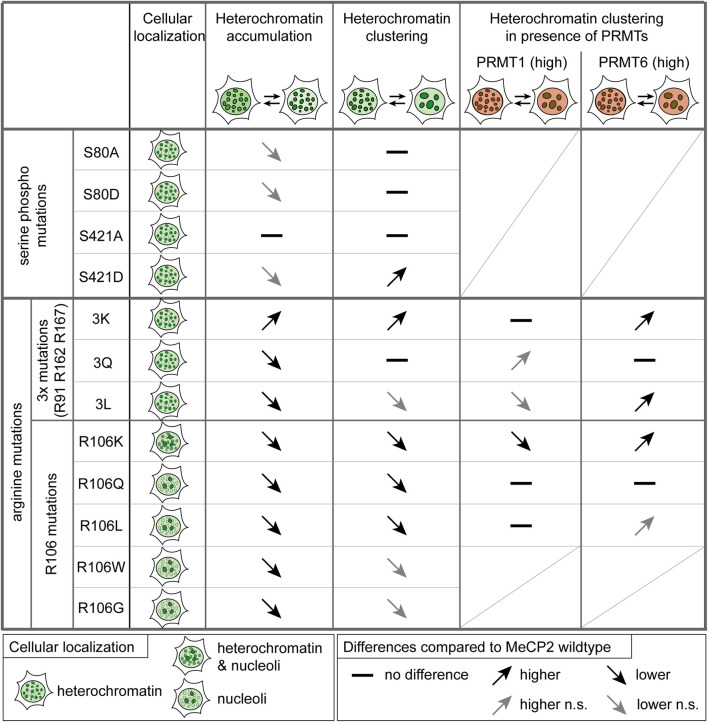
Summary of the functional characterization of MeCP2-induced heterochromatin organization. Functional differences of the MeCP2 PTM mutants in comparison to the wild type protein are represented as arrows showing increase (pointing up) and decrease (pointing down), if not statistically significant (n.s.) in gray, and no difference is marked by a line. Triple mutations stands for R91/R162/R167 mutations. Heterochromatin accumulation and clustering are MeCP2 dose-dependent. For the heterochromatin clustering in presence of PRMT1 and 6, only high PRMT levels were considered.

As MeCP2 serine 80 and 421 phosphorylation sites are well validated but not functionally characterized in the context of heterochromatin organization, we also analyzed their heterochromatin accumulation, clustering properties and binding kinetics (see Figure 8). The phospho-mimicking mutant S80D showed faster heterochromatin binding kinetics than wild type MeCP2, indicating reduced heterochromatin binding. By contrast, Tao et al. (2009) observed a decrease in MeCP2 chromatin binding affinity of the phospho-null MeCP2 S80A mutant to *Pomc* and *Gtl2* promoters by ChIP-qPCR. As with this method, the authors measured MeCP2 binding to selected genomic regions and not the overall MeCP2 heterochromatin binding kinetics, we used different methods to elucidate overall (hetero)chromatin association. Thus, our results contribute to understanding the function of MeCP2 S80 phosphorylation in global heterochromatin binding. MeCP2 S80 plays a role in heterochromatin association, but not in its clustering. In contrast, the serine 421 phosphorylation mimicking mutant S421D showed increased heterochromatin clustering (with lower heterochromatin cluster numbers and larger cluster areas) compared to wild type MeCP2. As S421 phosphorylation was found exclusively in the brain upon neuronal stimulation (Zhou et al., 2006; Tao et al., 2009), we propose that clustering of heterochromatin compartments plays a role in this process. Albeit heterochromatin binding often correlates to the ability of proteins to cluster heterochromatin together over time in the cell nucleus, it is not the only factor. In fact, whereas the binding of the Rett P101H mutant MeCP2 protein is similar to the wild type MeCP2, its clustering ability is totally impaired (Agarwal et al., 2011) and cannot be rescued by artificially targeting it to heterochromatin (Casas-Delucchi et al., 2012).

To observe the consequences of *PRMT1* and *PRMT6*, we introduced plasmids coding for PRMT one and six alone as well as together with MeCP2 in mouse myoblast cells. Solely the expression of *PRMT1* and *PRMT6* reduced the overall heterochromatin cluster size concomitantly increasing their numbers. PRMT1 mostly acts as a coactivator of transcription (An et al., 2004; Nicholson et al., 2009), whereas PRMT6 mostly acts as transcriptional repressor (Guccione et al., 2007; Hyllus et al., 2007; Stein et al., 2012; Stein et al., 2016), but it was also reported to contribute to gene activation (Bouchard et al., 2018). Interestingly, cells from cancer patients showed higher *PRMT1* and *PRMT6* expression than cells from healthy tissue, which seemed to be beneficial for tumor growth (Yoshimatsu et al., 2011). Furthermore, *PRMT6* upregulation was found to correlate with DNA hypomethylation in mESCs and MCF7 cells (Veland et al., 2017), providing a possible explanation for the reduced heterochromatin clustering we observed upon *PRMT6* overexpression. Coexpression of *Mecp2* wild type and *PRMT1* and *PRMT6* lead to significantly decreased MeCP2-mediated heterochromatin clustering (indicated by higher cluster numbers and smaller areas), suggesting that a high degree of arginine methylation in the cells drastically impairs MeCP2 clustering function. This effect was more pronounced for PRMT6 than for PRMT1, although PRMT1 is considered responsible for the majority of cellular arginine methylation (Tang et al., 2000). Thus, PRMT6 might have a higher affinity for MeCP2 than PRMT1. Moreover, *PRMT1* and *PRMT6* expression was reported to depend on the MeCP2 level in a neuroblastoma cell line, thus suggesting a positive gene regulatory interaction between MeCP2 and these two genes (Vecsler et al., 2010).

The overall changes in heterochromatin clustering upon *PRMT* overexpression could be explained either by overall higher arginine methylation levels or by increased arginine methylation levels of MeCP2. To distinguish between these possibilities we made use of the MeCP2 arginine point mutations, which are not modifiable by the PRMTs. For this purpose, we generated MeCP2 constructs mutated for the modification sites identified (R91, R162, and R167) substituting the arginine with lysine (3K), glutamine (3Q) or leucine (3L). Although none of these substitutions can truly mimic the different arginine (modification) states, lysine retains the positive charge as (methylated) arginine, the polar glutamine should sterically mimic a methylated arginine and the unpolar leucine should mimic the methyl groups of methylated arginine (Supplementary Figure S8C, Figure 1E). Furthermore, arginine substitutions by glutamine and leucine were also found in Rett syndrome patients. Arginine substitution with the positively charged lysine increased heterochromatin accumulation and clustering in comparison to wild type MeCP2. In contrast, substitutions with glutamine and leucine reduced heterochromatin accumulation and lead to faster heterochromatin binding kinetics effectively reducing the t ½ to half of the one obtained with wild type MeCP2. These results indicate the importance of the positive charge on R91, R162, and R167 for MeCP2 heterochromatin accumulation and clustering but especially for heterochromatin binding kinetics. Of note, MeCP2 3Q seems to be the best MeCP2 mimic for heterochromatin clustering emphasizing that its polarity and steric properties are more similar to those of wild type MeCP2. The absence of charge and polarity clearly impacts all functional properties of MeCP2 tested here as seen with the MeCP2 3L mutant. Thus, we hypothesize that MeCP2 is methylated in brain at any given time at least at one of the arginine methylation sites identified and that this modification partially changes the positive charge distribution. Thus, methylated arginines might show similar properties as polar amino acids. In fact, it was described that arginine methylation alters the charge distribution to more diffuse (especially in case of dimethylation) but still positive electrostatic properties (Evich et al., 2016; Lorton and Shechter, 2019). In addition, methylation changes arginine shape and reduces the number of possible hydrogen bonds (McBride and Silver, 2001; Bedford and Clarke, 2009). Cotransfection experiments of MeCP2 triple mutants and various PRMTs revealed differences in heterochromatin clustering between the mutants and the wild type protein, further strengthening the evidence for arginine methylation on one or more of the identified modification sites. In the presence of PRMT6, MeCP2 3K and 3L showed significantly higher heterochromatin clustering function represented by lower heterochromatin cluster numbers and larger areas than wild type MeCP2. MeCP2 3Q hardly induced any clustering, similar to MeCP2 wild type. MeCP2 3K showed higher clustering abilities (lower heterochromatin numbers and larger areas) than wild type MeCP2 and was able to reverse the effect of PRMT6 alone, which induced reduced clustering of heterochromatin. This effect might not be a direct result of the positive charge on heterochromatin clustering, but rather an indirect one as MeCP2 3K cannot be methylated by PRMTs on the mutated arginines. In comparison to the wild type protein, the MeCP2 3L mutant showed higher clustering when PRMT6 was introduced, although it showed decreased clustering without PRMT6. These results suggest a very high arginine methylation level of wild type MeCP2 in the coexpression experiment, which decreases its clustering ability to such an extent that even the MeCP2 3L mutant clusters more than the methylated wild type protein. From the increased heterochromatin clustering functions of the 3K and 3L mutant, which cannot be methylated on the substituted arginines, we conclude that MeCP2 gets methylated by PRMT6 on these sites. Of note, arginine methylation catalyzed by PRMT1 and PRMT6 often takes place on arginines flanked by one or more glycines in so called glycine and arginine rich (GAR) motifs (Lorton and Shechter, 2019) and MeCP2 R91 and R162 are localized adjacent to glycines. Although our results emphasize that MeCP2 gets methylated on arginines and in consequence shows reduced heterochromatin clustering abilities, we cannot exclude that consequences of high arginine methylation levels also indirectly impact MeCP2 heterochromatin clustering. Examples could be the modification of MeCP2 interacting proteins or other proteins involved in heterochromatin clustering, e.g., histones. Interestingly, MBD2, another member of the methyl-CpG binding protein family, was shown to undergo arginine methylation, which resulted in reduced DNA binding and reduced functionality in transcriptional repression (Tan and Nakielny, 2006).

MeCP2 R106 was identified as dimethylated in our mass spectrometry analysis and is commonly mutated in Rett syndrome patients to tryptophan (W) and glutamine (Q), in very few cases also to glycine (G) and leucine (L). MeCP2 R106W is a frequent Rett syndrome mutation causing severe phenotypes (Cuddapah et al., 2014) and the less common R106Q mutation was described to cause “classic” Rett syndrome (Bienvenu et al., 2000; Fukuda et al., 2005; Zahorakova et al., 2016), but there is insufficient clinical information reported for individuals with R106Q, R106G and R106L for a comparison of phenotypes (see RettBASE, (Krishnaraj et al., 2017)). R106 W was reported to abolish DNA binding, while R106Q reduced it (Ballestar et al., 2000; Yang et al., 2016). Accordingly, our experiments showed that all R106 mutants mainly lost heterochromatin accumulation and mislocalized to the negatively charged nucleoli due to the high amount of positively charged amino acids in MeCP2 (Martin et al., 2015). The reduced DNA binding and accumulation can be explained by the location of R106 close to the Asx-ST motif, which stabilizes MeCP2 DNA binding ((Ho et al., 2008), Figure 2E). H/DX experiments revealed similar dynamic protein behavior of MeCP2 R106W and wild type protein (Hansen et al., 2011) and circular dichroism spectra of R106W/Q showed no major changes in secondary structure compared to wild type MeCP2 (Ballestar et al., 2000; Yang et al., 2016). Instead, molecular modeling of the MeCP2 R106W/Q structures pointed towards local changes of hydrogen bonds and salt bridges (Kucukkal et al., 2015; Pedretti et al., 2016; Yang et al., 2016), which might cause changes in DNA binding as R106 is part of a ß-strand in the MBD structure, stabilizes the Asx-ST motif and is buried and not exposed to the surrounding (Kucukkal et al., 2015; Pedretti et al., 2016). Thus, MeCP2 R106W/Q mutations have not been found to induce changes in overall MeCP2 structure but rather result in smaller local changes in amino acid interactions. The heterochromatin clustering was highly impaired in the MeCP2 R106 mutants as well. The reason might be the lack of binding to pericentric heterochromatin regions as the clustering abilities of some Rett mutants could be rescued by repositioning of the proteins to the heterochromatin regions (Casas-Delucchi et al., 2012). In addition, we recently showed that heterochromatin clustering *in vivo* can be modeled by *in vitro* phase separation (Zhang et al., 2022). The minimal basis for MeCP2 liquid-liquid phase separation was electrostatic self-interaction, but also DNA promoted *de novo* phase separation of MeCP2 in physiological salt conditions (Zhang et al., 2022). Thus, DNA binding as well as oligomerization *via* its ID and TRD domain (Becker et al., 2013) is involved in MeCP2 heterochromatin clustering. From the cotransfection experiments of PRMTs and MeCP2 R106 mutants it could be hypothesized that R106 is more likely to be methylated by PRMT6 than by PRMT1, as PRMT6 presence affected the clustering by MeCP2 R106 mutants. Altogether, our results demonstrate that post-translational modifications of MeCP2, in particular arginine methylation and to a lesser extent serine phosphorylation, play an essential role in modulating MeCP2 function in heterochromatin organization.

## Data Availability

The datasets presented in this study can be found in online repositories. The names of the repositories and accession numbers can be found below: The data was uploaded to TUDataLib accessible with the link https://doi.org/10.48328/tudatalib-868. The mass spectrometry proteomics data have been deposited to the ProteomeXchange Consortium via the PRIDE (Perez Riverol et al., 2019) partner repository with the dataset identifier PXD033696.

## References

[B1] Adams V. H. McBryant S. J. Wade P. A. Woodcock C. L. Hansen J. C. (2007). Intrinsic disorder and autonomous domain function in the multifunctional nuclear protein, MeCP2. J. Biol. Chem. 282, 15057–15064. 10.1074/jbc.M700855200 17371874

[B2] Agarwal N. Becker A. Jost K. L. Haase S. Thakur B. K. Brero A. (2011). MeCP2 Rett mutations affect large scale chromatin organization. Hum. Mol. Genet. 20, 4187–4195. 10.1093/hmg/ddr346 21831886

[B3] Agarwal N. Hardt T. Brero A. Nowak D. Rothbauer U. Becker A. (2007). MeCP2 interacts with HP1 and modulates its heterochromatin association during myogenic differentiation. Nucleic Acids Res. 35, 5402–5408. 10.1093/nar/gkm599 17698499 PMC2018631

[B4] Amir R. E. Van den Veyver I. B. Wan M. Tran C. Q. Francke U. Zoghbi H. Y. (1999). Rett syndrome is caused by mutations in X-linked MECP2, encoding methyl-CpG-binding protein 2. Nat. Genet. 23, 185–188. 10.1038/13810 10508514

[B5] An W. Kim J. Roeder R. G. (2004). Ordered cooperative functions of PRMT1, p300, and CARM1 in transcriptional activation by p53. Cell 117, 735–748. 10.1016/j.cell.2004.05.009 15186775

[B6] Baccarini P. (1908). Sulle cinesi vegetative del *“Cynomorium coccineum L* . N. Giorn. Bot. Ital. N. Ser. 15, 189–203.

[B7] Ballestar E. Yusufzai T. M. Wolffe A. P. (2000). Effects of Rett syndrome mutations of the methyl-CpG binding domain of the transcriptional repressor MeCP2 on selectivity for association with methylated DNA. Biochemistry 39, 7100–7106. 10.1021/bi0001271 10852707

[B8] Becker A. Allmann L. Hofstätter M. Casà V. Weber P. Lehmkuhl A. (2013). Direct homo- and hetero-interactions of MeCP2 and MBD2. PLoS ONE 8, e53730. 10.1371/journal.pone.0053730 23335972 PMC3546041

[B9] Becker A. Zhang P. Allmann L. Meilinger D. Bertulat B. Eck D. (2016). Poly(ADP-ribosyl)ation of methyl CpG binding domain protein 2 regulates chromatin structure. J. Biol. Chem. 291, 4873–4881. 10.1074/jbc.M115.698357 26772194 PMC4777825

[B10] Bedford M. T. (2007). Arginine methylation at a glance. J. Cell Sci. 120, 4243–4246. 10.1242/jcs.019885 18057026

[B11] Bedford M. T. Clarke S. G. (2009). Protein arginine methylation in mammals: Who, what, and why. Mol. Cell 33, 1–13. 10.1016/j.molcel.2008.12.013 19150423 PMC3372459

[B12] Bellini E. Pavesi G. Barbiero I. Bergo A. Chandola C. Nawaz M. S. (2014). MeCP2 post-translational modifications: A mechanism to control its involvement in synaptic plasticity and homeostasis? Front. Cell. Neurosci. 8, 236. 10.3389/fncel.2014.00236 25165434 PMC4131190

[B13] Bertulat B. De Bonis M. L. Della Ragione F. Lehmkuhl A. Milden M. Storm C. (2012). MeCP2 dependent heterochromatin reorganization during neural differentiation of a novel Mecp2-deficient embryonic stem cell reporter line. PLoS ONE 7, e47848. 10.1371/journal.pone.0047848 23112857 PMC3480415

[B14] Bienvenu T. Carrié A. de Roux N. Vinet M. C. Jonveaux P. Couvert P. (2000). MECP2 mutations account for most cases of typical forms of Rett syndrome. Hum. Mol. Genet. 9, 1377–1384. 10.1093/hmg/9.9.1377 10814719

[B15] Bouchard C. Sahu P. Meixner M. Nötzold R. R. Rust M. B. Kremmer E. (2018). Genomic location of PRMT6-dependent H3R2 methylation is linked to the transcriptional outcome of associated genes. Cell Rep. 24, 3339–3352. 10.1016/j.celrep.2018.08.052 30232013

[B16] Brero A. Easwaran H. P. Nowak D. Grunewald I. Cremer T. Leonhardt H. (2005). Methyl CpG-binding proteins induce large-scale chromatin reorganization during terminal differentiation. J. Cell Biol. 169, 733–743. 10.1083/jcb.200502062 15939760 PMC2171616

[B17] Casas-Delucchi C. S. Becker A. Bolius J. J. Cardoso M. C. (2012). Targeted manipulation of heterochromatin rescues MeCP2 Rett mutants and re-establishes higher order chromatin organization. Nucleic Acids Res. 40, e176. 10.1093/nar/gks784 22923521 PMC3526307

[B18] Cerletti M. Paggi R. A. Guevara C. R. Poetsch A. De Castro R. E. (2015). Global role of the membrane protease LonB in Archaea: Potential protease targets revealed by quantitative proteome analysis of a lonB mutant in Haloferax volcanii. J. Proteomics 121, 1–14. 10.1016/j.jprot.2015.03.016 25829260

[B19] Chahrour M. Jung S. Y. Shaw C. Zhou X. Wong S. T. C. Qin J. (2008). MeCP2, a key contributor to neurological disease, activates and represses transcription. Science 320, 1224–1229. 10.1126/science.1153252 18511691 PMC2443785

[B20] Chang B. Chen Y. Zhao Y. Bruick R. K. (2007). JMJD6 is a histone arginine demethylase. Science 318, 444–447. 10.1126/science.1145801 17947579

[B21] Chen D. Ma H. Hong H. Koh S. S. Huang S. M. Schurter B. T. (1999). Regulation of transcription by a protein methyltransferase. Science 284, 2174–2177. 10.1126/science.284.5423.2174 10381882

[B22] Cuddapah V. A. Pillai R. B. Shekar K. V. Lane J. B. Motil K. J. Skinner S. A. (2014). Methyl-CpG-binding protein 2(MECP2) mutation type is associated with disease severity in Rett syndrome. J. Med. Genet. 51, 152–158. 10.1136/jmedgenet-2013-102113 24399845 PMC4403764

[B23] Dyballa N. Metzger S. (2009). Fast and sensitive colloidal coomassie G-250 staining for proteins in polyacrylamide gels. JoVE 1, 1431. 10.3791/1431 PMC3149902 19684561

[B24] Eng J. K. McCormack A. L. Yates J. R. (1994). An approach to correlate tandem mass spectral data of peptides with amino acid sequences in a protein database. J. Am. Soc. Mass Spectrom. 5, 976–989. 10.1016/1044-0305(94)80016-2 24226387

[B25] Evich M. Stroeva E. Zheng Y. G. Germann M. W. (2016). Effect of methylation on the side-chain pKavalue of arginine. Protein Sci. 25, 479–486. 10.1002/pro.2838 26540340 PMC4815340

[B26] Falk M. Feodorova Y. Naumova N. Imakaev M. Lajoie B. R. Leonhardt H. (2019). Heterochromatin drives compartmentalization of inverted and conventional nuclei. Nature 570, 395–399. 10.1038/s41586-019-1275-3 31168090 PMC7206897

[B27] Fan C. Zhang H. Fu L. Li Y. Du Y. Qiu Z. (2020). Rett mutations attenuate phase separation of MeCP2. Cell Discov. 6, 38. 10.1038/s41421-020-0172-0 32566246 PMC7296026

[B28] Frankel A. Yadav N. Lee J. Branscombe T. L. Clarke S. Bedford M. T. (2002). The novel human protein arginine N-methyltransferase PRMT6 is a nuclear enzyme displaying unique substrate specificity. J. Biol. Chem. 277, 3537–3543. 10.1074/jbc.M108786200 11724789

[B29] Fukuda T. Yamashita Y. Nagamitsu S. Miyamoto K. Jin J.-J. Ohmori I. (2005). Methyl-CpG binding protein 2 gene (MECP2) variations in Japanese patients with rett syndrome: Pathological mutations and polymorphisms. Brain Dev. 27, 211–217. 10.1016/j.braindev.2004.06.003 15737703

[B30] Georgel P. T. Horowitz-Scherer R. A. Adkins N. Woodcock C. L. Wade P. A. Hansen J. C. (2003). Chromatin compaction by human MeCP2. J. Biol. Chem. 278, 32181–32188. 10.1074/jbc.M305308200 12788925

[B31] Goulet I. Gauvin G. Boisvenue S. Côté J. (2007). Alternative splicing yields protein arginine methyltransferase 1 isoforms with distinct activity, substrate specificity, and subcellular localization. J. Biol. Chem. 282, 33009–33021. 10.1074/jbc.M704349200 17848568

[B32] Guccione E. Bassi C. Casadio F. Martinato F. Cesaroni M. Schuchlautz H. (2007). Methylation of histone H3R2 by PRMT6 and H3K4 by an MLL complex are mutually exclusive. Nature 449, 933–937. 10.1038/nature06166 17898714

[B33] Hansen J. C. Wexler B. B. Rogers D. J. Hite K. C. Panchenko T. Ajith S. (2011). DNA binding restricts the intrinsic conformational flexibility of methyl CpG binding protein 2 (MeCP2). J. Biol. Chem. 286, 18938–18948. 10.1074/jbc.M111.234609 21467044 PMC3099709

[B34] Heckman K. L. Pease L. R. (2007). Gene splicing and mutagenesis by PCR-driven overlap extension. Nat. Protoc. 2, 924–932. 10.1038/nprot.2007.132 17446874

[B35] Heinz K. S. Casas-Delucchi C. S. Török T. Cmarko D. Rapp A. Raska I. (2018). Peripheral re-localization of constitutive heterochromatin advances its replication timing and impairs maintenance of silencing marks. Nucleic Acids Res. 46, 6112–6128. 10.1093/nar/gky368 29750270 PMC6158597

[B36] Herrmann F. Pably P. Eckerich C. Bedford M. T. Fackelmayer F. O. (2009). Human protein arginine methyltransferases *in vivo* - distinct properties of eight canonical members of the PRMT family. J. Cell Sci. 122, 667–677. 10.1242/jcs.039933 19208762

[B37] Ho K. L. McNae I. W. Schmiedeberg L. Klose R. J. Bird A. P. Walkinshaw M. D. (2008). MeCP2 binding to DNA depends upon hydration at methyl-CpG. Mol. Cell 29, 525–531. 10.1016/j.molcel.2007.12.028 18313390

[B38] Hyllus D. Stein C. Schnabel K. Schiltz E. Imhof A. Dou Y. (2007). PRMT6-mediated methylation of R2 in histone H3 antagonizes H3 K4 trimethylation. Genes Dev. 21, 3369–3380. 10.1101/gad.447007 18079182 PMC2113036

[B39] Inuzuka L. M. Guerra-Peixe M. Macedo-Souza L. I. Pedreira C. C. Gurgel-Giannetti J. Monteiro F. P. (2021). MECP2-related conditions in males: A systematic literature review and 8 additional cases. Eur. J. Paediatr. Neurology 34, 7–13. 10.1016/j.ejpn.2021.05.013 34271245

[B40] Jones P. L. Veenstra G. J. Wade P. A. Vermaak D. Kass S. U. Landsberger N. (1998). Methylated DNA and MeCP2 recruit histone deacetylase to repress transcription. Nat. Genet. 19, 187–191. 10.1038/561 9620779

[B41] Jost K. L. Bertulat B. Cardoso M. C. (2012). Heterochromatin and gene positioning: Inside, outside, any side? Chromosoma 121, 555–563. 10.1007/s00412-012-0389-2 23090282 PMC3501169

[B42] Jost K. L. Rottach A. Milden M. Bertulat B. Becker A. Wolf P. (2011). Generation and characterization of rat and mouse monoclonal antibodies specific for MeCP2 and their use in X-inactivation studies. PLoS ONE 6, e26499. 10.1371/journal.pone.0026499 22140431 PMC3225355

[B43] Kleinschmidt M. A. Streubel G. Samans B. Krause M. Bauer U.-M. (2008). The protein arginine methyltransferases CARM1 and PRMT1 cooperate in gene regulation. Nucleic Acids Res. 36, 3202–3213. 10.1093/nar/gkn166 18413343 PMC2425501

[B44] Kokura K. Kaul S. C. Wadhwa R. Nomura T. Khan M. M. Shinagawa T. (2001). The Ski protein family is required for MeCP2-mediated transcriptional repression. J. Biol. Chem. 276, 34115–34121. 10.1074/jbc.M105747200 11441023

[B45] Krishnaraj R. Ho G. Christodoulou J. (2017). RettBASE: Rett syndrome database update. Hum. Mutat. 38, 922–931. 10.1002/humu.23263 28544139

[B46] Kucukkal T. G. Yang Y. Uvarov O. Cao W. Alexov E. (2015). Impact of rett syndrome mutations on mecp2 MBD stability. Biochemistry 54, 6357–6368. 10.1021/acs.biochem.5b00790 26418480 PMC9871983

[B47] Larson A. G. Elnatan D. Keenen M. M. Trnka M. J. Johnston J. B. Burlingame A. L. (2017). Liquid droplet formation by HP1α suggests a role for phase separation in heterochromatin. Nature 547, 236–240. 10.1038/nature22822 28636604 PMC5606208

[B48] Le Romancer M. Treilleux I. Leconte N. Robin-Lespinasse Y. Sentis S. Bouchekioua-Bouzaghou K. (2008). Regulation of estrogen rapid signaling through arginine methylation by PRMT1. Mol. Cell 31, 212–221. 10.1016/j.molcel.2008.05.025 18657504

[B49] Lewis J. D. Meehan R. R. Henzel W. J. Maurer-Fogy I. Jeppesen P. Klein F. (1992). Purification, sequence, and cellular localization of a novel chromosomal protein that binds to methylated DNA. Cell 69, 905–914. 10.1016/0092-8674(92)90610-o 1606614

[B50] Lorton B. M. Shechter D. (2019). Cellular consequences of arginine methylation. Cell. Mol. Life Sci. 76, 2933–2956. 10.1007/s00018-019-03140-2 31101937 PMC6642692

[B51] Lunyak V. V. Burgess R. Prefontaine G. G. Nelson C. Sze S.-H. Chenoweth J. (2002). Corepressor-dependent silencing of chromosomal regions encoding neuronal genes. Science 298, 1747–1752. 10.1126/science.1076469 12399542

[B52] Lyst M. J. Ekiert R. Ebert D. H. Merusi C. Nowak J. Selfridge J. (2013). Rett syndrome mutations abolish the interaction of MeCP2 with the NCoR/SMRT co-repressor. Nat. Neurosci. 16, 898–902. 10.1038/nn.3434 23770565 PMC3786392

[B53] Martin R. M. Ter-Avetisyan G. Herce H. D. Ludwig A. K. Lättig-Tünnemann G. Cardoso M. C. (2015). Principles of protein targeting to the nucleolus. Nucleus 6, 314–325. 10.1080/19491034.2015.1079680 26280391 PMC4615656

[B54] McBride A. E. Silver P. A. (2001). State of the arg. Cell 106, 5–8. 10.1016/s0092-8674(01)00423-8 11461695

[B55] Nan X. Campoy F. J. Bird A. (1997). MeCP2 is a transcriptional repressor with abundant binding sites in genomic chromatin. Cell 88, 471–481. 10.1016/s0092-8674(00)81887-5 9038338

[B56] Nan X. Ng H. H. Johnson C. A. Laherty C. D. Turner B. M. Eisenman R. N. (1998). Transcriptional repression by the methyl-CpG-binding protein MeCP2 involves a histone deacetylase complex. Nature 393, 386–389. 10.1038/30764 9620804

[B57] Nicholson T. B. Chen T. Richard S. (2009). The physiological and pathophysiological role of PRMT1-mediated protein arginine methylation. Pharmacol. Res. 60, 466–474. 10.1016/j.phrs.2009.07.006 19643181

[B58] Pedretti A. Granito C. Mazzolari A. Vistoli G. (2016). Structural effects of some relevant missense mutations on the MECP2-DNA binding: A md study analyzed by Rescore+, a versatile rescoring tool of the vega zz program. Mol. Inf. 35, 424–433. 10.1002/minf.201501030 27546046

[B59] Perez-Riverol Y. Csordas A. Bai J. Bernal-Llinares M. Hewapathirana S. Kundu D. J. (2019). The PRIDE database and related tools and resources in 2019: Improving support for quantification data. Nucleic Acids Res. 47, D442–D450. 10.1093/nar/gky1106 30395289 PMC6323896

[B60] Qian K. Huang C. T.-L. Chen H. Blackbourn L. W. Chen Y. Cao J. (2014). A simple and efficient system for regulating gene expression in human pluripotent stem cells and derivatives. Stem Cells 32, 1230–1238. 10.1002/stem.1653 24497442 PMC4121394

[B61] Ran F. A. Hsu P. D. Wright J. Agarwala V. Scott D. A. Zhang F. (2013). Genome engineering using the CRISPR-Cas9 system. Nat. Protoc. 8, 2281–2308. 10.1038/nprot.2013.143 24157548 PMC3969860

[B62] Rival-Gervier S. Lo M. Y. Khattak S. Pasceri P. Lorincz M. C. Ellis J. (2013). Kinetics and epigenetics of retroviral silencing in mouse embryonic stem cells defined by deletion of the D4Z4 element. Mol. Ther. 21, 1536–1550. 10.1038/mt.2013.131 23752310 PMC3734652

[B63] Schmidt A. Zhang H. Cardoso M. C. (2020). MeCP2 and chromatin compartmentalization. Cells 9, 878. 10.3390/cells9040878 PMC7226738 32260176

[B64] Stein C. Nötzold R. R. Riedl S. Bouchard C. Bauer U.-M. (2016). The arginine methyltransferase PRMT6 cooperates with polycomb proteins in regulating HOXA gene expression. PLoS ONE 11, e0148892. 10.1371/journal.pone.0148892 26848759 PMC4746130

[B65] Stein C. Riedl S. Ruthnick D. Notzold R. R. Bauer U.-M. (2012). The arginine methyltransferase PRMT6 regulates cell proliferation and senescence through transcriptional repression of tumor suppressor genes. Nucleic Acids Res. 40, 9522–9533. 10.1093/nar/gks767 22904088 PMC3479209

[B66] Stopa N. Krebs J. E. Shechter D. (2015). The PRMT5 arginine methyltransferase: Many roles in development, cancer and beyond. Cell. Mol. Life Sci. 72, 2041–2059. 10.1007/s00018-015-1847-9 25662273 PMC4430368

[B67] Strom A. R. Emelyanov A. V. Mir M. Fyodorov D. V. Darzacq X. Karpen G. H. (2017). Phase separation drives heterochromatin domain formation. Nature 547, 241–245. 10.1038/nature22989 28636597 PMC6022742

[B68] Sylvestersen K. B. Horn H. Jungmichel S. Jensen L. J. Nielsen M. L. (2014). Proteomic analysis of arginine methylation sites in human cells reveals dynamic regulation during transcriptional arrest. Mol. Cell. Proteomics 13, 2072–2088. 10.1074/mcp.O113.032748 24563534 PMC4125738

[B69] Tan C. P. Nakielny S. (2006). Control of the DNA methylation system component MBD2 by protein arginine methylation. Mol. Cell. Biol. 26, 7224–7235. 10.1128/MCB.00473-06 16980624 PMC1592890

[B70] Tang J. Frankel A. Cook R. J. Kim S. Paik W. K. Williams K. R. (2000). PRMT1 is the predominant type I protein arginine methyltransferase in mammalian cells. J. Biol. Chem. 275, 7723–7730. 10.1074/jbc.275.11.7723 10713084

[B71] Tao J. Hu K. Chang Q. Wu H. Sherman N. E. Martinowich K. (2009). Phosphorylation of MeCP2 at Serine 80 regulates its chromatin association and neurological function. Proc. Natl. Acad. Sci. U.S.A. 106, 4882–4887. 10.1073/pnas.0811648106 19225110 PMC2660725

[B72] Tillotson R. Selfridge J. Koerner M. V. Gadalla K. K. E. Guy J. De Sousa D. (2017). Radically truncated MeCP2 rescues Rett syndrome-like neurological defects. Nature 550, 398–401. 10.1038/nature24058 29019980 PMC5884422

[B73] Tsai W.-C. Gayatri S. Reineke L. C. Sbardella G. Bedford M. T. Lloyd R. E. (2016). Arginine demethylation of G3BP1 promotes stress granule assembly. J. Biol. Chem. 291, 22671–22685. 10.1074/jbc.M116.739573 27601476 PMC5077203

[B74] Tyanova S. Temu T. Cox J. (2016). The MaxQuant computational platform for mass spectrometry-based shotgun proteomics. Nat. Protoc. 11, 2301–2319. 10.1038/nprot.2016.136 27809316

[B75] UniProt Consortium (2021). UniProt: The universal protein knowledgebase in 2021. Nucleic Acids Res. 49, D480–D489. 10.1093/nar/gkaa1100 33237286 PMC7778908

[B76] Vecsler M. Simon A. J. Amariglio N. Rechavi G. Gak E. (2010). MeCP2 deficiency down-regulates specific nuclear proteins that could be partially recovered by valproic acid *in vitro* . Epigenetics 5, 61–67. 10.4161/epi.5.1.10630 20093853

[B77] Veland N. Hardikar S. Zhong Y. Gayatri S. Dan J. Strahl B. D. (2017). The arginine methyltransferase PRMT6 regulates DNA methylation and contributes to global DNA hypomethylation in cancer. Cell Rep. 21, 3390–3397. 10.1016/j.celrep.2017.11.082 29262320 PMC5753604

[B78] Walport L. J. Hopkinson R. J. Chowdhury R. Schiller R. Ge W. Kawamura A. (2016). Arginine demethylation is catalysed by a subset of JmjC histone lysine demethylases. Nat. Commun. 7, 11974. 10.1038/ncomms11974 27337104 PMC4931022

[B79] Wang L. Hu M. Zuo M.-Q. Zhao J. Wu D. Huang L. (2020). Rett syndrome-causing mutations compromise MeCP2-mediated liquid-liquid phase separation of chromatin. Cell Res. 30, 393–407. 10.1038/s41422-020-0288-7 32111972 PMC7196128

[B80] Wesche J. Kühn S. Kessler B. M. Salton M. Wolf A. (2017). Protein arginine methylation: A prominent modification and its demethylation. Cell. Mol. Life Sci. 74, 3305–3315. 10.1007/s00018-017-2515-z 28364192 PMC11107486

[B81] Yang Y. Kucukkal T. G. Li J. Alexov E. Cao W. (2016). Binding analysis of methyl-CpG binding domain of MeCP2 and rett syndrome mutations. ACS Chem. Biol. 11, 2706–2715. 10.1021/acschembio.6b00450 27356039 PMC9860374

[B82] Yoshimatsu M. Toyokawa G. Hayami S. Unoki M. Tsunoda T. Field H. I. (2011). Dysregulation of PRMT1 and PRMT6, Type I arginine methyltransferases, is involved in various types of human cancers. Int. J. Cancer 128, 562–573. 10.1002/ijc.25366 20473859

[B83] Zahorakova D. Lelkova P. Gregor V. Magner M. Zeman J. Martasek P. (2016). MECP2 mutations in Czech patients with rett syndrome and rett-like phenotypes: Novel mutations, genotype-phenotype correlations and validation of high-resolution melting analysis for mutation scanning. J. Hum. Genet. 61, 617–625. 10.1038/jhg.2016.19 26984561

[B84] Zhang H. Romero H. Schmidt A. Gagova K. Qin W. Bertulat B. (2022). MeCP2-induced heterochromatin organization is driven by oligomerization-based liquid-liquid phase separation and restricted by DNA methylation. Nucleus 13, 1–34. 10.1080/19491034.2021.2024691 35156529 PMC8855868

[B85] Zhou Z. Hong E. J. Cohen S. Zhao W.-N. Ho H.-Y. H. Schmidt L. (2006). Brain-specific phosphorylation of MeCP2 regulates activity-dependent Bdnf transcription, dendritic growth, and spine maturation. Neuron 52, 255–269. 10.1016/j.neuron.2006.09.037 17046689 PMC3962021

