# Supplementary material for: MeCP2 heterochromatin organization is modulated by arginine methylation and serine phosphorylation
Source: Front Cell Dev Biol. 2022 Sep 12;10:941493. doi: 10.3389/fcell.2022.941493 (PMC9510713; doi:10.3389/fcell.2022.941493)
Supplement: Supplementary file 1 [file DataSheet1.pdf]

Supplementary Material

1 Supplementary Tables

Supplementary Table 1: Plasmid characteristics.

Name	pc number*	Fluorescent protein	(Gene) species	Promoter	Reference
pEGFP-N1_MeCP2(WT)	-	EGFP	Mus musculus	CMV	Tillotson et al., 2017
AAVS1-TRE3G-EGFP	-	EGFP	synthetic	CAG TRE3G	Qian et al., 2014
HSC1-HS4-GiP	-	EGFP	Gallus gallus (Core HS4 insulator sequence (dimer))	EF-1α	Rival-Gervier et al., 2013
pSpCas9(BB)-2A-Puro (PX459) V2.0	-	-	Streptococcus pyogenes	U6 CAG	Ran et al., 2013
pmMeCP2G wt	4701	EGFP	Mus musculus synthetic	CAG TRE3G	This study
pmMeCP2G R91K R162K R167K (3K)	4702	EGFP	Mus musculus synthetic	CAG TRE3G	This study
pmMeCP2G R91Q R162Q R167Q (3Q)	4703	EGFP	Mus musculus synthetic	CAG TRE3G	This study
pmMeCP2G R91L R162L R167L (3L)	4704	EGFP	Mus musculus synthetic	CAG TRE3G	This study
pmMeCP2G S80A	4705	EGFP	Mus musculus synthetic	CAG TRE3G	This study
pmMeCP2G S80D	4706	EGFP	Mus musculus synthetic	CAG TRE3G	This study
pmMeCP2G S421A	4707	EGFP	Mus musculus synthetic	CAG TRE3G	This study
pmMeCP2G S421D	4708	EGFP	Mus musculus synthetic	CAG TRE3G	This study
pmMeCP2G R106K	4718	EGFP	Mus musculus synthetic	CAG TRE3G	This study
pmMeCP2G R106Q	4719	EGFP	Mus musculus synthetic	CAG TRE3G	This study
pmMeCP2G R106L	4724	EGFP	Mus musculus synthetic	CAG TRE3G	This study
pmMeCP2G R106W	4721	EGFP	Mus musculus synthetic	CAG TRE3G	This study
pmMeCP2G R106G	4722	EGFP	Mus musculus synthetic	CAG TRE3G	This study
phPRMT1-pcDNA3.1**	4795	-	Homo sapiens	CMV	This study
pmPRMT4-pcDNA3.1**	4796	-	Mus musculus	CMV	This study
phPRMT5-pcDNA3.1**	4797	-	Homo sapiens	CMV	This study
phPRMT6-pcDNA3.1***	4798	-	Homo sapiens	CMV	Stein et al., 2012
pTYB1-Mecp2wt	1294	-	Homo sapiens	T7	Georgel et al., 2003

*pc number: plasmid collection number. **plasmid naming according to Cardoso lab plasmid collection. ***originally published as pcDNA3.1 hPRMT6.

Supplementary Table 2: Oligonucleotide characteristics.

Name	Sequence [5' - 3']	Application	Reference
AmpR-Ori HpaI fwd	TTTGTTAACCGCGGAACCCCTATTTG	PCR	this study
AmpR-Ori MluI rev	TTTACGCGTTTTCCATAGGCTC	PCR	this study
GSG-T2A AgeI XmaI fwd	TTTTACCCGGGACCGGTAGTGGAGAGGGCA	PCR	this study
bGHpolyA HpaI rev	CCGTTAACTCCCCAGCATGCCTGCT	PCR	this study
Mecp2 SalI NheI fwd	CCCGCTAGCTTTGTCGACATGGTAGCTGGGATGTTAG GGCTCAGGGAGGAAAAGTCAG	PCR	this study
Mecp2 Linker BamHI rev	GGTGGATCCCGGGCCCGCGGTACCGTGGACGGTATC GATAAGCTTGATATCGAATTCCTGCTAACTCTCTCGG TCAC	PCR	this study
HS4 insulator SpeI fwd	GCCCCCACTAGTCTCTTTTATTGAGCTCACG	PCR	this study
HS4 insulator PspXI rev	TTTTTACTCGAGGCGCGCCTGTCATTCTAAATC	PCR	this study
HS4 MluI EcoRI fwd	GGGCGGGGAATTCGAACGCGTTGTGGGCTCTTTTATT GAGCTCAC	PCR	this study
HS4 NotI rev	TTGCGGCCGCTTGATATCGAATTCGAGTTGGCGCGCC TGTCATTC	PCR	this study
HS4 NcoI fwd	CCTTCCATGGGGTTGTGGGCTCTTTTATTGAGCTCAC	PCR	this study
HS4 HpaI EcoRI rev	TTTTTGAATTCGAGTTAACATTCGAGTTGGCGCGCCT GTCA	PCR	this study
TRE3G Promoter fwd (LNCX)	AGCTCGTTTAGTGAACCGTCAGATC	PCR	this study
EGFP_1-rev	GCTGAACTTGTGGCCGTTTAC	PCR	this study
Mecp2 R91K fwd	CATTATCCGTGACAAGGGACCTATGTATG	PCR	this study
Mecp2 R91K rev	CATACATAGGTCCCTTGTCACGGATAATG	PCR	this study
Mecp2 R162R167K fwd	AAAGGGAGCCCCTCCAAGAGAGAGCAG	PCR	this study
Mecp2 R162R167K rev	CTTGGAGGGGCTCCCTTTCCCAGTTAC	PCR	this study
Mecp2 R91Q fwd	CATTATCCGTGACCAGGGACCTATGTATG	PCR	this study
Mecp2 R91Q rev	CATACATAGGTCCCTGGTCACGGATAATG	PCR	this study
Mecp2 R162R167Q fwd	CAAGGGAGCCCCTCCCAGAGAGAGCAG	PCR	this study
Mecp2 R162R167Q rev	CTGGGAGGGGCTCCCTTGCCCAGTTAC	PCR	this study
Mecp2 R91L fwd	CATTATCCGTGACCTGGGACCTATGTATG	PCR	this study
Mecp2 R91L rev	CATACATAGGTCCCAGGTCACGGATAATG	PCR	this study
Mecp2 R162R167L fwd	CTAGGGAGCCCCTCCCTGAGAGAGCAG	PCR	this study
Mecp2 R162R167L rev	CAGGGAGGGGCTCCCTAGCCCAGTTAC	PCR	this study
Mecp2 S80A fwd	AGAAGCCTCGGCTGCCCCCAAACAG	PCR	this study
Mecp2 S80A rev	CTGTTTGGGGGCAGCCGAGGCTTCT	PCR	this study
Mecp2 S80D fwd	AGAAGCCTCGGCTGACCCCAAACAG	PCR	this study
Mecp2 S80D rev	CTGTTTGGGGTCAGCCGAGGCTTCT	PCR	this study
Mecp2 S421A fwd	GAGGAGGCGCACTGGAAAGCGAT	PCR	this study
Mecp2 S421A rev	ATCGCTTTCCAGTGCGCCTCCTC	PCR	this study
Mecp2 S421D fwd	GAGGAGGCGATCTGGAAAGCGAT	PCR	this study
Mecp2 S421D rev	ATCGCTTTCCAGATCGCCTCCTC	PCR	this study
Mecp2 R106K fwd	CTGAAGGTTGGACAAAAAAGCTTAAAC	PCR	this study
Mecp2 R106K rev	GTTTAAGCTTTTTTGTCCAACCTTCAG	PCR	this study
Mecp2 R106Q fwd	CTGAAGGTTGGACACAAAAGCTTAAAC	PCR	this study
Mecp2 R106Q rev	GTTTAAGCTTTTGTGTCCAACCTTCAG	PCR	this study
Mecp2 R106L fwd	CTGAAGGTTGGACACTAAAGCTTAAAC	PCR	this study

Mecp2 R106L rev	GTTTAAGCTTTAGTGTCCAACCTTCAG	PCR	this study
Mecp2 R106W fwd	CTGAAGGTTGGACATGGAAGCTTAAAC	PCR	this study
Mecp2 R106W rev	GTTTAAGCTTCCATGTCCAACCTTCAG	PCR	this study
Mecp2 R106G fwd	CTGAAGGTTGGACAGGAAAGCTTAAAC	PCR	this study
Mecp2 R106G rev	GTTTAAGCTTTCCTGTCCAACCTTCAG	PCR	this study
hPRMT5 fwd	TTTTGGATCCATGGCGGCGATGGCGGTC	PCR	this study
hPRMT5 rev	AAACTCGAGTCAGAATTCGAGGCCAATGGTATATG	PCR	this study

Supplementary Table 3: Cell line characteristics.

Name	Species	Type	Genotype	Reference
C2C12	*Mus musculus*	myoblast	wild type	Yaffe and Saxel, 1977
MTF -/y	*Mus musculus*	tail fibroblast	Mecp2 -/y	Guy et al., 2001
HEK293T	*Homo sapiens*	embryonic kidney	wild type	DuBridge et al., 1987
BL21 (DE3)	*Escherichia coli*	-	F – ompT hsdSB (rB – , m~B–) gal dcm (DE3)	Studier and Moffatt, 1986

Supplementary Table 4: Primary and secondary antibody characteristics.

Reactivity	Host	Dilution	Application	Cat / clone	Company/Reference
Anti-MeCP2	rabbit	1:500	WB	-	Jost et al., 2011
Anti-MeCP2	rat	Undiluted TCSN	IF / WB	4H7, 4G10, 4E1	Jost et al., 2011
Anti-mono methyl arginine	rabbit	1:1000	WB	#8015	Cell Signaling Technology
Anti-symmetric dimethyl arginine (SDMA)	rabbit	1:1000	WB	#13222	Cell Signaling Technology
Anti-asymmetric dimethyl arginine (ADMA)	rabbit	1:1000	WB	#13522	Cell Signaling Technology
Anti-phospho MECP2 Ser80	rabbit	1:1000	WB	#P21953	Molecular Probes, Inc.
Anti-phospho MECP2 Ser421	rabbit	1:1000	WB	#PA5-35396	Thermo Fisher Scientific
Anti-phospho tyrosine	mouse	1:500	WB	#3630	Clontech Laboratories, Inc.
Anti-Myc tag	mouse	1:1000	IF	#ab32 / 9E10	abcam
Anti-rat IgG Cy3	donkey	1:300 / 1:1000	IF / WB	#712-165-153	Jackson ImmunoResearch Laboratories, inc.
Anti-mouse IgG Cy5	donkey	1:250	IF	#715-175-150	Jackson ImmunoResearch Laboratories, inc.
Anti-rabbit IgG HRP	goat	1:10000	WB	#A0545	Sigma-Aldrich, Inc.
Anti-mouse IgG HRP	sheep	1:5000	WB	#NA 931	Amersham Pharmacia Biotech

IF: immunofluorescence, WB: Western blot; HRP: horseradish peroxidase. TCSN: tissue culture supernatant

Supplementary Table 5: Imaging system characteristics.

Microscope / Company	Lasers/lamps	Filters (ex. & em. (nm))	Objectives/ lenses	Detection system	Incubation system	Application
Nikon CREST Eclipse TiE2, Nikon, Tokyo, Japan	SPECTRA X LED 470/24 nm (196 mW), 550/15 nm (260 mW), 640/30 nm (231 mW)	em.: Quadbandpass (432/25 nm; 515/25 nm; 595/25 nm; 730/70 nm)	40x air Plan Apo λ DIC (0.95 NA, 250 µm WD)	Nikon Qi2 751600 16.25 MPx	-	Fluorescence imaging + DIC
Confocal microscope Leica TCS SPEII, Wetzlar, Germany	Multicolor solid-state laser module RYBV 405 nm / 25 mW 488 nm / 10 mW	em.: DAPI: ex. 360/40, em. LP 425 GFP: ex. 470/40, em.: LP515	oil immersion 63x ACS APO CS (1.3 NA)	Leica SP-Detector adjustable in the range of 430 – 750 nm	-	Fluorescence imaging of MTF -/y cells
Widefield Axiovert 200 /Zeiss, Germany	HBO100 mercury lamp	DAPI (300-400 & 410-510); GFP (473-491 & 506-534);	oil immersion 63x Plan-Apochromat (1.4 NA)	12-bit AxioCam mRM	-	Fluorescence imaging for calculation of heterochromatin accumulation
Operetta high content screening microscopy/ PerkinElmer Life Sciences, UK	Xenon fiber-optic light source, 300 W, 360 – 640 nm continuous spectrum	DAPI: 360-400 & 410–480 GFP: 460-490 & 500–550 Cy5: 620-640 & 650-760	40x air (0.95 NA) long WD	14-bit Jenoptik CMOS	-	high content screening microscopy
Confocal microscope Leica SP5 II, Wetzlar, Germany	488 nm Argon ion laser, 633 nm HeNe gas laser 20 mW	AOBS beam splitter	HCX PL APO 63x / 1.4-0.6 oil lambda blue	2 HyD Hybrid Detectors	ACU live cell chamber (Olympus), inverse DMI 600 stand	FRAP
UltraView VoX spinning disk on an inverted Nikon Ti-E microscope / PerkinElmer	Solid state diode laser (488 nm)	488: 505-549	60x Plan-Apochromat NA 1.45 Oil	Cooled 14-bit Hamamatsu C9100-50 EMCCD	Closed live cell microscopy chamber (ACU control, Olympus)	Protein *in situ* extraction analysis
Amersham AI600 imager/GE Healthcare, Chicago, IL, USA	White light (trans) Chemiluminescence, fluorescence	Cy2: 525BP20, Cy3/ EtBr: 605BP40, Cy5: 705BP40	-	16-bit Peltier cooled Fujifilm Super CCD	-	Western blot, SDS-PAGE imaging

ex.: extinction; em.: emission; WD: working distance; LP: long pass; BP: band pass.

Supplementary table 6: Plot statistics (main figures 3 - 7).

Figure	Sample	n	Median	Mean	StDev	95% CI	*p*-value
3B	C2C12 untransfected	200	1.00	1.00	0.03	-	-
	MeCP2 wt (low)	78	1.82	1.48	0.79	0.01	-
	MeCP2 wt (high)	100	1.68	1.46	0.70	-	-
	MeCP2 3K (low)	94	1.94	1.61	1.03	0.01	4.17e-05
	MeCP2 3K (high)	81	1.72	1.66	0.76	0.01	1.06e-03
	MeCP2 3Q (low)	92	1.67	1.43	0.65	-	7.55e-07
	MeCP2 3Q (high)	75	1.50	1.37	0.61	-	5.35e-06
	MeCP2 3L (low)	112	1.46	1.22	0.53	-	<2.2e-16
	MeCP2 3L (high)	97	1.45	1.22	0.46	-	<2.2e-16
3C	MeCP2 S80A (low)	91	1.76	1.47	0.84	0.01	0.04144
	MeCP2 S80A (high)	64	1.66	1.45	0.73	0.01	0.4674
	MeCP2 S80D (low)	82	1.66	1.38	0.76	0.01	3.66e-07
	MeCP2 S80D (high)	69	1.63	1.36	0.72	0.01	0.487
	MeCP2 S421A (low)	132	1.82	1.51	0.77	-	0.9957
	MeCP2 S421A (high)	88	1.58	1.41	0.71	-	0.06161
	MeCP2 S421D (low)	112	1.89	1.37	0.71	-	0.1606
	MeCP2 S421D (high)	73	1.50	1.35	0.71	0.01	2.37e-06
3D	MeCP2 R106K (low)	49	1.17	0.87	0.26	-	<2.2e-16
	MeCP2 R106K (high)	61	1.15	1.00	0.29	-	<2.2e-16
	MeCP2 R106Q (low)	64	1.05	0.80	0.16	-	<2.2e-16
	MeCP2 R106Q (high)	55	1.02	0.82	0.12	-	<2.2e-16
	MeCP2 R106L (low)	63	1.02	0.80	0.18	-	<2.2e-16
	MeCP2 R106L (high)	74	1.01	0.79	0.15	-	<2.2e-16
	MeCP2 R106W (low)	86	1.05	0.80	0.19	-	<2.2e-16
	MeCP2 R106W (high)	55	1.04	0.83	0.18	-	<2.2e-16
	MeCP2 R106G (low)	53	1.05	0.86	0.19	-	<2.2e-16
	MeCP2 R106G (high)	54	1.04	0.83	0.19	-	<2.2e-16
4B T 1/2	MeCP2 wt	17	26.97	27.40	5.83	0.90	-
	MeCP2 3K	11	31.52	31.13	5.79	0.11	0.1003
	MeCP2 3Q	16	14.28	14.33	6.50	0.10	3.56e-06
	MeCP2 3L	17	15.19	14.80	3.92	0.06	5.742e-08
4B mobile fraction	MeCP2 wt	17	0.69	0.72	0.16	-	-
	MeCP2 3K	11	0.69	0.70	0.17	-	0.8691
	MeCP2 3Q	16	0.81	0.85	0.13	-	0.01422
	MeCP2 3L	17	0.91	0.94	0.17	-	0.001197
4C T 1/2	MeCP2 S80A	10	27.26	29.86	9.17	0.19	0.7486
	MeCP2 S80D	16	16.36	15.56	4.59	0.07	2.383e-07
	MeCP2 S421A	13	28.19	27.61	7.63	0.14	0.8047
	MeCP2 S421D	12	23.83	27.82	11.04	0.20	0.7438
4C mobile fraction	MeCP2 S80A	10	0.69	0.73	0.15	-	0.615
	MeCP2 S80D	16	0.625	0.65	0.13	-	0.1708
	MeCP2 S421A	13	0.72	0.74	0.20	-	0.7534
	MeCP2 S421D	12	0.785	0.81	0.13	-	0.07998
4D MeCP2-WT	0	48	1.00	1.00	0.00	0.00	
	90	48	0.90	0.89	0.01	0.03	
	135	48	0.83	0.83	0.01	0.04	
	165	48	0.80	0.81	0.01	0.04	
	195	48	0.77	0.78	0.02	0.04	
	225	48	0.76	0.77	0.02	0.05	
	255	48	0.75	0.75	0.02	0.05	
	285	48	0.73	0.74	0.02	0.05	
	315	48	0.72	0.73	0.02	0.05	
	345	48	0.71	0.71	0.02	0.05	
	375	48	0.69	0.70	0.02	0.05	
	405	48	0.68	0.69	0.02	0.05	
	435	48	0.67	0.68	0.02	0.05	
	465	48	0.66	0.67	0.02	0.05	
	495	48	0.65	0.66	0.02	0.05	
	525	48	0.65	0.66	0.02	0.05	
	555	48	0.64	0.65	0.02	0.05	
	585	48	0.63	0.64	0.02	0.05	
	615	48	0.62	0.63	0.02	0.05	

4D MeCP2-S80D	0	17	1.00	1.00	0.00	0.00	
	90	17	0.78	0.79	0.04	0.08	
	135	17	0.67	0.70	0.05	0.09	
	165	17	0.60	0.67	0.04	0.08	
	195	17	0.60	0.65	0.04	0.09	
	225	17	0.60	0.64	0.04	0.09	
	255	17	0.59	0.64	0.04	0.09	
	285	17	0.59	0.63	0.04	0.09	
	315	17	0.59	0.62	0.04	0.09	
	345	17	0.59	0.62	0.04	0.09	
	375	17	0.59	0.61	0.04	0.09	
	405	17	0.59	0.61	0.04	0.09	
	435	17	0.59	0.60	0.05	0.09	
	465	17	0.58	0.60	0.05	0.09	
	495	17	0.57	0.59	0.05	0.09	
	525	17	0.57	0.59	0.05	0.09	
	555	17	0.56	0.59	0.05	0.09	
	585	17	0.55	0.59	0.05	0.09	
	615	17	0.55	0.59	0.05	0.09	
4D MeCP2-3L	0	15	1.00	1.00	0.00	0.00	
	90	15	0.78	0.75	0.02	0.05	
	135	15	0.68	0.67	0.03	0.05	
	165	15	0.66	0.64	0.03	0.06	
	195	15	0.63	0.63	0.03	0.06	
	225	15	0.61	0.62	0.03	0.06	
	255	15	0.59	0.61	0.03	0.06	
	285	15	0.57	0.60	0.03	0.06	
	315	15	0.57	0.60	0.03	0.06	
	345	15	0.57	0.59	0.03	0.07	
	375	15	0.57	0.59	0.03	0.07	
	405	15	0.57	0.58	0.03	0.07	
	435	15	0.57	0.58	0.03	0.07	
	465	15	0.56	0.58	0.03	0.07	
	495	15	0.56	0.58	0.04	0.07	
	525	15	0.56	0.57	0.04	0.07	
	555	15	0.56	0.57	0.04	0.07	
	585	15	0.56	0.57	0.04	0.07	
	615	15	0.56	0.57	0.04	0.07	
4E MeCP2-WT	150 mM NaCl lysate			808.36			
	150 mM NaCl pellet			3234.22			
	450 mM NaCl lysate			6116.65			
	450 mM NaCl pellet			1461.00			
	600 mM NaCl lysate			5727.16			
	600 mM NaCl pellet			1256.47			
4E MeCP2-S80D	150 mM NaCl lysate			1002.16			
	150 mM NaCl pellet			5951.11			
	450 mM NaCl lysate			5478.47			
	450 mM NaCl pellet			3282.41			
	600 mM NaCl lysate			4749.58			
	600 mM NaCl pellet			1191.64			
4E MeCP2-3L	150 mM NaCl lysate			207.61			
	150 mM NaCl pellet			632.16			
	450 mM NaCl lysate			4899.03			
	450 mM NaCl pellet			0.00			
	600 mM NaCl lysate			6692.30			
	600 mM NaCl pellet			0.00			
5B number	C2C12 untransfected	6787	22	22.68	8.12	0.01	-
	MeCP2 wt (low)	951	22	22.59	7.83	0.02	-
	MeCP2 wt (high)	704	20	20.32	7.21	0.02	-
	MeCP2 3K (low)	851	21	21.74	7.89	0.02	0.01342
	MeCP2 3K (high)	549	19	19.12	7.24	0.02	0.002595
	MeCP2 3Q (low)	493	22	22.44	6.96	0.02	0.9712
	MeCP2 3Q (high)	310	19	19.72	6.42	0.02	0.2815
	MeCP2 3L (low)	983	23	23.31	8.09	0.02	0.02703

	MeCP2 3L (high)	604	21	21.03	7.30	0.02	0.05115
5B area	C2C12 untransfected	6787	2.10	2.37	1.16	-	-
	MeCP2 wt (low)	951	2.16	2.39	1.19	-	-
	MeCP2 wt (high)	704	2.28	2.57	1.26	-	-
	MeCP2 3K (low)	851	2.16	2.41	1.21	-	0.1795
	MeCP2 3K (high)	549	2.40	2.69	1.35	-	1.33e-12
	MeCP2 3Q (low)	493	2.16	2.40	1.14	-	0.2373
	MeCP2 3Q (high)	310	2.34	2.59	1.22	-	0.01024
	MeCP2 3L (low)	983	2.10	2.35	1.11	-	0.2251
	MeCP2 3L (high)	604	2.22	2.51	1.21	-	0.004491
5C number	MeCP2 S80A (low)	1271	21	21.51	7.72	0.01	0002977
	MeCP2 S80A (high)	793	19	19.52	7.64	0.02	0.02728
	MeCP2 S80D (low)	1414	22	22.71	8.26	0.01	0.6859
	MeCP2 S80D (high)	886	21	20.98	8.14	0.02	0.1388
	MeCP2 S421A (low)	2403	22	22.59	8.06	0.01	0.7858
	MeCP2 S421A (high)	1440	20	20.23	7.53	0.01	0.5655
	MeCP2 S421D (low)	1105	20	20.83	8.12	0.02	1.22e-07
	MeCP2 S421D (high)	840	17	18.31	7.54	0.02	3.86e-10
5C area	MeCP2 S80A (low)	1271	2.16	2.38	1.15	-	0.815
	MeCP2 S80A (high)	793	2.28	2.56	1.27	-	0.4688
	MeCP2 S80D (low)	1414	2.10	2.33	1.13	-	2.02e-10
	MeCP2 S80D (high)	886	2.16	2.49	1.31	-	7.54e-14
	MeCP2 S421A (low)	2403	2.10	2.40	1.16	-	0.5981
	MeCP2 S421A (high)	1440	2.34	2.63	1.30	-	4.46e-06
	MeCP2 S421D (low)	1105	2.22	2.53	1.29	-	< 2.2e-16
	MeCP2 S421D (high)	840	2.40	2.78	1.49	-	< 2.2e-16
5D number	MeCP2 R106K (low)	1368	23	23.46	7.62	0.01	0.004146
	MeCP2 R106K (high)	567	22	22.71	7.20	0.02	1.41e-09
	MeCP2 R106Q (low)	1434	23	23.09	8.75	0.01	0.2508
	MeCP2 R106Q (high)	375	22	22.15	7.80	0.03	2.59e-05
	MeCP2 R106L (low)	1050	24	24.24	8.26	0.02	1.72e-06
	MeCP2 R106L (high)	167	23	23.10	8.00	0.04	5.29e-06
	MeCP2 R106W (low)	1450	23	22.90	8.46	0.01	0.3898
	MeCP2 R106W (high)	738	22	21.83	7.75	0.02	4.07e-05
	MeCP2 R106G (low)	1106	22	22.04	7.66	0.01	0.08368
	MeCP2 R106G (high)	334	20	20.57	6.88	0.02	0.398
5D area	MeCP2 R106K (low)	1368	2.10	2.32	1.09	-	2.54e-10
	MeCP2 R106K (high)	567	2.10	2.37	1.11	-	< 2.2e-16
	MeCP2 R106Q (low)	1434	2.03	2.32	1.14	-	4.29e-16
	MeCP2 R106Q (high)	375	2.10	2.37	1.15	-	< 2.2e-16
	MeCP2 R106L (low)	1050	1.97	2.21	1.02	-	< 2.2e-16
	MeCP2 R106L (high)	167	1.97	2.22	1.08	-	< 2.2e-16
	MeCP2 R106W (low)	1450	2.10	2.41	1.22	-	0.4714
	MeCP2 R106W (high)	738	2.16	2.42	1.21	-	< 2.2e-16
	MeCP2 R106G (low)	1106	2.10	2.36	1.13	-	0.005653
	MeCP2 R106G (high)	334	2.16	2.44	1.19	-	2.98e-12
6C number	PRMT1 (low)	126	26	26.71	8.91	0.05	1.09e-07
	PRMT1 (high)	21	28	27.62	5.71	0.08	0.001045
6C area	PRMT1 (low)	126	1.97	2.24	1.11	-	1.2e-14
	PRMT1 (high)	21	2.00	2.20	1.07	-	0.01079
6D number	PRMT6 (low)	3146	26	26.33	8.19	0.01	< 2.2e-16
	PRMT6 (high)	682	26	26.57	8.10	0.02	< 2.2e-16
6D area	PRMT6 (low)	3146	1.91	2.15	0.99	-	< 2.2e-16
	PRMT6 (high)	682	1.91	2.13	0.98	-	< 2.2e-16
7A	PRMT1 (high), MeCP2 wt (low)	29	27	27.93	7.63	0.09	-
	PRMT1 (high), MeCP2 wt (high)	232	20	22.12	8.93	0.04	-
	PRMT1 (low), MeCP2 wt (low)	52	27.5	26.98	9.5	0.08	-
	PRMT1 (low), MeCP2 wt (high)	282	22	22.75	8.74	0.03	-
	PRMT1 (high), MeCP2 3K (low)	26	24.5	23.58	8.83	0.11	0.0806
	PRMT1 (high), MeCP2 3K (high)	144	21	22.18	9.08	0.05	0.7324
	PRMT1 (low), MeCP2 3K (low)	40	26	24.63	9.9	0.1	0.3401
	PRMT1 (low), MeCP2 3K (high)	178	20	21.31	8.33	0.04	0.07061
	PRMT1 (high), MeCP2 3Q (low)	36	22.5	22.75	6.58	0.07	0.004312
	PRMT1 (high), MeCP2 3Q (high)	226	19	20.83	7.3	0.03	0.25
	PRMT1 (low), MeCP2 3Q (low)	96	25	25.27	8.73	0.06	0.2347

	PRMT1 (low), MeCP2 3Q (high)	276	22	23	8.38	0.03	0.8832
	PRMT1 (high), MeCP2 3L (low)	34	24.5	25.71	8.97	0.1	0.2112
	PRMT1 (high), MeCP2 3L (high)	114	23	22.68	8.73	0.05	0.2948
	PRMT1 (low), MeCP2 3L (low)	110	26.5	25.55	9.27	0.06	0.4059
	PRMT1 (low), MeCP2 3L (high)	156	23	22.97	9.09	0.05	0.7617
7B	PRMT6 (high), MeCP2 wt (low)	142	27.5	27.99	7.71	0.04	-
	PRMT6 (high), MeCP2 wt (high)	318	25	25.33	8.45	0.03	-
	PRMT6 (low), MeCP2 wt (low)	818	27	27.62	7.9	0.02	-
	PRMT6 (low), MeCP2 wt (high)	92	24	24.16	7.72	0.05	-
	PRMT6 (high), MeCP2 3K (low)	102	24	24.44	9.31	0.06	0.0005301
	PRMT6 (high), MeCP2 3K (high)	162	18	18.94	8.3	0.04	6.87e-15
	PRMT6 (low), MeCP2 3K (low)	528	25	25.44	8.88	0.02	3.08e-06
	PRMT6 (low), MeCP2 3K (high)	22	22	22.14	5.72	0.08	0.2956
	PRMT6 (high), MeCP2 3Q (low)	76	27	25.93	9.09	0.07	0.1689
	PRMT6 (high), MeCP2 3Q (high)	320	25	26.07	8.99	0.03	0.3974
	PRMT6 (low), MeCP2 3Q (low)	718	26	26.93	8.28	0.02	0.04095
	PRMT6 (low), MeCP2 3Q (high)	76	28	27.84	9.88	0.07	0.01197
	PRMT6 (high), MeCP2 3L (low)	68	25	24.84	9.77	0.07	0.04254
	PRMT6 (high), MeCP2 3L (high)	240	24	23.58	8.42	0.03	0.0241
	PRMT6 (low), MeCP2 3L (low)	470	26.5	26.44	8.77	0.03	0.01003
	PRMT6 (low), MeCP2 3L (high)	268	23	24.03	8.81	0.03	0.7235
7C	PRMT1 (high), MeCP2 R106K (low)	50	24	24.26	8.66	0.08	0.03505
	PRMT1 (high), MeCP2 R106K (high)	248	24	24.32	8.38	0.03	0.002063
	PRMT1 (low), MeCP2 R106K (low)	260	25.5	25.19	7.97	0.03	0.1909
	PRMT1 (low), MeCP2 R106K (high)	440	24	24.77	8.74	0.03	0.002416
	PRMT1 (high), MeCP2 R106Q (low)	42	22	22.50	5.86	0.06	0.001865
	PRMT1 (high), MeCP2 R106Q (high)	64	23.5	23.55	7.73	0.06	0.09642
	PRMT1 (low), MeCP2 R106Q (low)	106	23.5	23.68	8.70	0.05	0.03174
	PRMT1 (low), MeCP2 R106Q (high)	60	25	25.48	7.95	0.06	0.01825
	PRMT1 (high), MeCP2 R106L (low)	86	24	24.60	6.95	0.05	0.03178
	PRMT1 (high), MeCP2 R106L (high)	152	24	25.10	7.80	0.04	0.0001461
	PRMT1 (low), MeCP2 R106L (low)	250	26.5	26.36	8.23	0.03	0.6033
	PRMT1 (low), MeCP2 R106L (high)	144	26	26.63	7.81	0.04	7.10e-06
7D	PRMT6 (high), MeCP2 R106K (low)	82	21.5	22.89	10.05	0.07	3.54e-05
	PRMT6 (high), MeCP2 R106K (high)	168	22	21.22	9.11	0.04	5.71e-06
	PRMT6 (low), MeCP2 R106K (low)	542	23.5	23.90	9.32	0.03	7.25e-16
	PRMT6 (low), MeCP2 R106K (high)	68	23	23.25	9.31	0.07	0.4788
	PRMT6 (high), MeCP2 R106Q (low)	90	25.5	25.53	9.17	0.06	0.02262
	PRMT6 (high), MeCP2 R106Q (high)	18	28	27.17	11.09	0.17	0.3492
	PRMT6 (low), MeCP2 R106Q (low)	24	28	29.42	13.89	0.18	0.8737
	PRMT6 (low), MeCP2 R106Q(high)	16	26.5	25.50	8.02	0.13	0.4435
	PRMT6 (high), MeCP2 R106L(low)	384	24	23.70	8.80	0.03	4.07e-07
	PRMT6 (high), MeCP2 R106L (high)	174	24	23.89	7.44	0.04	0.09972
	PRMT6 (low), MeCP2 R106L (low)	610	24	23.66	8.36	0.02	< 2.2e-16
	PRMT6 (low), MeCP2 R106L (high)	30	24.5	24.37	8.45	0.10	0.7843

n: number of cells; StDev: standard deviation; CI: confidence interval; p-value: in comparison to wild type MeCP2; for 4D, the SEM (Standard Error of the Mean) values are given rather than StDev.

Supplementary table 7: Post-translational modifications of MeCP2 identified by mass spectrometry analysis.

	amino acid*	modification	peptide sequence***	number of biological replicates (total)	Proteome Discoverer	MaxQuant
NTD	R9/ K12**	met	AAAAATAAAAAAPSGGGGGGEEErLEEk	5	0	5
	R9**	dimet	AAAAATAAAAAAPSGGGGGGEEEr	5	0	5
	S13**	phos	LEEKsEDQDLQGLR	6	2	6
	K42	ac	EGkHEPLQPSAHHSAEPAEAGK	2	0	2
	K61	met / dimet	HEPLQPSAHHSAEPAEAGk	2 / 5	1 / 4	2 / 4
	S68	phos	AETSESsGSAPAVPEASASPK	2	0	2
	S70	phos	AETSESSGsAPAVPEASASPK	1	1	0
	S78	phos	AETSESSGSAPAVPEAsASPK	4	3	4
	S80	phos	AETSESSGSAPAVPEASAsPK	7	7	7
	K82	met / dimet	AETSESSGSAPAVPEASASPk	2 / 4	1 / 4	2 / 4
MBD	R91	met	DrGPMYDDPTLPEGWTR	1	1	1
	R106	dimet	GPMYDDPTLPEGWTr	4	1	4
	T148	phos	VGDtSLDPNDFDFTVTGR	1	1	0
	S149	phos	VGDTsLDPNDFDFTVTGR	1	1	1
	T160	phos	VGDTSLDPNDFDFTVtGRGSPSR	3	0	3
	R162	met / dimet	VGDTSLDPNDFDFTVTGr(GSPSR)	7 / 4	6 / 2	7 / 4
ID	S164	phos	VGDTSLDPNDFDFTVTGRGsPSR	2	2	0
	S166	phos	VGDTSLDPNDFDFTVTGRGSPsR	1	1	0
	R167	met	VGDTSLDPNDFDFTVTGRGSPSr	5	2	5
	S216	phos	VLEKsPGK	1	1	1
	S229	phos	MPFQAsPGGK	5	2	5
	T240	phos	MPFQASPGGKGEGGGAtTSAQVMVIK	1	1	0
NID	K271	ac	kPGSVVAAAAAEAK	2	0	2
	S274	phos	KPGsVVAAAAAEAK	5	3	5
	K284	met / dimet	KPGSVVAAAAAEAk	1 / 4	1 / 3	1 / 4
CTD	K321	ac	EVVkPLLVSTLGEK	5	4	1
	K331	met / dimet	EVVKPLLVSTLGEk	2 / 4	1 / 4	2 / 4

NTD: N-terminal domain, MBD: Methyl-binding domain, ID: Intervening domain, NID: N-CoR interacting domain; CTD: C-terminal domain; met: methylation; dimet: dimethylation; phos: phosphorylation; ac: acetylation; *numbering according to mouse MeCP2 exon2 isoform (484 aa, uniprot Q9Z2D6-1) **only identified in mouse MeCP2 exon1 isoform (501 aa, uniprot Q9Z2D6-2) ***A peptide may bear more than one amino acid candidate for a post-translational modification. MaxQuant provides probability scores for the localization of each PTM site that can be retrieved from the data repository. Lower case indicates the modified amino acid.

Supplementary table 8: Plot statistics (supplementary figure S13).

Figure	Sample	n	Median	Mean	StDev	95% CI	*p*-value
S13A	PRMT1 (high), MeCP2 wt (low)	29	1.91	2.16	1.00	-	-
	PRMT1 (high), MeCP2 wt (high)	232	2.10	2.36	1.16	-	-
	PRMT1 (low), MeCP2 wt (low)	52	2.03	2.24	1.08	-	-
	PRMT1 (low), MeCP2 wt (high)	282	2.10	2.37	1.14	-	-
	PRMT1 (high), MeCP2 3K (low)	26	1.91	2.21	1.13	-	0.7383
	PRMT1 (high), MeCP2 3K (high)	144	2.10	2.35	1.10	-	0.9675
	PRMT1 (low), MeCP2 3K (low)	40	1.91	2.19	1.03	-	0.2078
	PRMT1 (low), MeCP2 3K (high)	178	2.16	2.42	1.11	-	0.005726
	PRMT1 (high), MeCP2 3Q (low)	36	1.91	2.21	1.02	-	0.373
	PRMT1 (high), MeCP2 3Q (high)	226	2.28	2.51	1.17	-	7.70e-14
	PRMT1 (low), MeCP2 3Q (low)	96	2.03	2.27	1.07	-	0.4513
	PRMT1 (low), MeCP2 3Q (high)	276	2.22	2.48	1.18	-	8.05e-10
	PRMT1 (high), MeCP2 3L (low)	34	1.97	2.16	1.03	-	0.7695
	PRMT1 (high), MeCP2 3L (high)	114	2.10	2.38	1.13	-	0.4875
	PRMT1 (low), MeCP2 3L (low)	110	1.91	2.22	1.17	-	0.09646
	PRMT1 (low), MeCP2 3L (high)	156	2.03	2.30	1.29	-	5.46e-05
S13B	PRMT6 (high), MeCP2 wt (low)	142	1.91	2.11	0.96	-	-
	PRMT6 (high), MeCP2 wt (high)	318	1.97	2.23	1.04	-	-
	PRMT6 (low), MeCP2 wt (low)	818	1.91	2.11	0.96	-	-
	PRMT6 (low), MeCP2 wt (high)	92	2.03	2.29	1.07	-	-
	PRMT6 (high), MeCP2 3K (low)	102	2.10	2.38	1.24	-	< 2.2e-16
	PRMT6 (high), MeCP2 3K (high)	162	2.40	2.76	1.43	-	< 2.2e-16
	PRMT6 (low), MeCP2 3K (low)	528	2.03	2.28	1.08	-	< 2.2e-16
	PRMT6 (low), MeCP2 3K (high)	22	2.10	2.34	1.06	-	0.3049
	PRMT6 (high), MeCP2 3Q (low)	76	1.97	2.23	1.12	-	0.002288
	PRMT6 (high), MeCP2 3Q (high)	320	1.97	2.23	1.09	-	0.9272
	PRMT6 (low), MeCP2 3Q (low)	718	1.97	2.19	1.03	-	9.54e-15
	PRMT6 (low), MeCP2 3Q (high)	76	1.97	2.17	0.99	-	0.001012
	PRMT6 (high), MeCP2 3L (low)	68	2.03	2.29	1.19	-	9.98e-06
	PRMT6 (high), MeCP2 3L (high)	240	2.10	2.36	1.17	-	1.18e-11
	PRMT6 (low), MeCP2 3L (low)	470	1.97	2.19	1.00	-	6.59e-12
	PRMT6 (low), MeCP2 3L (high)	268	2.10	2.35	1.11	-	0.02225
S13C	PRMT1 (high), MeCP2 R106K (low)	50	1.97	2.15	0.94	-	0.7171
	PRMT1 (high), MeCP2 R106K (high)	248	2.03	2.24	1.03	-	4.82e-08
	PRMT1 (low), MeCP2 R106K (low)	260	1.97	2.21	0.99	-	0.7667
	PRMT1 (low), MeCP2 R106K (high)	440	2.03	2.24	1.05	-	1.23e-12
	PRMT1 (high), MeCP2 R106Q (low)	42	2.10	2.36	1.21	-	0.000767
	PRMT1 (high), MeCP2 R106Q (high)	64	1.97	2.18	1.01	-	5.72e-09
	PRMT1 (low), MeCP2 R106Q (low)	106	2.03	2.28	1.13	-	0.5803
	PRMT1 (low), MeCP2 R106Q (high)	60	1.91	2.15	1.05	-	1.16E-14
	PRMT1 (high), MeCP2 R106L (low)	86	1.97	2.16	0.99	-	0.8981
	PRMT1 (high), MeCP2 R106L (high)	152	1.91	2.07	0.92	-	< 2.2e-16
	PRMT1 (low), MeCP2 R106L (low)	250	1.91	2.13	0.99	-	0.0002114
	PRMT1 (low), MeCP2 R106L (high)	144	1.91	2.11	0.96	-	< 2.2e-16
S13D	PRMT6 (high), MeCP2 R106K (low)	82	2.10	2.37	1.17	-	8.70e-13
	PRMT6 (high), MeCP2 R106K (high)	168	2.16	2.48	1.33	-	< 2.2e-16
	PRMT6 (low), MeCP2 R106K (low)	542	2.10	2.35	1.16	-	< 2.2e-16
	PRMT6 (low), MeCP2 R106K (high)	68	2.03	2.31	1.18	-	0.9811
	PRMT6 (high), MeCP2 R106Q (low)	90	1.91	2.13	1.03	-	0.4898
	PRMT6 (high), MeCP2 R106Q (high)	18	1.97	2.12	0.78	-	0.7577
	PRMT6 (low), MeCP2 R106Q (low)	24	1.94	2.20	1.21	-	0.1254
	PRMT6 (low), MeCP2 R106Q(high)	16	1.79	2.15	1.01	0.01	0.2926
	PRMT6 (high), MeCP2 R106L(low)	384	1.97	2.20	1.13	-	0.005139
	PRMT6 (high), MeCP2 R106L (high)	174	1.91	2.16	0.99	-	0.002118
	PRMT6 (low), MeCP2 R106L (low)	610	1.97	2.25	1.10	-	< 2.2e-16
	PRMT6 (low), MeCP2 R106L (high)	30	2.00	2.23	1.04	-	0.2227

n: number of cells, StDev: standard deviation, CI: confidence interval; p-value: in comparison to wild type MeCP2

2 Supplementary Figures

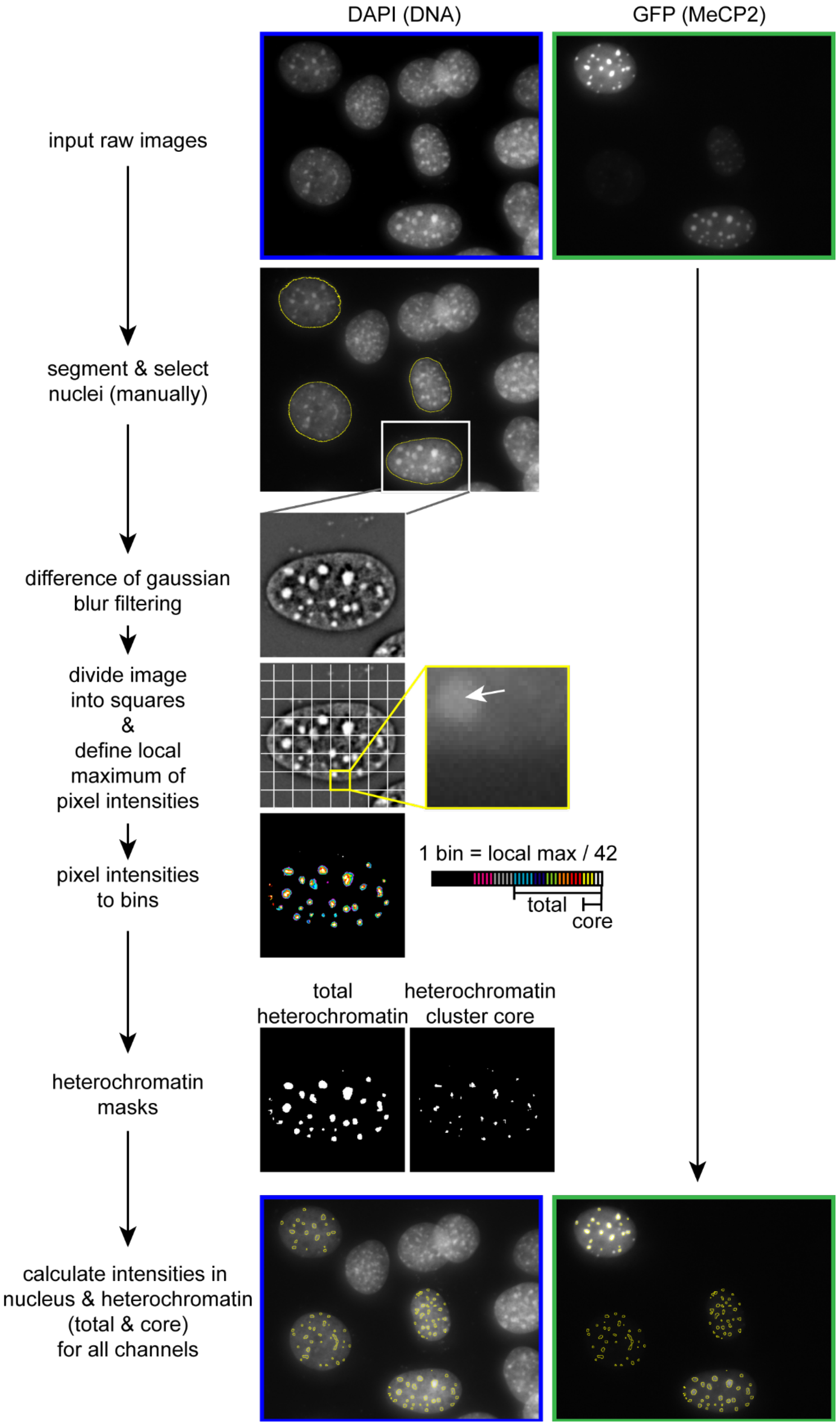

Figure S1: Image analysis pipeline using imageJ. Images were taken on a wide-field fluorescence microscope (Axiovert 200, Zeiss). Nuclei and heterochromatin segmentation were carried out based

on the DAPI channel and subsequently used for fluorescent intensity measurements on the GFP channel. Nuclei segmentation was performed manually and images were filtered using a difference of gaussian blur filter for heterochromatin segmentation. Each image was divided to squares, the local maximum of each square was determined, set as maximum for pixel intensity binning and heterochromatin clusters as well as the heterochromatin cluster cores were defined by taking a specific number of bins for the mask. The heterochromatin segmentation procedure was carried out using a self-made ImageJ macro published in (Zhang et al., 2022).

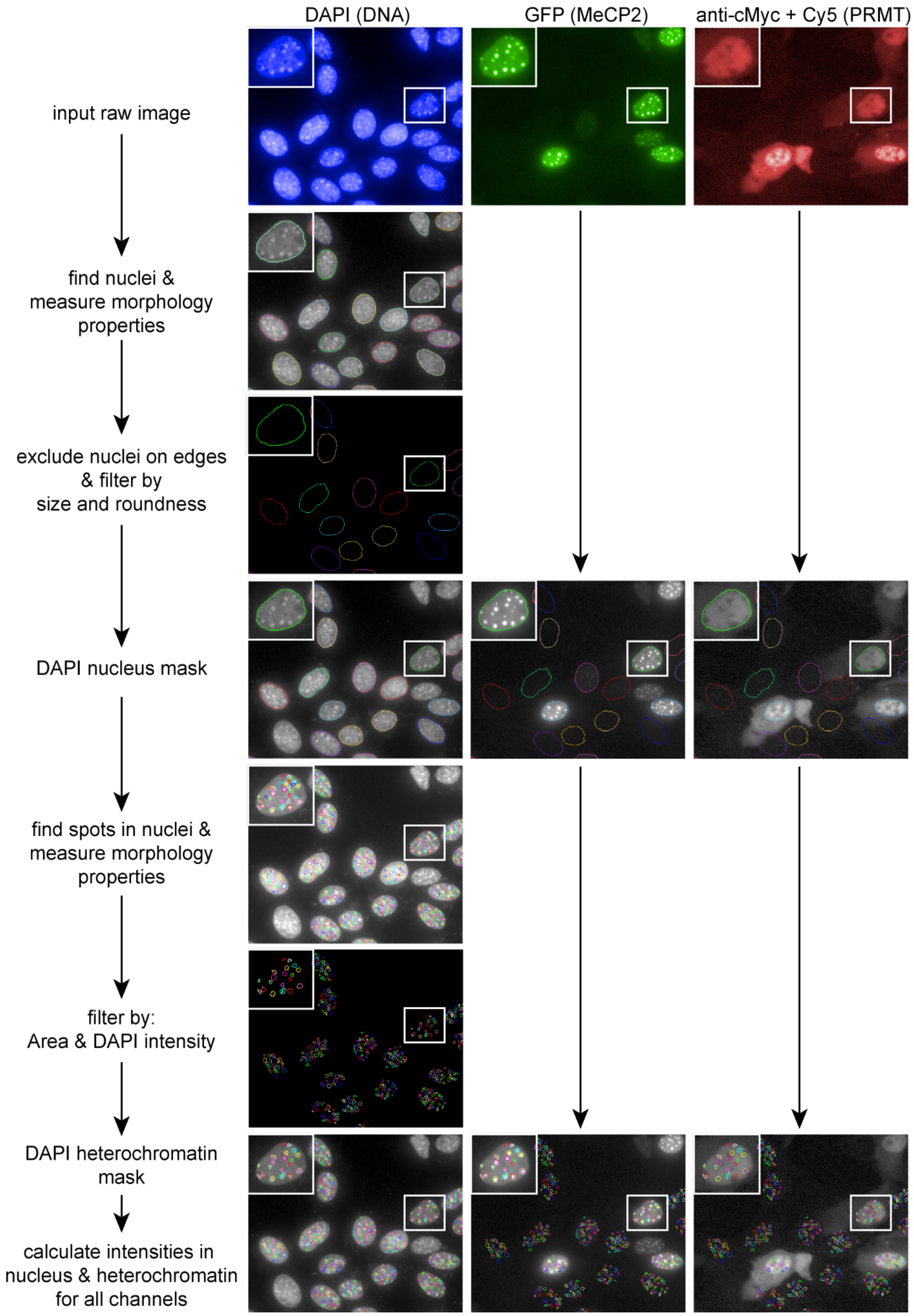

Figure S2: High content screening microscopy analysis pipeline. The analysis of high content screening microscopy images was done using the PerkinElmer Harmony software. Based on the DAPI channel, nuclei were segmented, those on edges were excluded and nuclei were filtered by size and roundness. For heterochromatin cluster segmentation in the DAPI channel, spots were found inside the nuclei and filtered by area and total DAPI intensity. The nuclei and heterochromatin mask were used to calculate fluorescent intensities in all channels.

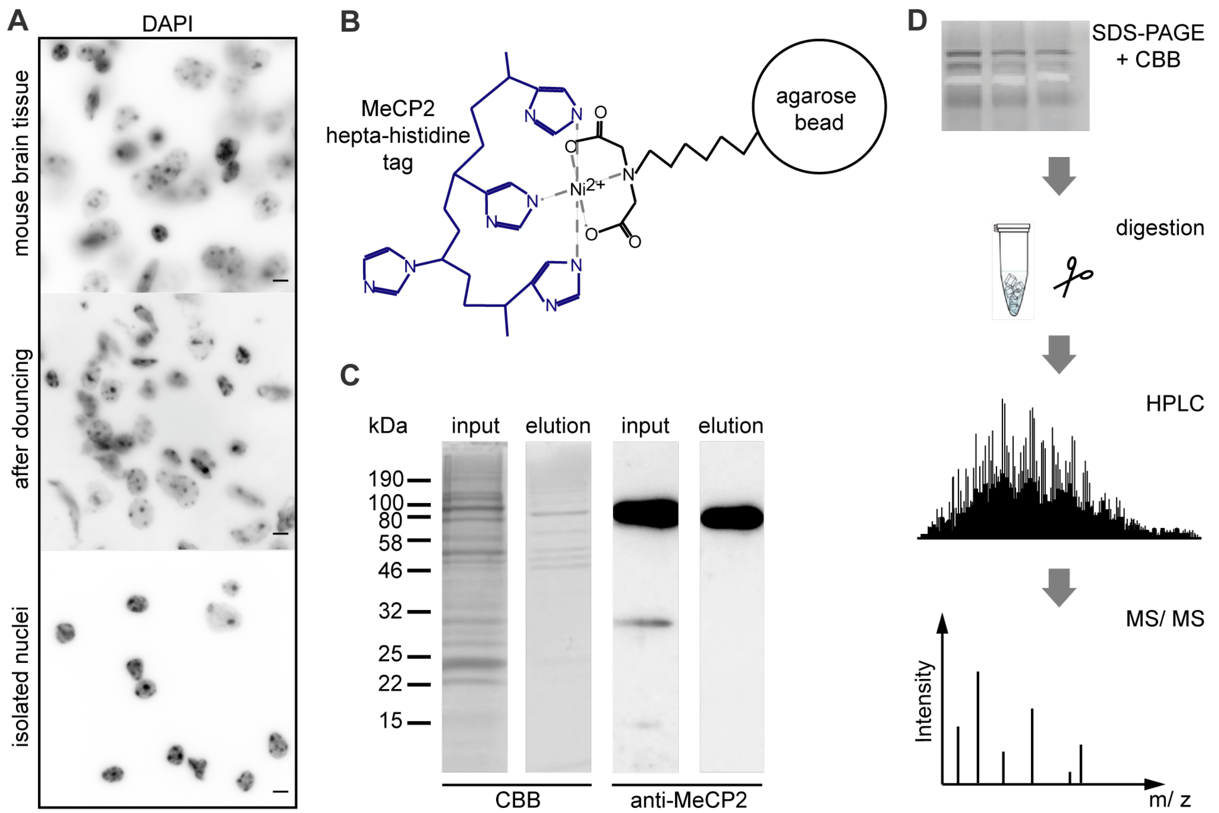

Figure S3: MeCP2 enrichment from mouse brain tissue and mass spectrometry analysis. (A) Images of DNA stained with DAPI from mouse brain tissue to the isolated nuclei. Scale bar 5 µm. (B) Scheme of the binding of MeCP2 hepta-histidine tag to Ni-IDA agarose beads. (C) Coomassie (CBB) stained SDS-PAGE and Western blot with antibodies specific to MeCP2 showing the input and elution fraction of the MeCP2 enrichment procedure. (D) Workflow of sample preparation and subsequent HPLC-MS/MS analysis involving SDS-PAGE and Coomassie staining, gel band extraction, trypsin digestion and HPLC-coupled mass spectrometry.

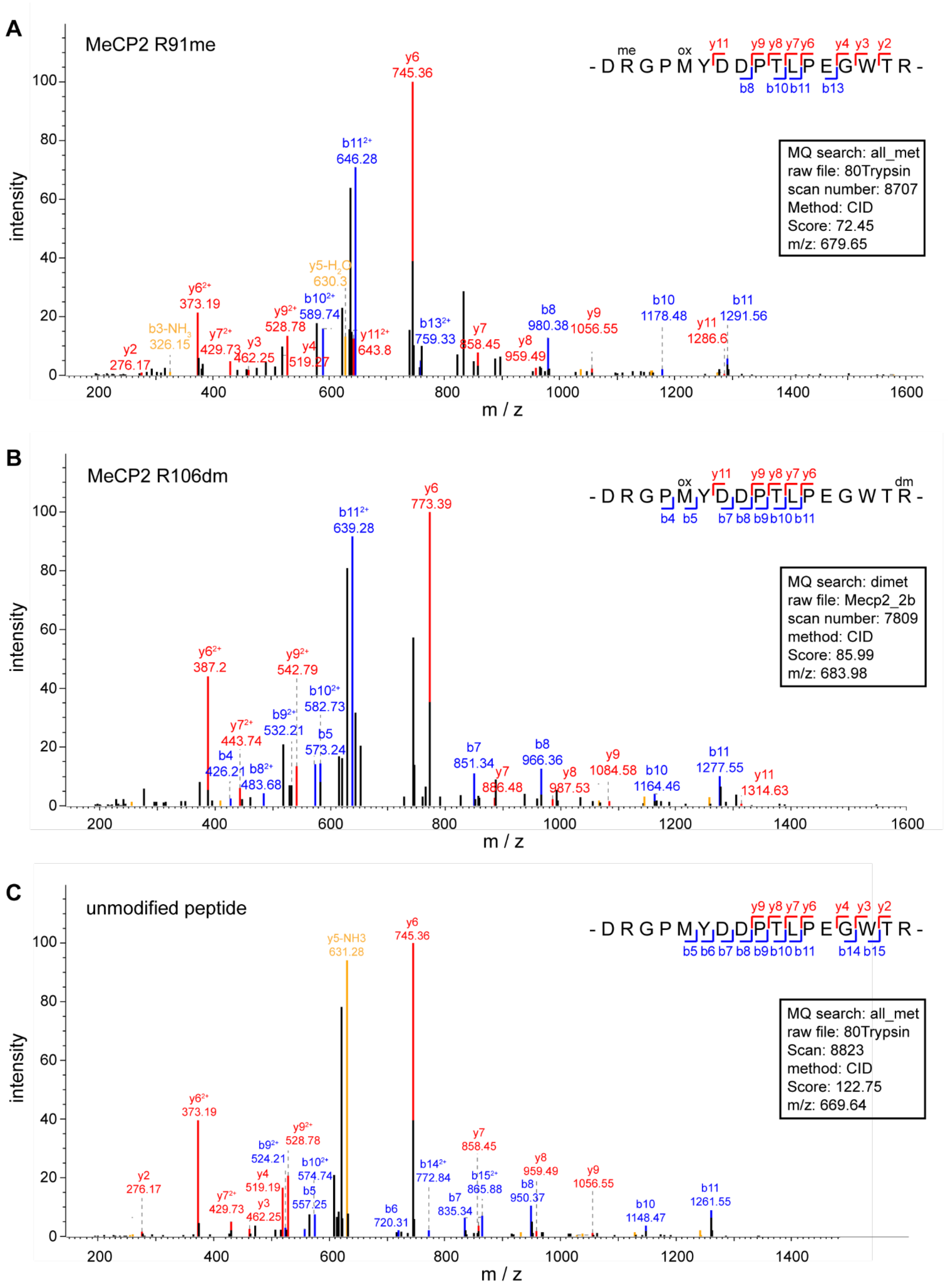

Figure S4: Manual validation of the arginine methylation sites R91 and R106 identified by mass spectrometry. Exemplary MS/MS spectra for the identification of MeCP2 R91 methylation (me) (A), MeCP2 R106 dimethylation (dm) (B) and an exemplary spectrum of the same peptide without modifications (C). The spectra were exported from the MaxQuant software (Tyanova et al., 2016) visualization tool, fragments of interest were labeled. The identified y- and b-ions are indicated on the peptide sequence and the details of the spectra are given in the black box.

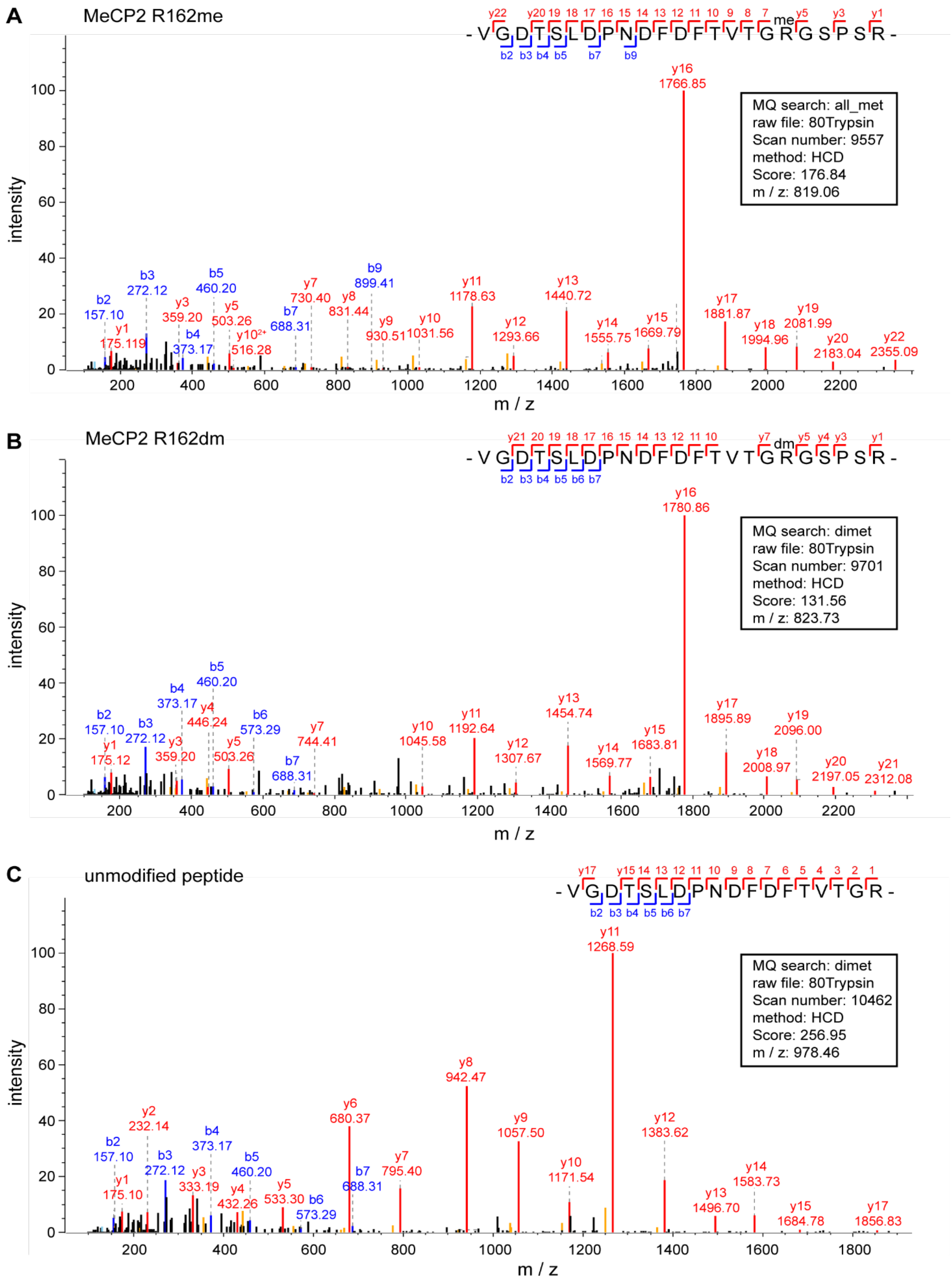

Figure S5: Manual validation of the arginine methylation site R162 identified by mass spectrometry. Exemplary MS/MS spectra for the identification of MeCP2 R162 methylation (me) (A), MeCP2 R162 dimethylation (dm) (B) and an exemplary spectrum of a peptide without the missed-cleavage and without modifications (C). The spectra were exported from the MaxQuant software (Tyanova et al., 2016) visualization tool, fragments of interest were labeled. The identified y- and b-ions are indicated on the peptide sequence and the details of the spectra are given in the black box.

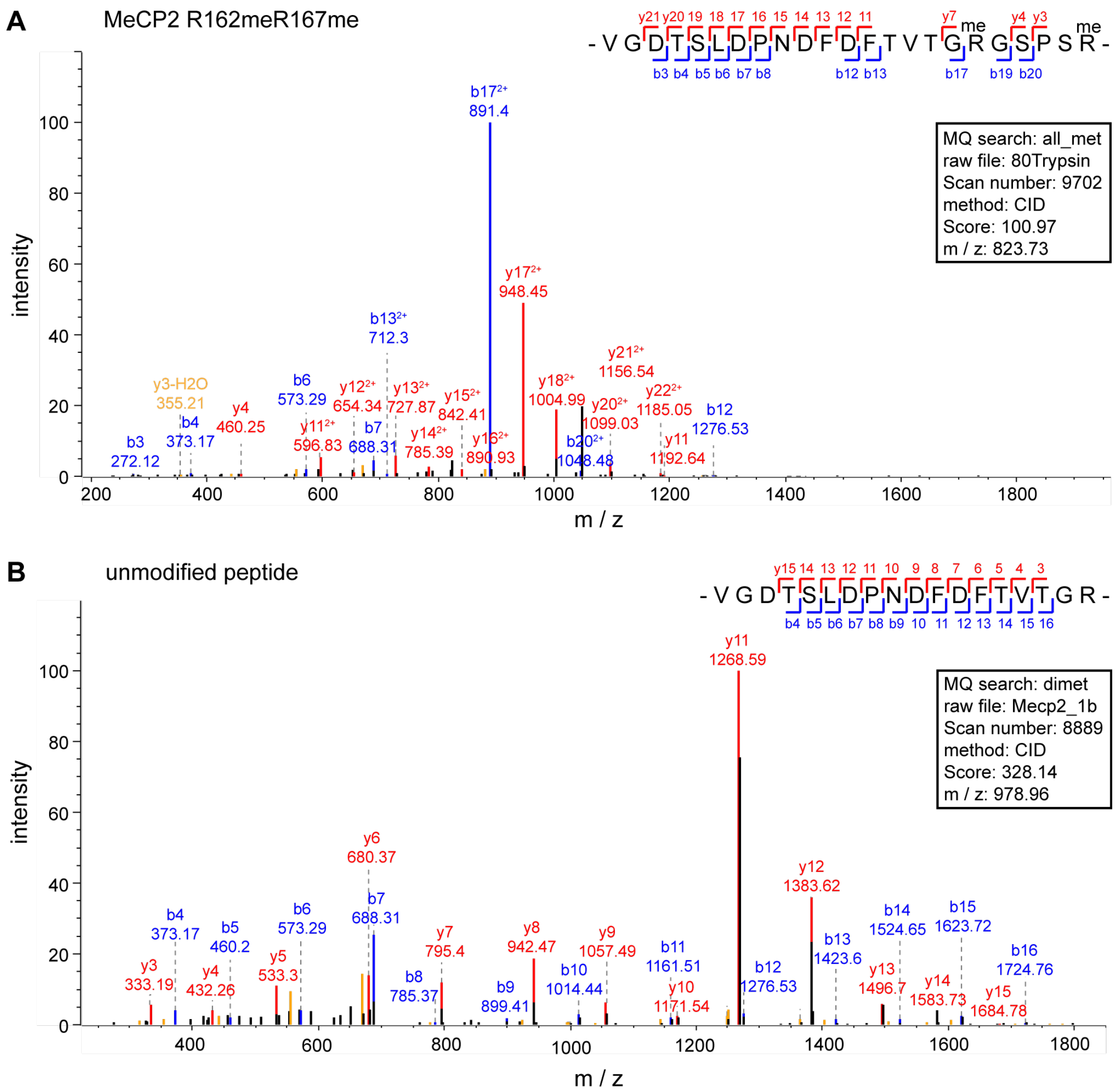

Figure S6: Manual validation of the arginine methylation site R167 identified by mass spectrometry. Exemplary MS/MS spectra for the identification of MeCP2 R167 methylation (me) (A) and an exemplary spectrum of a peptide without the missed cleavage and without modifications (B). The localization of R167 methylation cannot be determined from the spectrum shown in A, as it could also be a dimethylation on R162. The spectra were exported from the MaxQuant software (Tyanova et al., 2016) visualization tool, fragments of interest were labeled. The identified y- and b-ions are indicated on the peptide sequence and the details of the spectra are given in the black box.

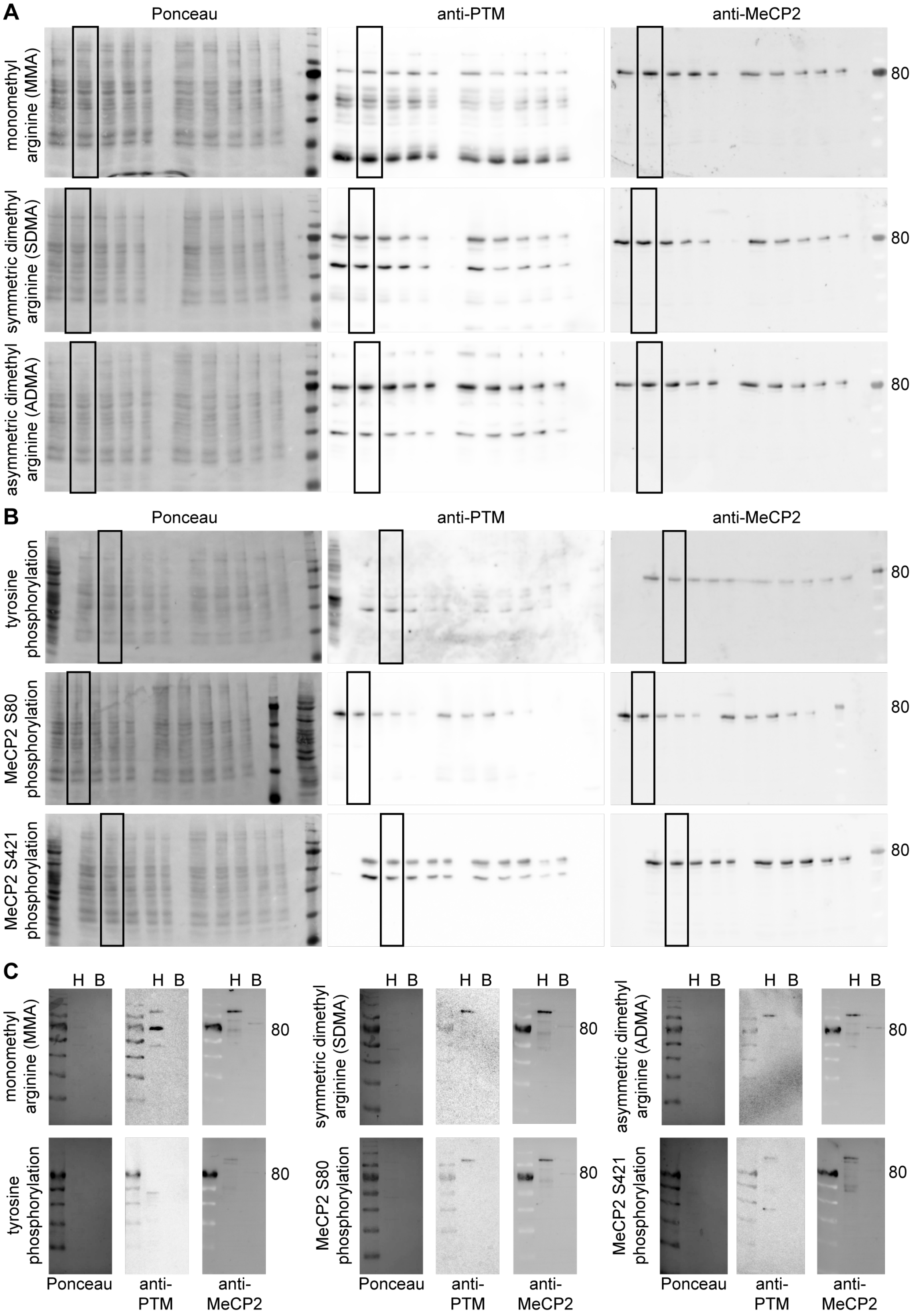
A
Ponceau
anti-PTM
anti-MeCP2
monomethyl arginine (MMA)
symmetric dimethyl arginine (SDMA)
asymmetric dimethyl arginine (ADMA)
80
B
tyrosine phosphorylation
MeCP2 S80 phosphorylation
MeCP2 S421 phosphorylation
C
H B
Ponceau
anti-PTM
anti-MeCP2

Figure S7: Western blots for detection of MeCP2 post-translational modifications on mouse brain nuclei. Western blot analysis of mouse brain nuclei extracts from 10^6 nuclei per lane tested for (A) monomethyl arginine (MMA), symmetric dimethylarginine (SDMA), asymmetric dimethyl arginine (ADMA), (B) tyrosine phosphorylation, MeCP2 S80 phosphorylation, MeCP2 S421 phosphorylation and reprobed with an antibody specific for MeCP2. Shown are the full membranes stained for total protein with Ponceau S stain, incubated with PTM specific antibodies and with MeCP2 specific antibodies. Boxes mark the lanes of interest shown in figure 1. (C) Western blot analysis of MECP2-GFP purified from human HEK cells (H) and MeCP2 purified from *E. coli* (B) probed with the same antibodies as in (A) and (B).

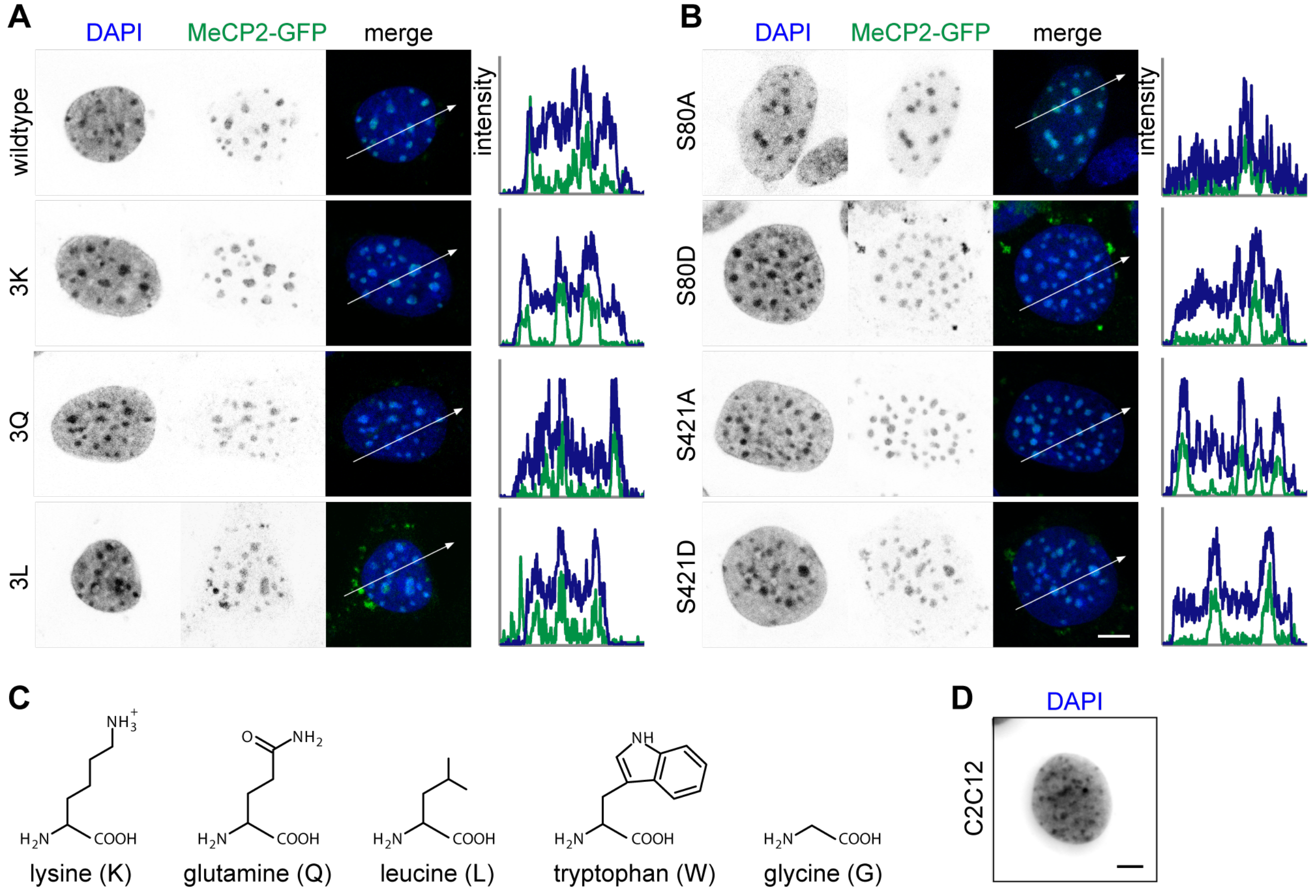

Figure S8: Subcellular localization of MeCP2-GFP mutated for arginine methylated (A) and phosphorylated (B) sites in MTF *Mecp2* -/y cells. (C) Structure of amino acids used as substitutes for methylated arginine sites. (D) DNA staining of a C2C12 mouse myoblast cell. Scale bars 5 µm.

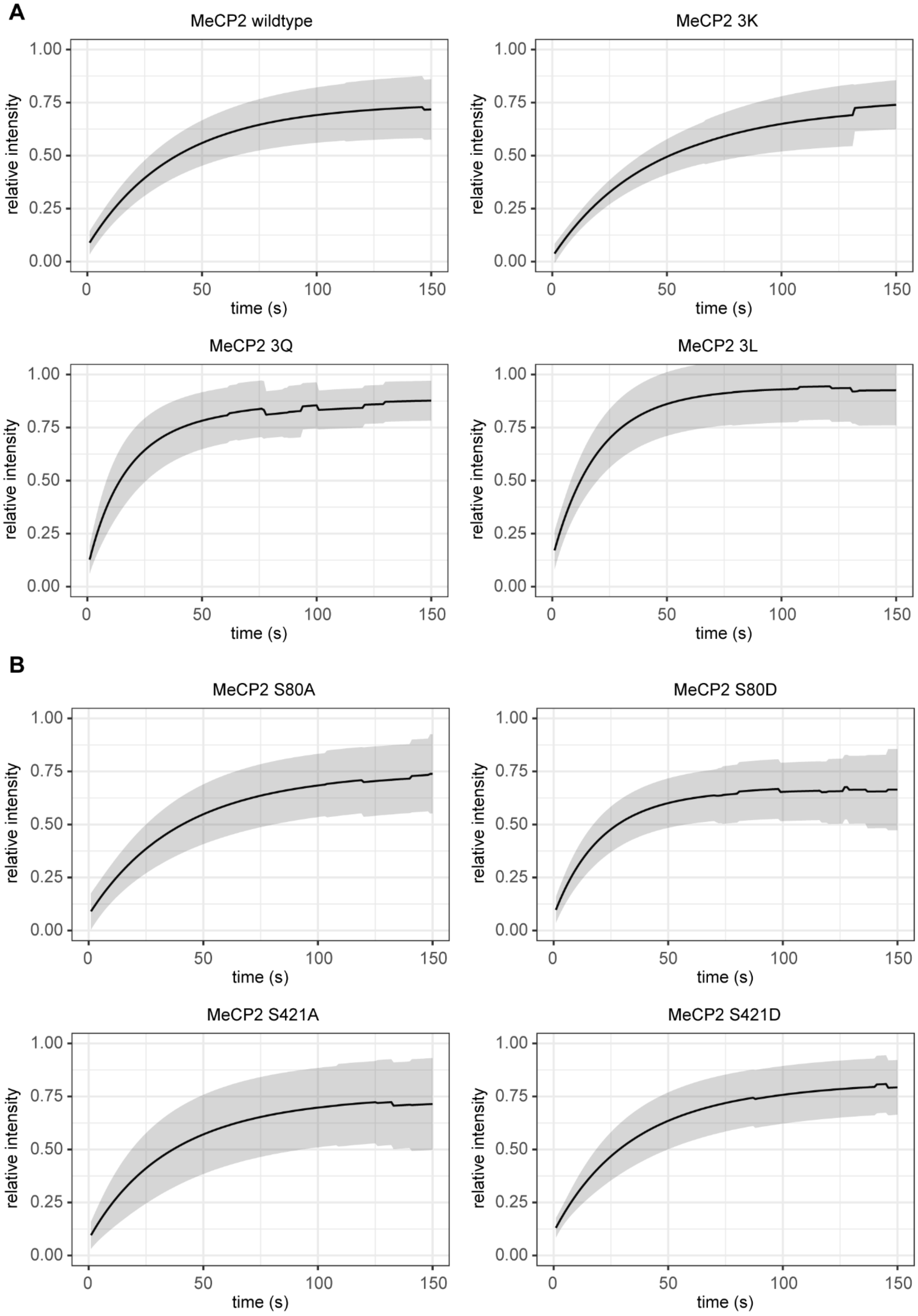

Figure S9: Fluorescence recovery after photobleaching curves of MeCP2 3x arginine methylation (A) and single serine phosphorylation mutants (B) transfected in MTF *Mecp2* -/y cells. The mean of all individual fitted curves per timepoint with the standard deviation for each individual timepoint (grey shading) is shown in the plots.

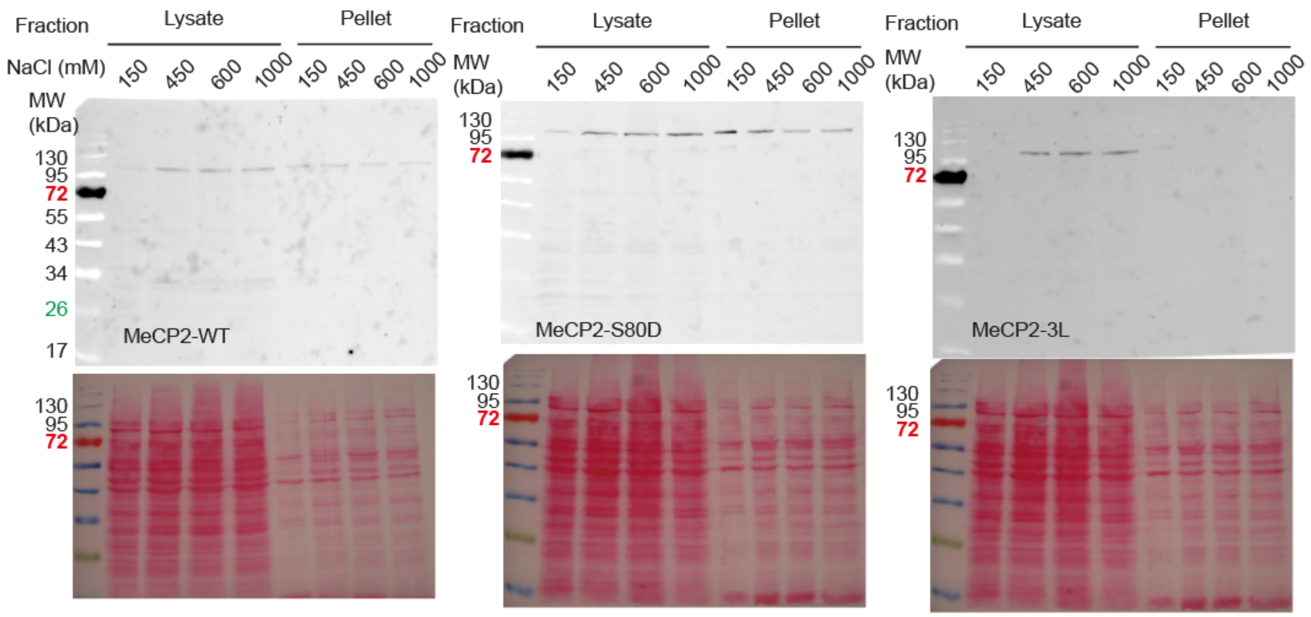

Figure S10: Western blots for detection of MeCP2 extractability. C2C21 cells were transfected with the indicated constructs and extracted using different salt conditions. Membranes were stained with Ponceau S (bottom images) and afterwards were probed with anti-MeCP2 antibodies (upper images).

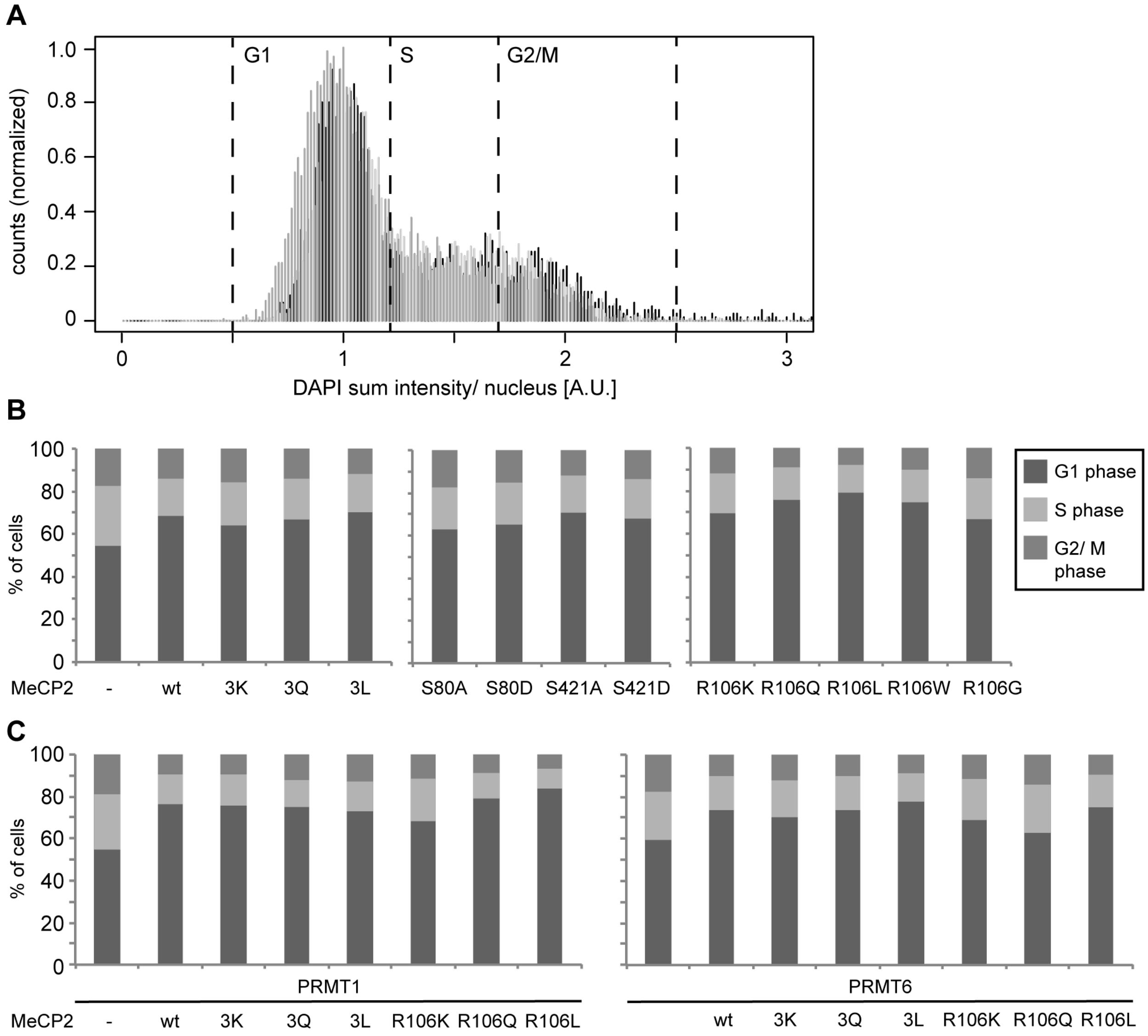

Figure S11: Cell cycle distribution analysis of mouse myoblast cells transfected with the constructs as indicated. (A) Exemplary cell cycle distribution plotted as the count of cells per DAPI sum intensity in the nucleus. The different intervals for cell cycle phases G1, S and G2/ M phase are indicated. (B) Bar diagrams depicting the percentage of cells per cell cycle phase as indicated in (A). Mock transfected cells (MeCP2 -), wild type (wt) MeCP2 and MeCP2 mutant transfected cells are shown in (B), transfections of the PRMTs 1 and 6 alone (MeCP2 -) and together with MeCP2 wild type and MeCP2 mutants are shown in (C).

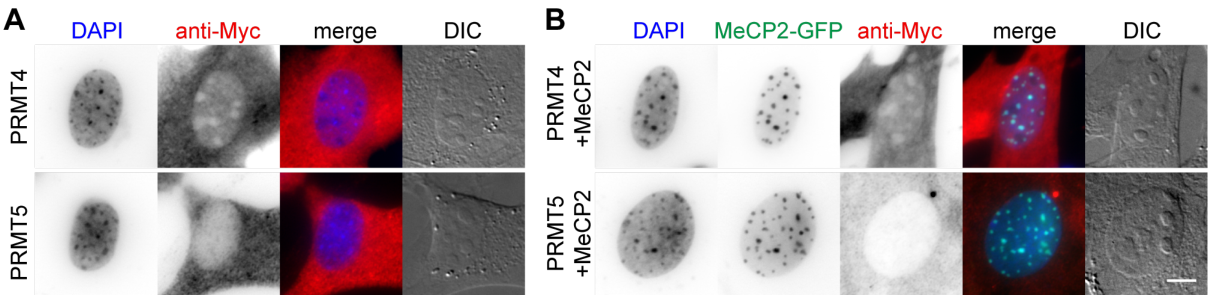

Figure S12: Subcellular localization of PRMT4 and 5 in C2C12 mouse myoblast cells in absence (A) and presence (B) of MeCP2. Scale bar 5 µm.

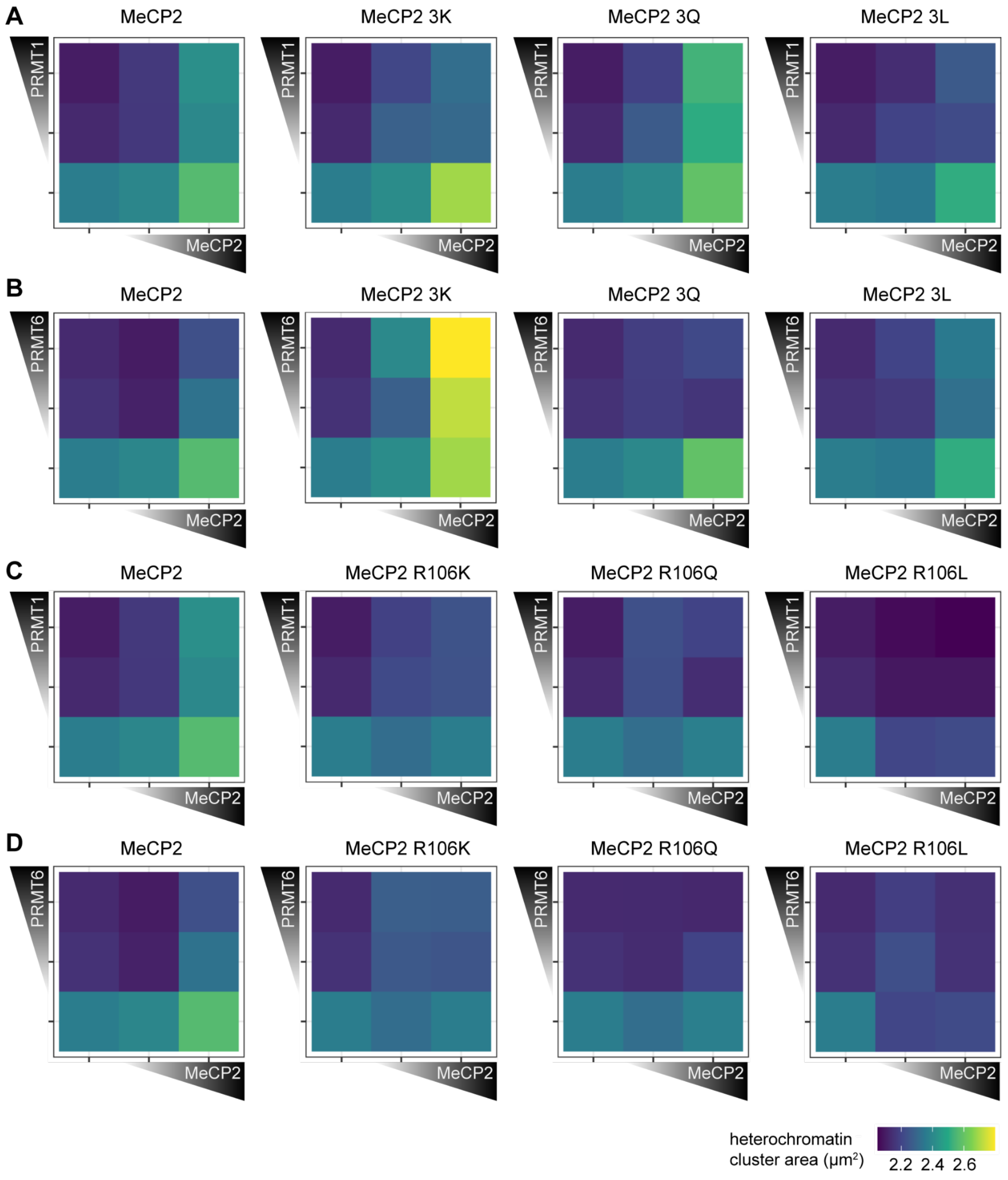

Figure S13: Heterochromatin cluster areas of C2C12 mouse myoblast cells transfected with MeCP2 mutant constructs in the presence of protein arginine methyltransferases (PRMTs) 1 and 6. Heatmaps show the heterochromatin cluster areas obtained by high-content screening microscopy of C2C12 cells cotransfected with MeCP2 3x mutants and PRMT1 (A) or PRMT6 (B) and with MeCP2 R106 mutants and PRMT1 (B) and PRMT6 (D). Cells were binned for low and high fluorescence intensity of both MeCP2 (GFP channel) and PRMT (Cy5). Heterochromatin cluster areas are shown as means of at least 26 cells from at least two biological replicates.

3 References

DuBridge, R. B., Tang, P., Hsia, H. C., Leong, P. M., Miller, J. H., and Calos, M. P. (1987). Analysis of mutation in human cells by using an Epstein-Barr virus shuttle system. *Mol. Cell. Biol.* 7, 379–387. doi:10.1128/mcb.7.1.379-387.1987.

Georgel, P. T., Horowitz-Scherer, R. A., Adkins, N., Woodcock, C. L., Wade, P. A., and Hansen, J. C. (2003). Chromatin compaction by human MeCP2. Assembly of novel secondary chromatin structures in the absence of DNA methylation. *J. Biol. Chem.* 278, 32181–32188. doi:10.1074/jbc.M305308200.

Guy, J., Hendrich, B., Holmes, M., Martin, J. E., and Bird, A. (2001). A mouse Mecp2-null mutation causes neurological symptoms that mimic Rett syndrome. *Nat. Genet.* 27, 322–326. doi:10.1038/85899.

Jost, K. L., Rottach, A., Milden, M., Bertulat, B., Becker, A., Wolf, P., Sandoval, J., Petazzi, P., Huertas, D., Esteller, M., et al. (2011). Generation and characterization of rat and mouse monoclonal antibodies specific for MeCP2 and their use in X-inactivation studies. *PLoS ONE* 6, e26499. doi:10.1371/journal.pone.0026499.

Qian, K., Huang, C. T.-L., Chen, H., Blackbourn, L. W., Chen, Y., Cao, J., Yao, L., Sauvey, C., Du, Z., and Zhang, S.-C. (2014). A simple and efficient system for regulating gene expression in human pluripotent stem cells and derivatives. *Stem Cells* 32, 1230–1238. doi:10.1002/stem.1653.

Ran, F. A., Hsu, P. D., Wright, J., Agarwala, V., Scott, D. A., and Zhang, F. (2013). Genome engineering using the CRISPR-Cas9 system. *Nat. Protoc.* 8, 2281–2308. doi:10.1038/nprot.2013.143.

Rival-Gervier, S., Lo, M. Y., Khattak, S., Pasceri, P., Lorincz, M. C., and Ellis, J. (2013). Kinetics and epigenetics of retroviral silencing in mouse embryonic stem cells defined by deletion of the D4Z4 element. *Mol. Ther.* 21, 1536–1550. doi:10.1038/mt.2013.131.

Stein, C., Riedl, S., Rüthnick, D., Nötzold, R. R., and Bauer, U.-M. (2012). The arginine methyltransferase PRMT6 regulates cell proliferation and senescence through transcriptional repression of tumor suppressor genes. *Nucleic Acids Res.* 40, 9522–9533. doi:10.1093/nar/gks767.

Studier, F. W., and Moffatt, B. A. (1986). Use of bacteriophage T7 RNA polymerase to direct selective high-level expression of cloned genes. *J. Mol. Biol.* 189, 113–130. doi:10.1016/0022-2836(86)90385-2.

Tillotson, R., Selfridge, J., Koerner, M. V., Gadalla, K. K. E., Guy, J., De Sousa, D., Hector, R. D., Cobb, S. R., and Bird, A. (2017). Radically truncated MeCP2 rescues Rett syndrome-like neurological defects. *Nature* 550, 398–401. doi:10.1038/nature24058.

Tyanova, S., Temu, T., and Cox, J. (2016). The MaxQuant computational platform for mass spectrometry-based shotgun proteomics. *Nat. Protoc.* 11, 2301–2319. doi:10.1038/nprot.2016.136.

Yaffe, D., and Saxel, O. (1977). Serial passaging and differentiation of myogenic cells isolated from dystrophic mouse muscle. *Nature* 270, 725–727. doi:10.1038/270725a0.

Zhang, H., Romero, H., Schmidt, A., Gagova, K., Qin, W., Bertulat, B., Lehmkuhl, A., Milden, M., Eck, M., Meckel, T., et al. (2022). MeCP2-induced heterochromatin organization is driven by oligomerization-based liquid-liquid phase separation and restricted by DNA methylation. *Nucleus* 13, 1–34. doi:10.1080/19491034.2021.2024691.
